# Chevalones H–M: Six New α-Pyrone Meroterpenoids from the Gorgonian Coral-Derived Fungus *Aspergillus hiratsukae* SCSIO 7S2001

**DOI:** 10.3390/md20010071

**Published:** 2022-01-14

**Authors:** Xia-Yu Chen, Qi Zeng, Yu-Chan Chen, Wei-Mao Zhong, Yao Xiang, Jun-Feng Wang, Xue-Feng Shi, Wei-Min Zhang, Si Zhang, Fa-Zuo Wang

**Affiliations:** 1 CAS Key Laboratory of Tropical Marine Bio-Resources and Ecology, Southern Marine Science and Engineering Guangdong Laboratory (Guangzhou), Guangdong Key Laboratory of Marine Materia Medica, RNAM Center for Marine Microbiology, South China Sea Institute of Oceanology, Chinese Academy of Sciences, 164 West Xingang Road, Guangzhou 510301, China; chenxiayu17@mails.ucas.ac.cn (X.-Y.C.); 18489875310@163.com (Q.Z.); weimaozhong@hotmail.com (W.-M.Z.); xy920412@sina.cn (Y.X.); wangjunfeng@scsio.ac.cn (J.-F.W.); shixuefeng@scsio.ac.cn (X.-F.S.); zhsimd@scsio.ac.cn (S.Z.); 2 University of Chinese Academy of Sciences, 19 Yuquan Road, Beijing 100049, China; 3 State Key Laboratory of Applied Microbiology Southern China, Guangdong Provincial Key Laboratory of Microbial Culture Collection and Application, Institute of Microbiology, Guangdong Academy of Sciences, 100 Central Xianlie Road, Guangzhou 510070, China; chenyc@gdim.cn (Y.-C.C.); wmzhang@gdim.cn (W.-M.Z.)

**Keywords:** coral-derived fungi, *Aspergillus hiratsukae*, meroterpenoids, antibacterial activity, cytotoxic activity

## Abstract

Six new α-pyrone meroterpenoid chevalones H–M (**1**–**6**), together with six known compounds (**7**–**12**), were isolated from the gorgonian coral-derived fungus *Aspergillus hiratsukae* SCSIO 7S2001 collected from Mischief Reef in the South China Sea. Their structures, including absolute configurations, were elucidated on the basis of spectroscopic analysis and X-ray diffraction data. Compounds **1**–**5** and **7** showed different degrees of antibacterial activity with MIC values of 6.25–100 μg/mL. Compound **8** exhibited potent cytotoxicity against SF-268, MCF-7, and A549 cell lines with IC_50_ values of 12.75, 9.29, and 20.11 μM, respectively.

## 1. Introduction

Chevalones are a class of meroterpenoids with multiple rings, polyketones and stereogenic structures. It is precisely because of this variable structure that they have a variety of biological activities. Chevalones A–D were first reported to be isolated from the soil-derived fungus *Eurotium chevalieri* and exhibited antimalarial activity, antimycobacterial activity and cytotoxicity against cancer cell lines [1]. On the other hand, chevalones were found to show synergistic effects, such as chevalone E, which enhanced the antibiotic oxacillin against methicillin-resistant *Staphylococcus aureus* [2,3], and potentiated the cytotoxic effect of doxorubicin in MDA-MB-231 breast cancer cells [4]. Chevalone C was found to show synergism with doxorubicin in A549 cells [5], and its acetylated analog also had certain cytotoxic activity against tumor cells [6]. In addition, chevalones F and G were both isolated by Paluka, but neither of them showed activity [7,8].

**Figure 1:**
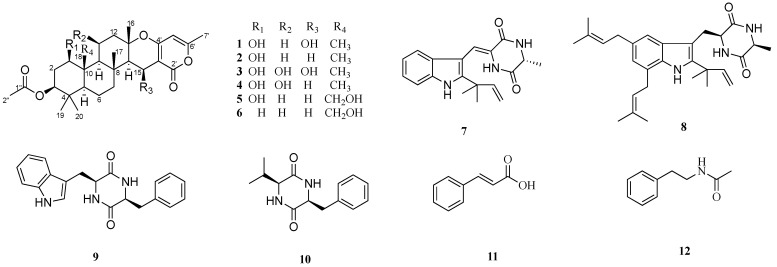
Chemical structures of compounds **1**–**12**.

Recently, we conducted NMR data analysis on the pretreated crude extracts of marine-derived fungal secondary metabolites and found that the metabolites of the coral-derived fungus *Aspergillus hiratsukae* SCSIO 7S2001 contained NMR signals with high similarity to chevalone C [1]. After further investigation of secondary metabolites of the fungus *Aspergillus hiratsukae* SCSIO 7S2001, we isolated six new α-pyrone meroterpenoid chevalones H–M (**1**–**6**), together with the six known compounds neoechinulin A (**7**) [9], echinuline (**8**) [10], isorugulosuvine (**9**) [11], cyclo(L-Phe-L-Val) (**10**) [12], *trans*-cinnamic acid (**11**) [13], and *N*-phenethylacetamide (**12**) [14], by comparison with the reported literature data (Figure 1). Herein, we report the isolation, structural elucidation and bioactivities of these compounds.

## 2. Results and Discussion

**Figure 2:**
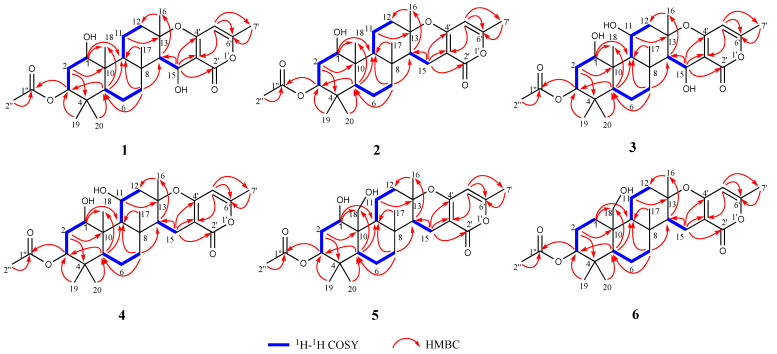
Key HMBC and COSY correlations of compounds **1**–**6**.

**Figure 3:**
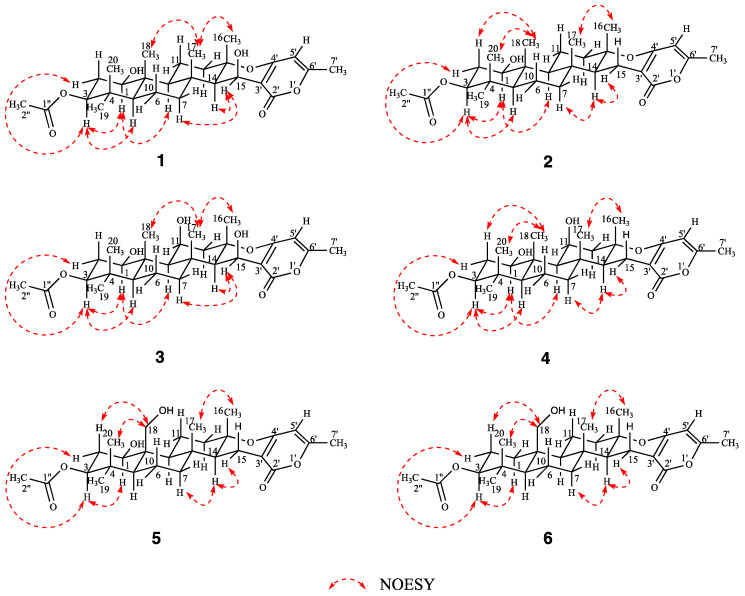
Key NOESY correlations of compounds **1**–**6**.

**Figure 4:**
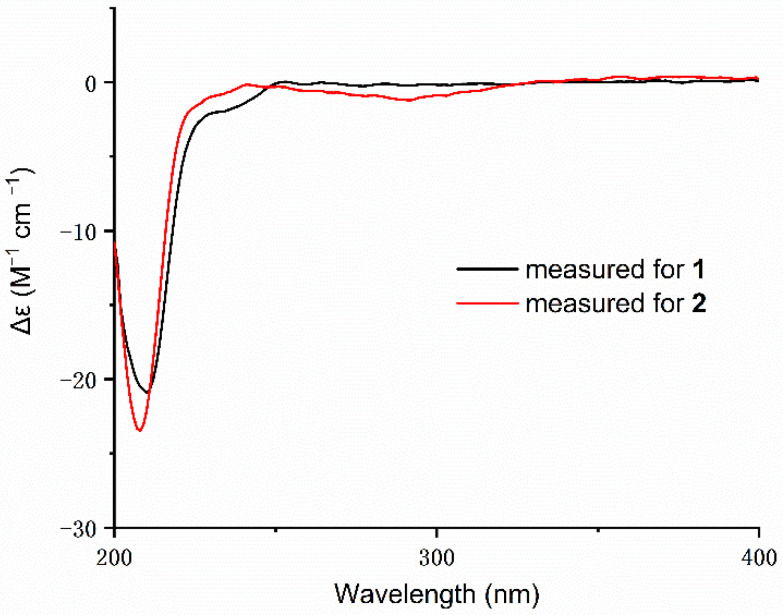
Experimental ECD spectra of compounds **1** and **2**.

**Figure 5:**
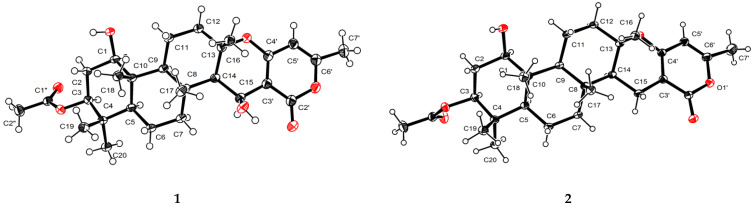
X-ray ORTEP diagram of compounds **1** and **2**.

Compound **1** was obtained as colorless crystals, and its molecular formula was determined to be C_28_H_40_O_7_ by HRESIMS (Figure S7) ion at *m*/*z* 511.2673 [M + Na]^+^ (calcd for C_28_H_40_O_7_Na, 511.2672), indicating nine degrees of unsaturation. The ^1^H NMR spectrum (Table 1) revealed the presence of seven methyl proton signals at *δ*_H_ 0.85 (3H, s), 0.86 (3H, s), 0.95 (3H, s), 1.33 (3H, s), 1.57 (3H, s), 2.05 (3H, s), and 2.19 (3H, s) and 10 diastereotopic methylene proton signals *δ*_H_ 1.18–1.21 (1H, m), 1.58–1.60 (1H, m), 1.60–1.62 (1H, m), 1.64–1.66 (1H, m), 1.66–1.68 (1H, m), 1.69–1.72 (1H, m), 1.89 (1H, *J* = 12.3, 4.5 Hz), 2.02 (1H, *J* = 11.9, 3.1 Hz), 2.16–2.18 (1H, m), and 2.65–2.69 (1H, m), seven methine proton signals *δ*_H_ 0.76 (1H, dd, *J* = 12.1, 2.5 Hz), 1.09 (1H, dd, *J* = 12.1, 2.4 Hz), 1.42 (1H, d, *J* = 4.1 Hz), 1.45 (1H, dd, *J* = 12.9, 4.7 Hz), 3.54 (1H, dd, *J* = 11.4, 4.6 Hz), 4.48 (1H, dd, *J* = 12.2, 4.4 Hz), and 4.89 (1H, d, *J* = 4.0 Hz), and the ^13^C NMR and DEPT data (Table 1) of **1** showed the presence of 28 carbon resonances, including seven methyls, five methylenes, seven methines, and nine quaternary carbons (two carbonyl carbons, three olefinic carbons and four quaternary carbons), which revealed that its structure possessed great similarity to the known meroterpenoid chevalone B [1]. By comparing their spectroscopic data with published literature values, the main difference was the hydroxylation of C-1 and C-15, which was deduced by the carbon chemical shifts. The position of C-1 could be proven via a series of mutually coupled resonances H-1/H_2_-2/H-3, H-5/H-6/H-7, H-9/H-11/H-12 and H-15 and H-16 in the COSY spectrum, respectively, along with the HMBC correlation from H-3 to C-1″; C-15 was also proven by combining the COSY and HMBC in H-14/H-15 and H-15 to C-2′ (Figure 2). The relative configuration of **1** was determined by NOESY correlations (Figure 3) of H-1/H-3, H-3/H-5, H-1/H-9, H-7/H-15, H-15/H-14, H-16/H-17 and H-17/H-18. Absolute configuration of **1** was confirmed by the experimental ECD of **1** (Figure 4) and single-crystal X-ray diffraction (Figure 5). Therefore, compound **1** was named chevalone H.

Compound **2** was obtained as colorless crystals, and its molecular formula C_28_H_40_O_6_ was deduced from the HRESIMS *m*/*z* 473.2907 [M + H]^+^ (calcd for C_28_H_41_O_6_, 473.2903), implying nine degrees of unsaturation. The ^1^H and ^13^C NMR spectra (Table 1) of **2** showed a high similarity to those of **1**, except for the lack of a hydroxyl group at C-15 (*δ*_C_ 17.0), which was supported by the HMBC correlation from the methylene proton H_2_-15 to carbonyl carbon C-2′. The relative stereochemistry was determined by a combination of the coupling constant and analysis of the NOESY spectrum, which was similar to those of **1**. In addition, the experimental ECD of **2** displayed good agreement with the experimental ECD of **1** (Figure 4). Further configuration of **2** was proven by single-crystal X-ray diffraction experimental data (Figure 5). The absolute configuration of **2** was assigned as 1*R*,3*S*,5*S*,8*R*,9*S*,10*R*,13*S*,14*S*. On the basis of the above evidence, **2** was a new α-pyrone meroterpenoid and named chevalone I. 

Compound **3** was obtained as a white solid, and its molecular formula C_28_H_40_O_8_ was deduced from the HRESIMS *m*/*z* 527.2609 [M + Na]^+^ (calcd for C_28_H_40_O_8_Na, 527.2621), implying nine degrees of unsaturation. The NMR spectra (Table 1) of **3** were similar to those of **1**, except for the presence of a secondary alcohol at C-11 (*δ*_C_ 70.0), which was supported by a series of COSY correlations between H-9/H-11/H-12. The relative stereochemistry was determined by a combination of the coupling constant and analysis of the NOESY spectrum, which was similar to those of **1**. The *J* values of 3.1 Hz for the coupling of H-9 to H-11 supported the axial position of H-11, indicating the *cis* ring junction. In addition, the experimental ECD of **3** displayed good agreement with the experimental ECD of **1** and **2** (Figure S73). The absolute configuration of **3** was assigned as 1*R*,3*S*,5*S*,8*R*,9*S*,10*S*,11*S*,13*S*,14*R*,15*S*. Thus, **3** was identified as a hydroxy derivative of **1** and named chevalone J.

**Table 1:** ^1^H NMR (700 MHz) and ^13^C NMR (176 MHz) data for compounds **1**–**3**.

NO.	1 ^a^		2 ^a^		3 ^a^	
	*δ*_C_ Type	*δ*_H_ (*J* in Hz)	*δ*_C_ Type	*δ*_H_ (*J* in Hz)	*δ*_C_ Type	*δ*_H_ (*J* in Hz)
1	78.1 CH	3.51 m	77.7 CH	3.54 dd (11.4, 4.6)	76.4 CH	3.63 dd (11.2, 4.8)
2	34.5 CH_2_	1.69–1.72 m	34.5 CH_2_	1.67–1.71 m	33.5 CH_2_	1.93–1.95 m
		1.89 dt (12.3, 4.5)		1.90 dt (12.5, 4.4)		1.80–1.82 m
3	77.3 CH	4.47 dd (12.2, 4.4)	77.2 CH	4.48 dd (12.2, 4.3)	77.3 CH	4.45 dd (12.3, 4.2)
4	37.9 C		37.9 C		37.7 C	
5	53.3 CH	0.74–0.78 m	53.3 CH	0.76 dd (12.1, 2.5)	54.4 CH	0.80 dd (12.4, 2.4)
6	17.4 CH_2_	1.58–1.60 m	17.6 CH_2_	1.56–1.58 m	18.1 CH_2_	1.06–1.08 m
		1.66–1.68 m		1.61–1.63 m		1.23–1.25 m
7	40.5 CH_2_	1.18–1.21 m	40.8 CH_2_	0.98–1.04 m	42.0 CH_2_	1.18–1.20 m
		2.16–2.18 m		1.85 dt (12.9, 3.4)		2.15–2.17 m
8	39.6 C		38.2 C		39.6 C	
9	61.6 CH	1.04 dd (11.7, 2.4)	61.1 CH	1.10 dd (12.1, 2.4)	61.5 CH	0.88 d (3.1)
10	43.5 C		43.5 C		44.1 C	
11	21.6 CH_2_	1.60–1.62 m	21.4 CH_2_	1.43–1.45 m	70.0 CH	4.79 dt (5.4, 2.7)
		2.65–2.69 m		2.64–2.70 m		
12	41.8 CH_2_	1.64–1.66 m	40.6 CH_2_	1.63–1.65 m	48.7 CH_2_	1.87–1.89 m
		2.02 dt (11.9, 3.1)		2.00–2.04 m		2.28–2.30 m
13	81.9 C		80.4 C		81.3 C	
14	56.5 CH	1.42 d (4.1)	52.1 CH	1.46 d (4.5)	56.6 CH	1.41 d (4.0)
15	60.5 CH	4.89 d (4.0)	17.0 CH_2_	2.13 dd (16.7, 13.0)	60.2 CH	4.95 d (4.0)
				2.42 dd (16.7, 4.7)		
16	22.1 CH_3_	1.57 s	20.6 CH_3_	1.19 s	23.1 CH_3_	1.77 s
17	18.3 CH_3_	1.33 s	16.4 CH_3_	0.90 s	19.6 CH_3_	1.69 s
18	12.4 CH_3_	0.95 s	12.4 CH_3_	0.92 s	14.9 CH_3_	1.29 s
19	28.0 CH_3_	0.85 s	27.9 CH_3_	0.84 s	27.8 CH_3_	0.84 s
20	16.2 CH_3_	0.86 s	16.1 CH_3_	0.85 s	16.0 CH_3_	0.88 s
2′	165.6 C		165.6 C		165.6 C	
3′	101.4 C		97.9 C		101.4 C	
4′	163.4 C		163.5 C		163.0 C	
5′	101.1 CH	5.73 s	100.8 CH	5.68 s	101.0 CH	5.73 s
6′	161.6 C		159.9 C		161.7 C	
7′	20.0 CH_3_	2.19 s	19.9 CH_3_	2.17 s	20.0 CH_3_	2.19 s
1″	171.0 C		171.0 C		171.0 C	
2″	21.3 CH_3_	2.05 s	21.3 CH_3_	2.05 s	21.3 CH_3_	2.06 s

^a^ The solvent was CDCl_3_.

Compound **4** was obtained as a white solid, and its molecular formula C_28_H_40_O_7_ was deduced from the HRESIMS *m*/*z* 489.2857 [M + H]^+^ (calcd for 489.2852), implying nine degrees of unsaturation. The NMR spectra (Table 2) of **4** indicated the same skeleton as **3**, except for the appearance of a methylene at C-15 (*δ*_C_ 17.2), which was deduced by the DEPT spectrum and HMBC correlation from H-15 to C-2′. The relative stereochemistry was determined by a combination of the coupling constant and analysis of the NOESY spectrum, which was similar to those of **3**. The *J* values of 2.9 Hz for the coupling of H-9 to H-11 supported the axial position of H-11, indicating the *cis* ring junction. In addition, the experimental ECD of **4** displayed good agreement with the experimental ECD of **1** and **2** (Figure S74). The absolute configuration of **4** was assigned as 1*R*,3*S*,5*S*,8*R*,9*S*,10*R*,11*S*,13*S*,14*S*. Thus, **4** was named chevalone K.

Compound **5** was obtained as a white solid, and its molecular formula C_28_H_40_O_7_ was deduced from the HRESIMS *m*/*z* 489.2839 [M + H]^+^ (calcd for 489.2852), implying nine degrees of unsaturation. Comparison of the spectroscopic data of **5** and **2** showed that they share a similar chevalone skeleton, except that the NMR resonances at C-18 were replaced by hydroxymethyl groups (*δ*_C_ 63.5) (Table 2). The relative stereochemistry was determined by a combination of the coupling constant and analysis of the NOESY spectrum, which was similar to those of **2**. Similarly, the experimental ECD of **5** displayed good agreement with the experimental ECD of **1** and **2** (Figure S75), which also assigned its absolute configuration. On the basis of the above evidence, **5** was a new meroterpenoid and we named it chevalone L.

Compound **6** was obtained as a white solid, and its molecular formula C_28_H_40_O_6_ was deduced from the HRESIMS *m*/*z* 473.2892 [M+H]^+^(calcd for 473.2903), implying nine degrees of unsaturation. The ^1^H and ^13^C NMR spectra of **6** (Table 2) were similar to those of **5**, except for the lack of a hydroxyl group at C-1 (*δ*_C_ 63.5), which was deduced by the DEPT NMR and COSY correlation between H-1/H-2/H-3. The relative stereochemistry was determined by a combination of the coupling constant and analysis of the NOESY spectrum, which was similar to those of **5**. In addition, the experimental ECD of **6** displayed good agreement with the experimental ECD of **1** and **2** (Figure S76). The absolute configuration of **6** was assigned as 3*S*,5*S*,8*R*,9*S*,10*R*,13*S*,14*S* and named chevalone M.

**Table 2:** ^1^H NMR (700 MHz) and ^13^C NMR (176 MHz) data for compounds **4**–**6**.

NO.	4 ^a^		5 ^b^		6 ^b^	
	*δ*_C_ Type	*δ*_H_ (*J* in Hz)	*δ*_C_ Type	*δ*_H_ (*J* in Hz)	*δ*_C_ Type	*δ*_H_ (*J* in Hz)
1	76.7 CH	3.59 dd (11.2, 4.9)	78.9 CH	3.82 dd (11.1, 5.1)	33.7 CH_2_	1.73–1.75 m
						0.93–0.95 m
2	34.0 CH_2_	1.82–1.84 m	34.7 CH_2_	1.95–1.98 m	24.7 CH_2_	1.64–1.69 m
		1.89 m		1.98–2.00 m		
3	79.0 CH	4.48 dd (12.3, 4.3)	77.2 CH	4.44 dd (12.1, 4.7)	82.4 CH	4.49 dd (11.8, 4.7)
4	38.7 C		37.5 C		38.7 C	
5	55.0 CH	0.91 dd (6.3, 1.8)	53.8 CH	0.85–0.87 m	57.3 CH	1.07–1.09 m
6	19.2 CH_2_	1.67–1.69 m	17.9 CH_2_	1.53–1.55 m	18.6 CH_2_	1.56–1.60 m
		1.76 dd (12.8, 3.7)		1.61–1.63 m		2.16 dd (16.6, 13.0)
7	43.5 CH_2_	1.09–1.12 m	41.8 CH_2_	1.05–1.07 m	42.7 CH_2_	1.14–1.18 m
		1.80–1.82 m		1.93 dt (13.0, 3.5)		1.89–1.94 m
8	39.0 C		38.4 C		38.6 C	
9	61.6 CH	1.08 d (2.9)	62.8 CH	1.18–1.20 m	62.4 CH	1.06–1.07 m
10	45.2 C		47.4 C		43.3 C	
11	70.6 CH	4.84–4.86 m	24.4 CH_2_	2.04–2.06 m	22.8 CH_2_	1.88–1.90 m
				2.68–2.70 m		
12	47.9 CH_2_	1.90–1.94 m	41.7 CH_2_	1.51–1.53 m	42.6 CH_2_	2.01–2.03 m
		2.24–2.26 m		2.00–2.02 m		1.51–1.53 m
13	81.5 C		80.5 C		82.3 C	
14	53.2 CH	1.56 dd (12.8, 4.7)	52.5 CH	1.44 dd (12.9, 4.8)	53.5 CH	1.50–1.51 m
15	17.4 CH_2_	2.40 dd (16.6, 4.8)	17.2 CH_2_	2.16–2.18m	17.8 CH_2_	2.39 dd (16.7, 4.7)
				2.43 dd (16.7, 4.8)		1.28–1.32 m
16	22.0 CH_3_	1.42 s	20.1 CH_3_	1.21 s	20.6 CH_3_	1.23 s
17	18.2 CH_3_	1.27 s	15.5 CH_3_	1.10 s	16.1 CH_3_	1.10 s
18	15.3 CH_3_	1.28 s	63.5 CH_2_	4.27 d (12.1)	62.1 CH_2_	3.85 d (12.0)
				3.90 d (12.2)		3.97 d (12.0)
19	28.0 CH_3_	0.84 s	28.2 CH_3_	0.84 s	29.1 CH_3_	0.89 s
20	16.3 CH_3_	0.89 s	16.2 CH_3_	0.81 s	17.3 CH_3_	0.87 s
2′	167.5 C		165.5 C		167.6 C	
3′	98.7 C		97.8 C		98.7 C	
4′	165.3 C		163.5 C		165.7 C	
5′	102.1 CH	5.89 s	100.8 CH	5.68 s	102.1 CH	5.89 s
6′	161.8 C		160.0 C		161.8 C	
7′	19.5 CH_3_	2.20 s	19.9 CH_3_	2.19 s	19.5 CH_3_	2.20 s
1″	172.6 C		171.1 C		172.8 C	
2″	21.0 CH_3_	2.04 s	21.3 CH_3_	2.06 s	21.1 CH_3_	2.03 s

^a^ The solvent was CD_3_OD, ^b^ the solvent was CDCl_3_.

The antibacterial activity of compounds **1**–**12** against *M. lutea*, *K. pneumoniae*, methicillin-resistant *Staphylococcus aureus*, and *Streptococcus faecalis* was evaluated with the broth dilution assay [15], and ciprofloxacin was used as a positive control (Table 3). The MIC of compound **1** was 6.25 μg/mL for *M. lutea*, methicillin-resistant *Staphylococcus aureus*, and *Streptococcus faecalis*; that of compound **2** was 6.25 μg/mL for methicillin-resistant *Staphylococcus aureus*; that of compound **3** was 12.5 μg/mL for methicillin-resistant *Staphylococcus aureus*; that of compound **4** was 6.25 μg/mL for *K. pneumoniae*; that of compound **5** was 12.5 μg/mL for *M. lutea*, methicillin-resistant *Staphylococcus aureus*, and *Streptococcus faecalis*; and that of compound **7** was 12.5 μg/mL for *Streptococcus faecalis*.

The cytotoxic activities of compounds **1**–**12** against SF-268, MCF-7, HepG-2, and A549 cell lines in vitro were evaluated with the SRB method [16], and adriamycin was used as a positive control (Table 4). Compounds **2** and **5** displayed weak cytotoxic activity against the SF-268, MCF-7, HepG-2, and A549 cell lines, and compound 8 displayed moderate cytotoxic activity against the SF-268, MCF-7, and A549 cell lines.

Compound **2** exhibited weak cytotoxic activity to against cancer cells, and its IC_50_ was 65.64–107.31 μM. Compound **5** showed cytotoxic activity at 54.78–58.54 μM. Compound **8** exhibited cytotoxic activity against the SF-268, MCF-7, and A549 cell lines with IC_50_ values of 12.75 ± 1.43, 9.29 ± 0.80, and 20.11 ± 2.31 μM, respectively. Comparing the cytotoxic activity of compounds **2** and **5**, **5** was slightly stronger than **2**, indicating that the hydroxylation at methyl (C-18) of **5** is an advantage in terms of cytotoxic activity. Regarding compounds **7** and **8**, **8** was significantly stronger than **7**, which did not show significant activity under 100 μM. This could indicate that prenylation in aromatic rings greatly contributes to cytotoxic activity, but the effect of double bond reduction on activity is unknown.

## 3. Materials and Methods

### 3.1. General Experimental Procedures

1D and 2D NMR spectra were recorded on an AVANCE III HD 700 (Temperature 298.0 K, Bruker, Billerica, MA, USA). Optical rotations were measured with an MCP 500 automatic polarimeter (Anton Paar, Graz, Austria) with CH_3_OH as the solvent. IR spectra were measured on an IR Affinity-1 spectrometer (Shimadzu, Kyoto, Japan). UV spectra were recorded on a UV-2600 spectrometer (Shimadzu, Tokyo, Japan). Circular dichroism spectra were measured by Chirascan circular dichroism spectrometer with the same concentration of UV measurement (Pathlength 10 mm, Applied Photophysics, Surrey, UK). HRESIMS spectra data were recorded on a MaXis quadrupole-time-of-flight mass spectrometer. Thin layer chromatography (TLC) was performed on plates precoated with silica gel GF254 (10–40 μm). Column chromatography (CC) was performed over silica gel (100–200 mesh and 200–300 mesh) (Qingdao Marine Chemical Factory, Qingdao, China) and ODS (50 μm, YMC, Kyoto, Japan). High-performance liquid chromatography was performed on an Agilent 1260 HPLC equipped with a DAD detector using an ODS column (YMC-pack ODS-A, 250 × 10 mm, 5 μm, 3 mL/min). All solvents used in CC and HPLC were of analytical grade (Tianjin Damao Chemical Plant, Tianjin, China) and chromatographic grade (Oceanpak, Goteborg, Sweden), respectively.

### 3.2. Fungal Material

The fungal strain used in this investigation was isolated from gorgonian coral collected in the Mischief Reef of the South China Sea. It was identified as *Aspergillus hiratsukae* SCSIO 7S2001, according to a molecular biological protocol by DNA amplification and sequencing of the ITS region (deposited in GenBank, accession no. MN347034). A voucher strain (SCSIO 7S2001) was deposited in the RNAM Center for Marine Microbiology, South China Sea Institute of Oceanology, Chinese Academy of Sciences.

### 3.3. Fermentation and Extraction

The fungal strain *Aspergillus hiratsukae* SCSIO 7S2001 was cultured on PDA plates (potatoes 200.0 g, glucose 20.0 g, agar 15.0 g and sea salt 30.0 g in 1.0 L H_2_O) at 28 °C for 7 days. The seed medium (potatoes 200.0 g, glucose 20.0 g and sea salt 30.0 g in 1.0 L H_2_O) was inoculated with strain SCSIO 7S2001 and incubated at 28.0 °C for 3 days on a rotating shaker (180 rpm). Then, a large-scale fermentation of fungal SCSIO 7S2001 was incubated for 30 days at 28 °C in 245 conical flasks (each flask contained 80.0 g rice and 100.0 mL H_2_O with 3‰ salinity) with solid rice medium. The whole fermented cultures were soaked in CH_3_OH/H_2_O (*v*/*v*, 7:3) overnight and filtered through cheesecloth to obtain the filtrate, and the extraction was repeated three times. The extract was evaporated under reduced pressure to evaporate the filtrate until the solvent could not be distilled out or the solvent was distilled out slowly to obtain an aqueous solution. We added twice the amount of ethyl acetate for extraction to the aqueous solution to obtain an organic phase (EtOAc-H_2_O, 2:1), and repeated the extraction 3 times. Both organic phases were combined and concentrated under reduced pressure to give the whole crude extract (138 g).

### 3.4. Isolation and Purification

The extract was subjected to silica gel vacuum liquid chromatography (VLC) eluting with a gradient of CH_3_OH-CH_2_Cl_2_ (0:1–1:0) to separate into four fractions. Fr.2 (5.8 g) was separated by a reverse-phase ODS silica gel column (CH_3_OH-H_2_O, 3:7–10:0) to obtain 10 fractions (Fr.2.1-2.10). Then, Fr.2.3 (75.8 mg) was purified by HPLC (CH_3_CN-H_2_O, 40:60) to yield **12** (42.9 mg, *t*_R_ = 8.1 min). Fr.2.7 (157.6 mg) was purified by HPLC (CH_3_CN-H_2_O, 50:50) to yield Fr.2.7.1 (6.6 mg, *t*_R_ = 26.9 min), F2.7.2 (7.8 mg, *t*_R_ = 16.2 min), and **4** (6.6 mg, *t*_R_ = 22.2 min). Fr.2.7.1 was recrystallized (EtOAc–N-hexane, 1:1) to yield **1** (5.8 mg). Fr.2.7.2 was recrystallized (EtOAc–N-hexane, 1:1) to yield **2** (5.8 mg). Fr.2.8 (139.2 mg) was purified by HPLC (CH_3_CN-H_2_O, 60:40) to yield **3** (26.4 mg, *t*_R_ = 21.1 min), **5** (11.9 mg, *t*_R_ = 15.5 min), and **6** (6.1 mg, *t*_R_ = 17.9 min). Fr.2.10 (661.8 mg) was divided into two fractions by Sephadex LH-20 (CH_3_OH-CH_2_Cl_2_ 1:1). Fr.2.10.1 was further purified by HPLC (CH_3_OH-H_2_O, 70:30) to yield **7** (5.7 mg, *t*_R_ = 9.1 min). Fr.2.10.2 was further purified by HPLC (CH_3_OH-H_2_O, 90:10) to yield **8** (5.6 mg, *t*_R_ = 12.1 min). Fr.3 was separated by a reverse-phase ODS silica gel column (CH_3_OH-H_2_O, 3:7–10:0) to obtain six fractions. Fr.3.3 was subjected to Sephadex LH-20 (CH_3_OH-CH_2_Cl_2_, 1:1) to obtain three fractions. Fr.3.3.1 was purified by HPLC (CH_3_CN-H_2_O, 30:70) to yield **9** (1.0 mg, *t*_R_ = 17.3 min). Fr.3.3.2 was purified by HPLC (CH_3_CN-H_2_O, 20:80) to yield **10** (3.7 mg, *t*_R_ = 27.0 min). Fr.3.3.3 was purified by HPLC (CH_3_CN-H_2_O, 32:68) to yield **11** (5.6 mg, *t*_R_ =13.3 min).

Chevalone H (**1**): white needle crystals. [α${]}_{D}^{25}$ = −57.4° (c 0.2, CH_3_OH). UV (c 0.5 mmol/L, CH_3_OH): λ_max_ (log *ε*) 205(4.39), 286(3.72) nm; IR (film) ν_max_ 3524, 2932, 2872, 1697, 1576, 1456, 1246, 1036 cm^−1^; HR-ESIMS at *m*/*z* 511.2673 [M + Na]^+^, calcd for C_28_H_41_O_8_, 511.2666. ^1^H NMR (CDCl_3_, 700 MHz) and ^13^C NMR (CDCl_3_, 176 MHz) see Table 1.

Chevalone I (**2**): white needle crystals. [α${]}_{D}^{25}$ = −74.8° (c 0.2, CH_3_OH); UV (c 0.5 mmol/L, CH_3_OH): λ_max_ (log *ε*) 206(4.26), 286(3.73) nm; IR (film) ν_max_ 3429, 2936, 2864, 1697, 1653, 1578, 1449, 1387, 1237, 1031, 997 cm^−1^; HRESIMS at *m*/*z* 473.2907 [M + H]^+^, calcd for C_28_H_41_O_6_, 473.2898. ^1^H NMR (CDCl_3_, 700 MHz) and ^13^C NMR (CDCl_3_, 176 MHz) see Table 1.

Chevalone J (**3**): white solid. [α${]}_{D}^{25}$ = −6.6° (c 0.2, CH_3_OH). UV (c 0.5 mmol/L, CH_3_OH): λ_max_ (log *ε*) 203(4.22), 286(3.56) nm. IR (film) ν_max_ 3366, 2936, 2857, 1697, 1653, 1541, 1456, 1024 cm^−1^. HR-ESIMS at *m*/*z* 527.2609 [M + Na]^+^, calcd for C_28_H_40_NaO_8_, 527.2615. ^1^H NMR (CDCl_3_, 700 MHz) and ^13^C NMR (CDCl_3_, 176 MHz) see Table 1.

Chevalone K (**4**): white solid. [α${]}_{D}^{25}$ = −21.6° (c 0.2, CH_3_OH). UV (c 0.5 mmol/L, CH_3_OH): λ_max_ (log *ε*) 205(4.32), 286(3.71) nm; IR (film) ν_max_ 3363, 2941, 1645, 1516, 1447, 1105, cm^−1^; HR-ESIMS *m*/*z* 489.2857 [M + H]^+^, calcd for C_28_H_41_O_7_, 489.2847. ^1^H NMR (CD_3_OD, 700 MHz) and ^13^C NMR (CD_3_OD, 176 MHz) see Table 2.

Chevalone L (**5**): white solid. [α${]}_{D}^{25}$ = −35.6° (c 0.2, CH_3_OH). UV (c 0.5 mmol/L, CH_3_OH): λ_max_ (log *ε*) 205(4.32), 286(3.71) nm; IR (film) ν_max_ 3362, 2928, 2851, 1697, 1653, 1576, 1506, 1238, 1026 cm^−1^; HR-ESIMS at *m*/*z* 489.2839 [M + H]^+^, calcd for C_28_H_41_O_7_, 489.2847. ^1^H NMR (CDCl_3_, 700 MHz) and ^13^C NMR (CDCl_3_, 176 MHz) see Table 2.

Chevalone M (**6**): white solid. [α${]}_{D}^{25}$ = −6.8° (c 0.2, CH_3_OH). UV (c 0.5 mmol/L, CH_3_OH): λ_max_ (log *ε*) 205(4.32), 286(3.71) nm. IR (film) ν_max_ 3420, 2934, 1695, 1587, 1236, 1026; HR-ESIMS *m*/*z* 473.2892 [M + H]^+^, calcd for C_28_H_41_O_6_, 473.2898. ^1^H NMR (CDCl_3_, 700 MHz) and ^13^C NMR (CDCl_3_, 176 MHz) see Table 2.

### 3.5. Crystal Structure Analysis

Crystallographic data for the compounds chevalone H (**1**) and chevalone I (**2**) were collected on a Rigaku XtaLAB AFC12 single-crystal diffractometer using Cu-Kα radiation (λ = 0.71073 A) at 298(2) K. The structures of **1** and **2** were solved by direct methods (SHELXS97), expanded using difference Fourier techniques, and refined by full-matrix least-squares calculation [17]. The nonhydrogen atoms were refined anisotropically, and hydrogen atoms were fixed at calculated positions. Crystallographic data of compounds **1** and **2** have been deposited at the Cambridge Crystallographic Data Center under the reference numbers CCDC 1,989,730 and 1,989,731. Copies of the data could be obtained free of charge from the CCDC at www.ccdc.cam.ac.uk (accessed on 11 March 2020).

Crystal data for **1**: C_28_H_40_O_7_, M = 488.27 g/mol, orthorhombic, space group P212121, *a* = 10.74060(10) Å, *b* = 15.17330(10) Å, *c* = 36.9556(4) Å, *V* = 6022.67(10) Å^3^, *Z* = 4, *T* = 105 K, μ (Cu-Kα) = 0.750 mm^−1^, *D_calc_* = 1.271 g/cm^3^, 32,806 reflections measured (7.538° ≤ *θ* ≤ 148.882°), 11,913 unique (*R_int_* = 0.0284, *R_sigma_* = 0.0310) which were used in all calculations. The final *R_1_* was 0.0354 (I > 2*σ*(I)), and *wR_2_* was 0.0906 (all data). The goodness of fit on *F*_2_ was 1.053. Flack parameter = 0.03(4).

Crystal data for **2**: C_28_H_40_O_6_, M =472.29 g/mol, orthorhombic, space group P212121, *a* = 5.99790(10) Å, *b* = 30.7241(6) Å, *c* = 13.2008(2) Å, *V*= 2432.64(7) Å^3^, *Z* = 4, *T* = 105(8) K, μ(Cu-Kα) = 0.718 mm^−1^, *D_calc_* = 1.290 g/cm^3^, 11,762 reflections measured (7.288° ≤ *θ* ≤ 148.656°), 4812 unique (*R_int_* = 0.0348, *R_sigma_* = 0.0405) which were used in all calculations. The final *R_1_* was 0.0405 (I > 2*σ*(I)), and *wR_2_* was 0.1063 (all data). The goodness of fit on *F_2_* was 1.099. Flack parameter = −0.02(10).

### 3.6. Antibacterial Activity

The antibacterial activities were evaluated with the broth dilution assay [15]. Four bacterial strains (*Klebsiella pneumoniae* ATCC 13883, *Streptococcus faecalis* ATCC 29212, methicillin-resistant *Staphylococcus aureus* 01, and *Micrococcus lutea* 01) were used, and ciprofloxacin was used as a positive control.

### 3.7. Cytotoxicity Activity

Cytotoxicity against SF268 (human glioblastoma carcinoma), MCF-7 (breast cancer), HepG-2 (liver cancer) and A549 (lung cancer) cell lines was assayed by the sulforhodamine (SRB) method [16,18]. Adriamycin was used as a positive control possessing potent cytotoxic activity. IC_50_ values were calculated with SPSS software using a nonlinear curve-fitting method. The cells of SF-268, MCF-7, HepG-2, and A549 were purchased from Stem Cell Bank, Chinese Academy of Sciences.

## 4. Conclusions

In this study, bioactive secondary metabolites were isolated from the gorgonian coral-derived fungus *Aspergillus hiratsukae* SCSIO 7S2001: six new α-pyrone meroterpenoid chevalones H–M (**1**–**6**), together with six known compounds (**7**–**12**). The absolute configurations of the new compounds were deduced by combining the NOE spectrum, X-ray single crystal diffraction, and ECD spectra. Compounds **1**–**6** were a series of chevalone derivatives substituted by hydroxy groups based on the meroterpenoid skeleton. Furthermore, the presence of some impurity peaks in the spectral tests of compounds **3** and **6** was due to their low yields, which may be unnecessarily lost by further purification, and this did not affect their qualitative analysis.

Antimicrobial resistance is a global health and development threat and has become one of the most important public health threats facing humanity in the 21st century [19]. In this study, compounds **1**–**5** and **7** with bacterial inhibitory potential were screened. In terms of antitumor cell activity, compound **8** exhibited antitumor activity in different cancer cells.

Compound **11** has rarely been reported in marine fungi, and previous studies have shown that trans-cinnamic acid may be an environmentally friendly alternative therapeutic agent for bacterial infections in the aquaculture industry [20]. Compound **12** has been isolated from marine fungi but has not yet been found to have biological activity [21,22].

## Figures and Tables

**Table 3:** The antibacterial activities of compounds **1**–**12**.

Compounds	MIC (μg/mL)			
	*Micrococcus lutea*	*Klebsiella pneumoniae*	Methicillin-Resistant *Staphylococcus aureus*	*Streptococcus faecalis*
**1**	6.25	50	6.25	6.25
**2**	25	>100	6.25	25
**3**	25	25	12.5	>100
**4**	>100	6.25	25	50
**5**	12.5	>100	12.5	12.5
**6**	>100	>100	>100	>100
**7**	>100	50	>100	12.5
**8**	>100	>100	>100	>100
**9**	>100	>100	>100	>100
**10**	>100	>100	>100	>100
**11**	>100	>100	>100	>100
**12**	>100	>100	>100	>100
Ciprofloxacin	0.25	0.25	0.50	0.50

**Table 4:** Cytotoxic activity of compounds (**1**–**12**) against tumor cells.

Compounds	IC_50_ (μM)			
	SF-268	MCF-7	HepG-2	A549
**1**	>128	>128	>128	>128
**2**	65.64 ± 0.53	91.69 ± 6.59	107.31 ± 9.83	84.54 ± 16.23
**3**	>128	>128	>128	>128
**4**	>128	>128	>128	>128
**5**	54.78 ± 3.18	56.28 ± 2.05	58.54 ± 1.52	55.33 ± 1.60
**6**	>128	>128	>128	>128
**7**	>128	>128	>128	>128
**8**	12.75 ± 1.43	9.29 ± 0.80	>128	20.11 ± 2.31
**9**	>128	>128	>128	>128
**10**	>128	>128	>128	>128
**11**	>128	>128	>128	>128
**12**	>128	>128	>128	>128
Adriamycin	1.94 ± 0.01	2.00 ± 0.04	2.16 ± 0.05	2.16 ± 0.07

## Data Availability

Data are contained within the article or Supplementary Material.

## Supplementary Materials

The following are available online at https://www.mdpi.com/article/10.3390/md20010071/s1, Figures S1–S53: ^1^H NMR, ^13^C NMR, DEPT, HSQC, HMBC, COSY, IR, and HR-ESIMS spectra of compounds **1**–**6**. Figures S54–S72: ^1^H NMR, ^13^C NMR spectra of compounds **7**–**12**. Figures S73–S76: Experimental ECD spectra of compounds **1**–**6**.
