# Supplementary material for: Chevalones H–M: Six New α-Pyrone Meroterpenoids from the Gorgonian Coral-Derived Fungus Aspergillus hiratsukae SCSIO 7S2001
Source: Mar Drugs. 2022 Jan 14;20(1):71. doi: 10.3390/md20010071 (PMC8781156; doi:10.3390/md20010071)
Supplement: Supplementary file 1 [file marinedrugs-20-00071-s001.zip › marinedrugs-1527414-supplementary.pdf]

Chevalones H-M: Six New α-Pyrone Meroterpenoids from the Gorgonian Coral-derived Fungus *Aspergillus hiratsukae* SCSIO 7S2001

Xia-Yu Chen [1, 2], Qi Zeng [1, 2], Yu-Chan Chen [3], Wei-Mao Zhong [1], Yao Xiang [1, 2], Jun-Feng Wang [1], Xue-Feng Shi [1], Si Zhang [1], and Fa-Zuo Wang [1, *]

[1] CAS Key Laboratory of Tropical Marine Bio-resources and Ecology, Southern Marine Science and Engineering Guangdong Laboratory (Guangzhou), Guangdong Key Laboratory of Marine Materia Medica, RNAM Center for Marine Microbiology, South China Sea Institute of Oceanology, Chinese Academy of Sciences, 164 West Xingang Road, Guangzhou 510301, China; chenxiayu17@mails.ucas.ac.cn; 18489875310@163.com; 792193347@qq.com; xy920412@sina.cn; shixuefeng@scsio.ac.cn; shixuefeng@scsio.ac.cn; zhsimd@scsio.ac.cn;

[2] University of Chinese Academy of Sciences, 19 Yuquan Road, Beijing 100049, China;

[3] State Key Laboratory of Applied Microbiology Southern China, Guangdong Provincial Key Laboratory of Microbial Culture Collection and Application, Guangdong Open Laboratory of Applied Microbiology, Guangdong Institute of Microbiology, 100 Central Xianlie Road, Guangzhou 510070, China; chenyc@gdim.cn

* Correspondence: wangfazuo@scsio.ac.cn.

Supplementary Information

Figure S1. The ^1H NMR spectrum of compound 1 in $CDCl_3$. 5

Figure S2. The ^1H NMR spectrum (0-3 ppm) of compound 1 in $CDCl_3$......6

Figure S3. The ^{13}C NMR spectrum of compound 1 in $CDCl_3$......7

Figure S4. The ^1H-^1H COSY spectrum of compound 1 in $CDCl_3$......8

Figure S5. The HSQC spectrum of compound 1 in $CDCl_3$. 9

Figure S6. The HMBC spectrum of compound 1 in $CDCl_3$. 10

Figure S7. The NOESY spectrum of compound 1 in $CDCl_3$. 11

Figure S8. The HRESIMS spectra of compound 1. 12

Figure S9. The IR spectrum of compound 1......12

Figure S10. The ^1H NMR spectrum of compound 2 in $CDCl_3$......13

Figure S11. The ^1H NMR spectrum (0-3 ppm) of compound 2 in $CDCl_3$. 14

Figure S12. The ^{13}C NMR spectrum of compound 2 in $CDCl_3$......15

Figure S13. The HSQC spectrum of compound 2 in $CDCl_3$. 16

Figure S14. The HMBC spectrum of compound 2 in $CDCl_3$. 17

Figure S15. The NOESY spectrum of compound 2 in $CDCl_3$......18

Figure S16. The HRESIMS spectrum of compound 2......19

Figure S17. The IR spectrum of compound 2......19

Figure S18. The ^1H spectrum of compound 3 in $CDCl_3$. 20

Figure S19. The ^1H spectrum (0-3 ppm) of compound 3 in $CDCl_3$......21

Figure S20. The ^{13}C spectrum of compound 3 in $CDCl_3$. 22

Figure S21. The ^1H-^1H COSY spectrum of compound 3 in $CDCl_3$......23

Figure S22. The HSQC spectrum of compound 3 in $CDCl_3$. 24

Figure S23. The HMBC spectrum of compound 3 in $CDCl_3$. 25

Figure S24. The NOESY spectrum of compound 3 in $CDCl_3$......26

Figure S25. The HRESIMS spectra of compound 3. 27

Figure S26. The IR spectrum of compound 3......27

Figure S27. The ^1H NMR spectrum of compound 4 in CD_3OD......28

Figure S28. The ^1H NMR spectrum (0-3 ppm) of compound 4 in CD_3OD. 29

Figure S29. The ^{13}C NMR spectrum of compound 4 in CD_3OD. 30

Figure S30. The ^1H-^1H COSY spectrum of compound 4 in CD_3OD......31

Figure S31. The HSQC spectrum of compound 4 in CD_3OD. **32**

Figure S32. The HMBC spectrum of compound 4 in CD_3OD. **33**

Figure S33. The NOESY spectrum of compound 4 in CD_3OD. **34**

Figure S34. The HRESIMS spectrum of compound 4. .... **35**

Figure S35. The IR spectrum of compound 4. .... **35**

Figure S36. The ^1H NMR spectrum of compound 5 in $CDCl_3$. **36**

Figure S37. The ^1H NMR spectrum (0-3 ppm) of compound 5 in $CDCl_3$. **37**

Figure S38. The ^{13}C NMR spectrum of compound 5 in $CDCl_3$. **38**

Figure S39. The ^1H-^1H COSY spectrum of compound 5 in $CDCl_3$. **39**

Figure S40. The HSQC spectrum of compound 5 in $CDCl_3$. **40**

Figure S41. The HMBC spectrum of compound 5 in $CDCl_3$. **41**

Figure S42. The NOESY spectrum of compound 5 in $CDCl_3$. **42**

Figure S43. The HRESIMS spectrum of compound 5. .... **43**

Figure S44. The IR spectrum of compound 5. .... **43**

Figure S45. The ^1H NMR spectrum of compound 6 in CD_3OD. **44**

Figure S46. The ^1H NMR spectrum (0-3 ppm) of compound 6 in CD_3OD. **45**

Figure S47. The ^{13}C NMR spectrum of compound 6 in CD_3OD. **46**

Figure S48. The ^1H-^1H COSY spectrum of compound 6 in CD_3OD. **47**

Figure S49. The HSQC spectrum of compound 6 in CD_3OD. **48**

Figure S50. The HMBC spectrum of compound 6 in CD_3OD. **49**

Figure S51. The NOESY spectrum of compound 6 in CD_3OD. **50**

Figure S52. The HRESIMS spectrum of compound 6. .... **51**

Figure S53. The IR spectrum of compound 6. .... **51**

Figure S54. The ^1H NMR spectrum of compound 7 in $CDCl_3$. **52**

Figure S55. The ^1H NMR spectrum (4-8 ppm) of compound 7 in $CDCl_3$. **53**

Figure S56. The ^{13}C NMR spectrum of compound 7 in $CDCl_3$. **54**

Figure S57. The ^1H NMR spectrum of compound 8 in DMSO-d_6. .... **55**

Figure S58. The ^1H NMR spectrum (3-8 ppm) of compound 8 in DMSO-d_6. .... **56**

Figure S59. The ^{13}C NMR spectrum of compound 8 in DMSO-d_6. .... **57**

Figure S60. The ^1H NMR spectrum of compound 9 in $CDCl_3$. **58**

Figure S61. The ^1H NMR spectrum (2.8-4 ppm) of compound 9 in $CDCl_3$. **59**

Figure S62. The ^1H NMR spectrum (6-8 ppm) of compound 9 in $CDCl_3$. **60**

Figure S63. The ^{13}C NMR spectrum of compound 9 in $CDCl_3$. 61

Figure S64. The ^{1}H NMR spectrum of compound 10 in $CDCl_3$. 62

Figure S65. The ^{1}H NMR spectrum (0-5 ppm) of compound 10 in $CDCl_3$. 63

Figure S66. The ^{13}C NMR spectrum of compound 10 in $CDCl_3$. 64

Figure S67. The ^{1}H NMR spectrum of compound 11 in $CDCl_3$. 65

Figure S68. The ^{1}H NMR spectrum (6-8 ppm) of compound 11 in $CDCl_3$. 66

Figure S69. The ^{13}C NMR spectrum of compound 11 in $CDCl_3$. 67

Figure S70. The ^{1}H NMR spectrum of compound 12 in $CDCl_3$. 68

Figure S71. The ^{1}H NMR spectrum (5-8 ppm) of compound 12 in $CDCl_3$. 69

Figure S72. The ^{13}C NMR spectrum of compound 12 in $CDCl_3$ 70

Figure S73. Experimental ECD spectra of compounds 1, 2 and 3. 71

Figure S74. Experimental ECD spectra of compounds 1, 2 and 4. 71

Figure S75. Experimental ECD spectra of compounds 1, 2 and 5. 72

Figure S76. Experimental ECD spectra of compounds 1, 2 and 6. 72

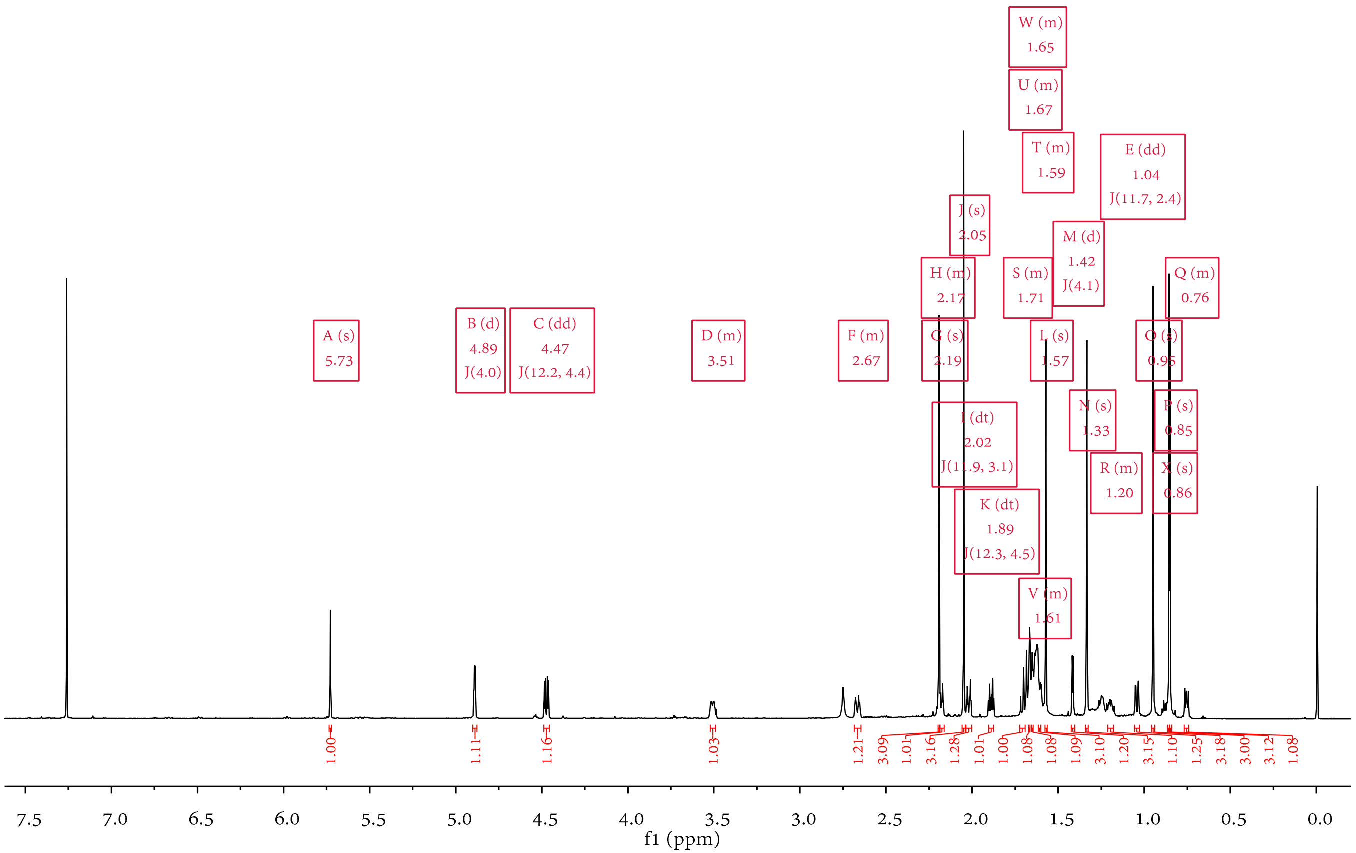

Figure S1. The ^{1}H NMR spectrum of compound 1 in $CDCl_3$.

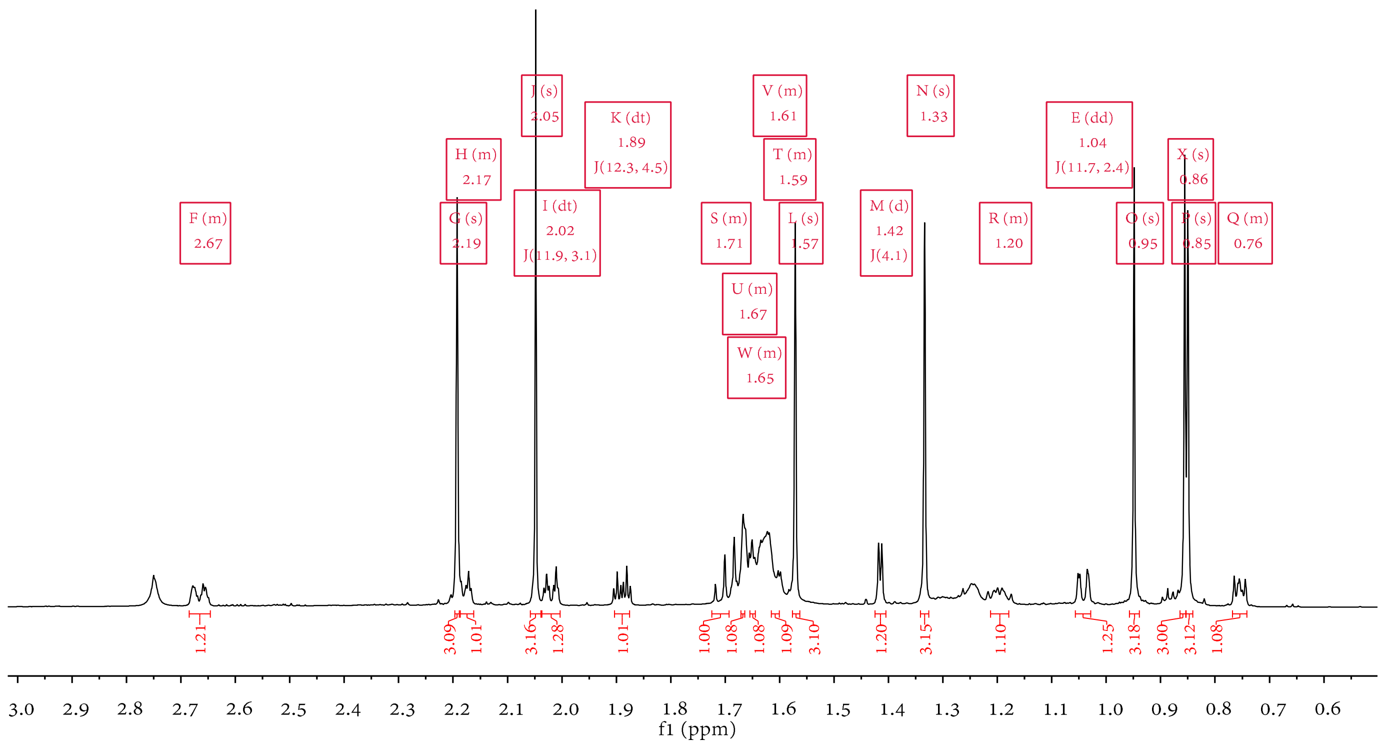

Figure S2. The ^{1}H NMR spectrum (0-3 ppm) of compound 1 in $CDCl_3$.

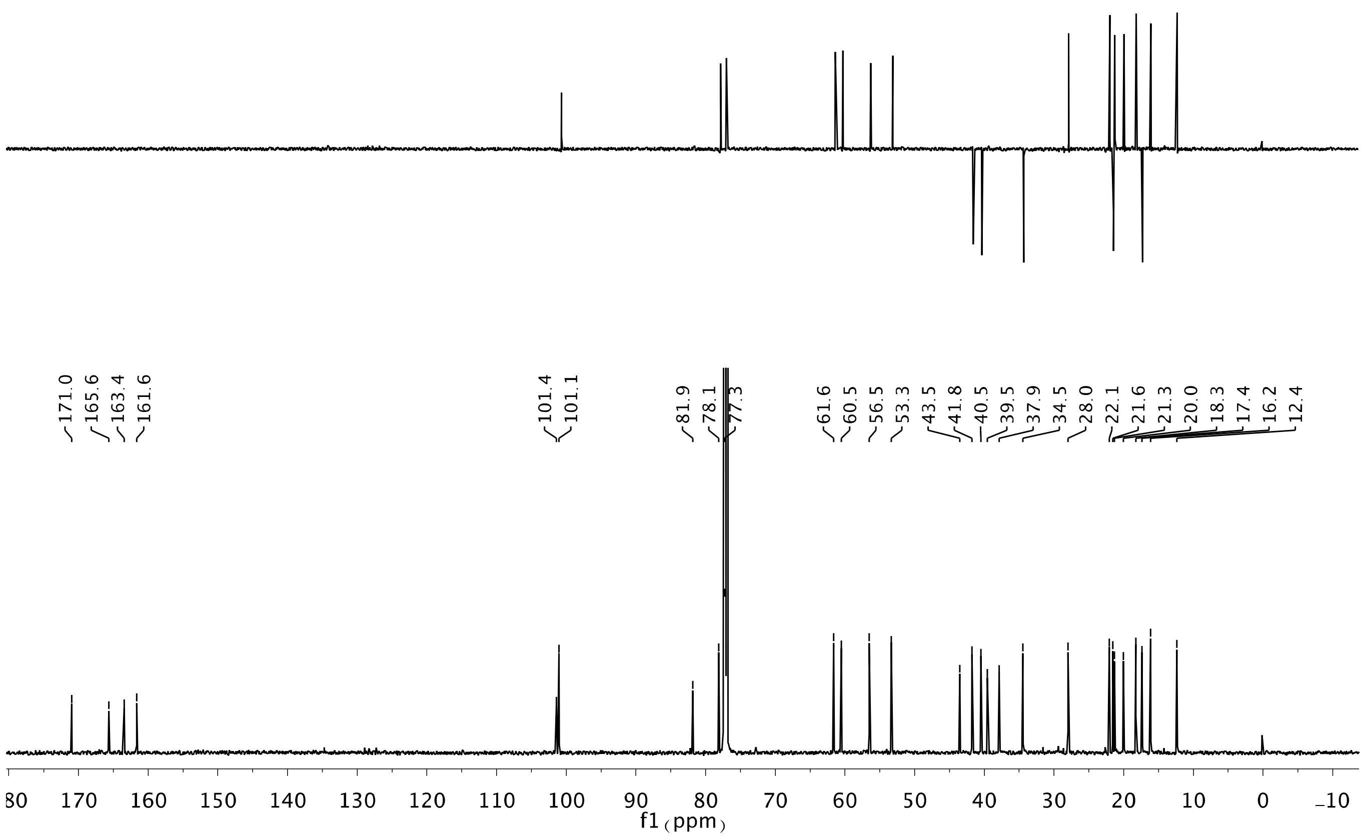

Figure S3. The ^{13}C NMR spectrum of compound 1 in $CDCl_3$.

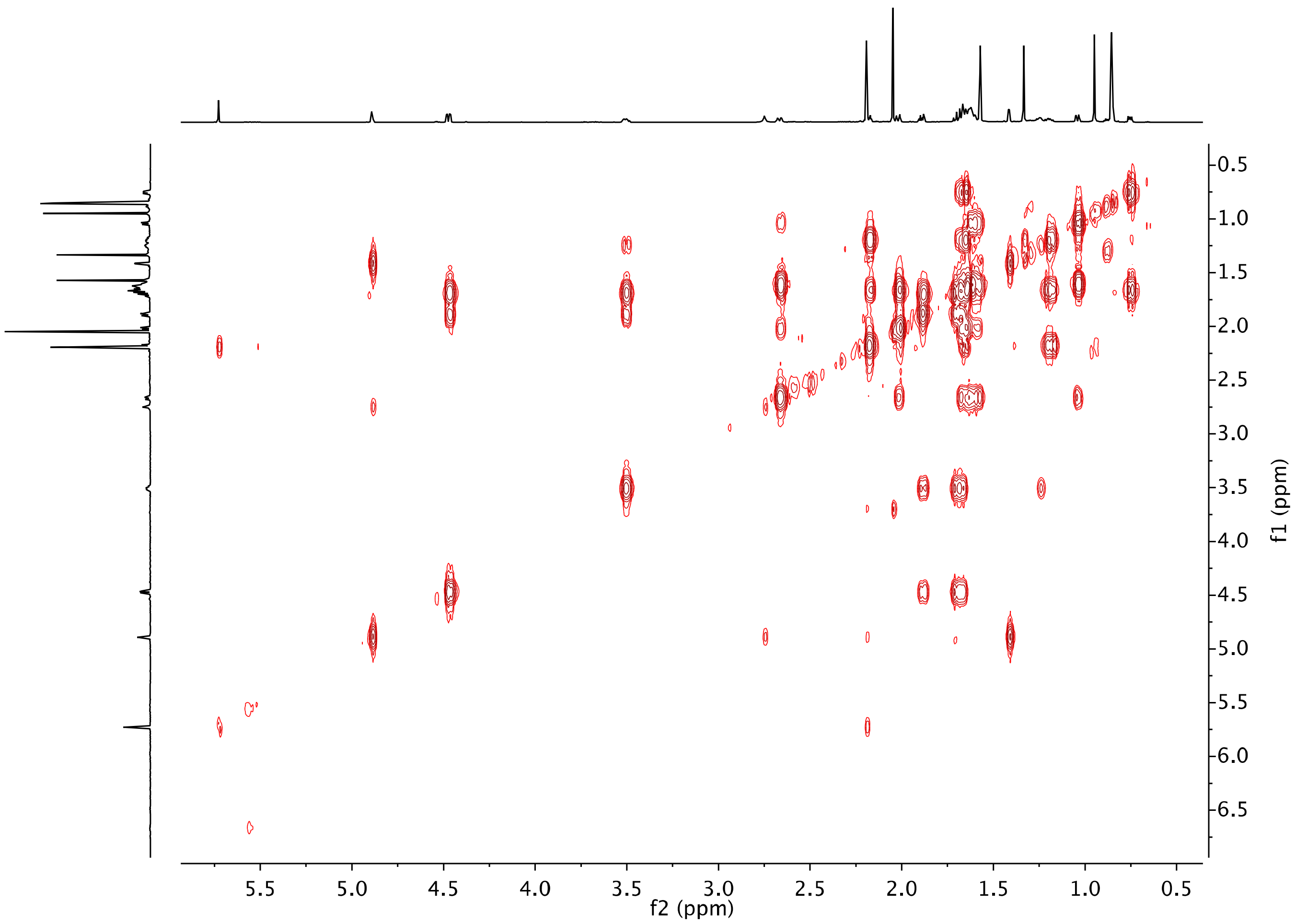

Figure S4. The ^{1}H-^{1}H COSY spectrum of compound 1 in $CDCl_3$.

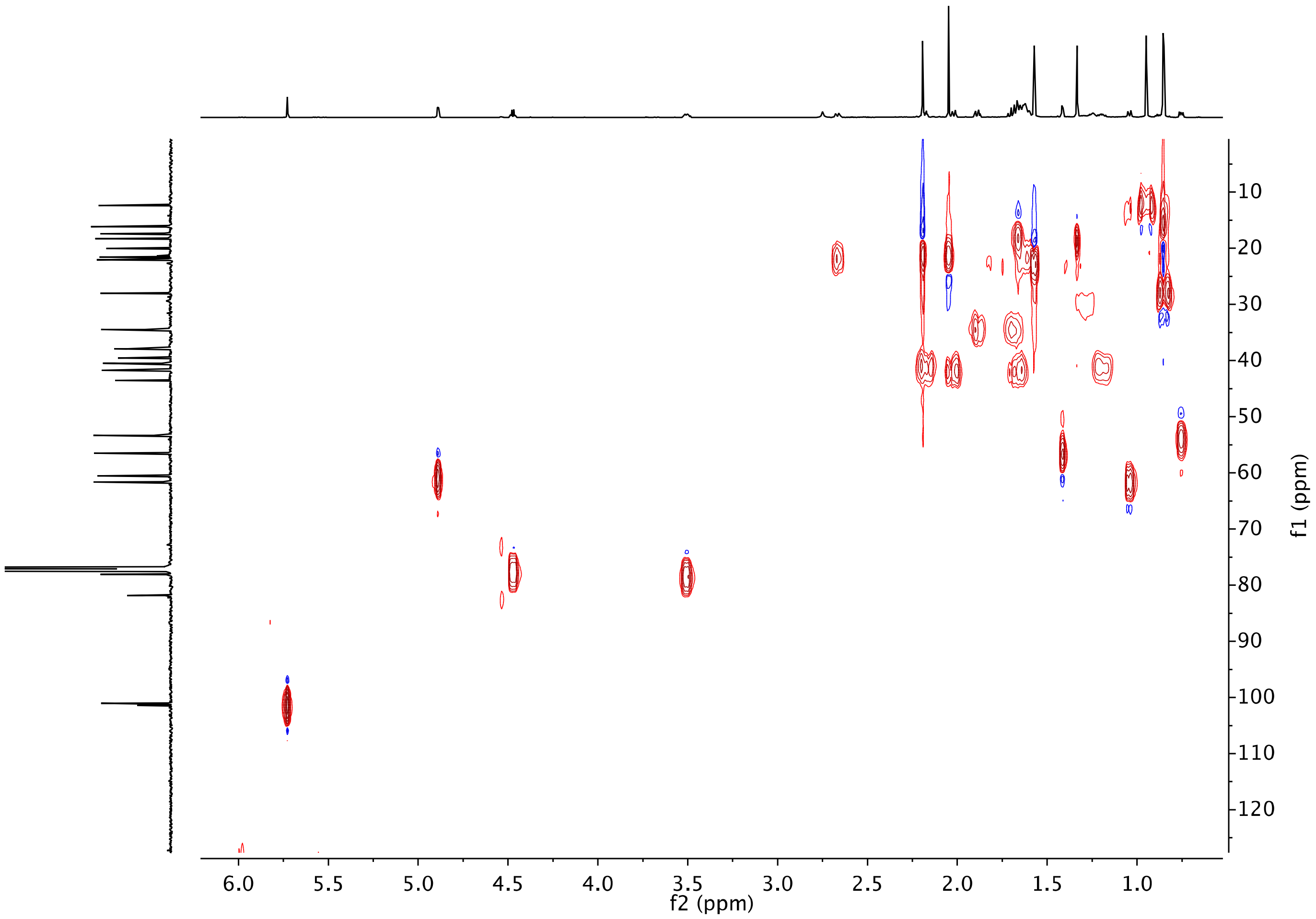

Figure S5. The HSQC spectrum of compound 1 in $CDCl_3$.

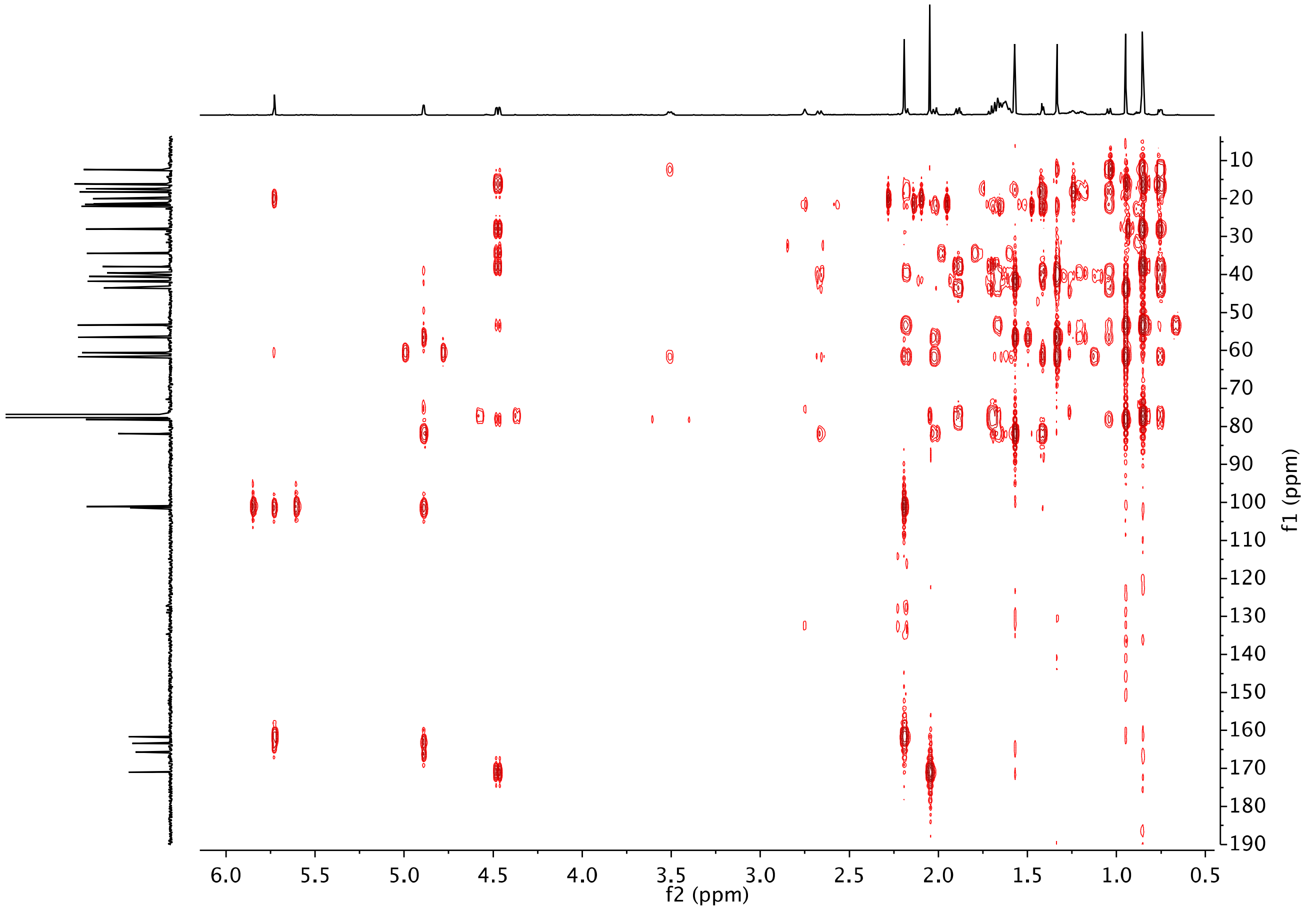

Figure S6. The HMBC spectrum of compound 1 in $CDCl_3$.

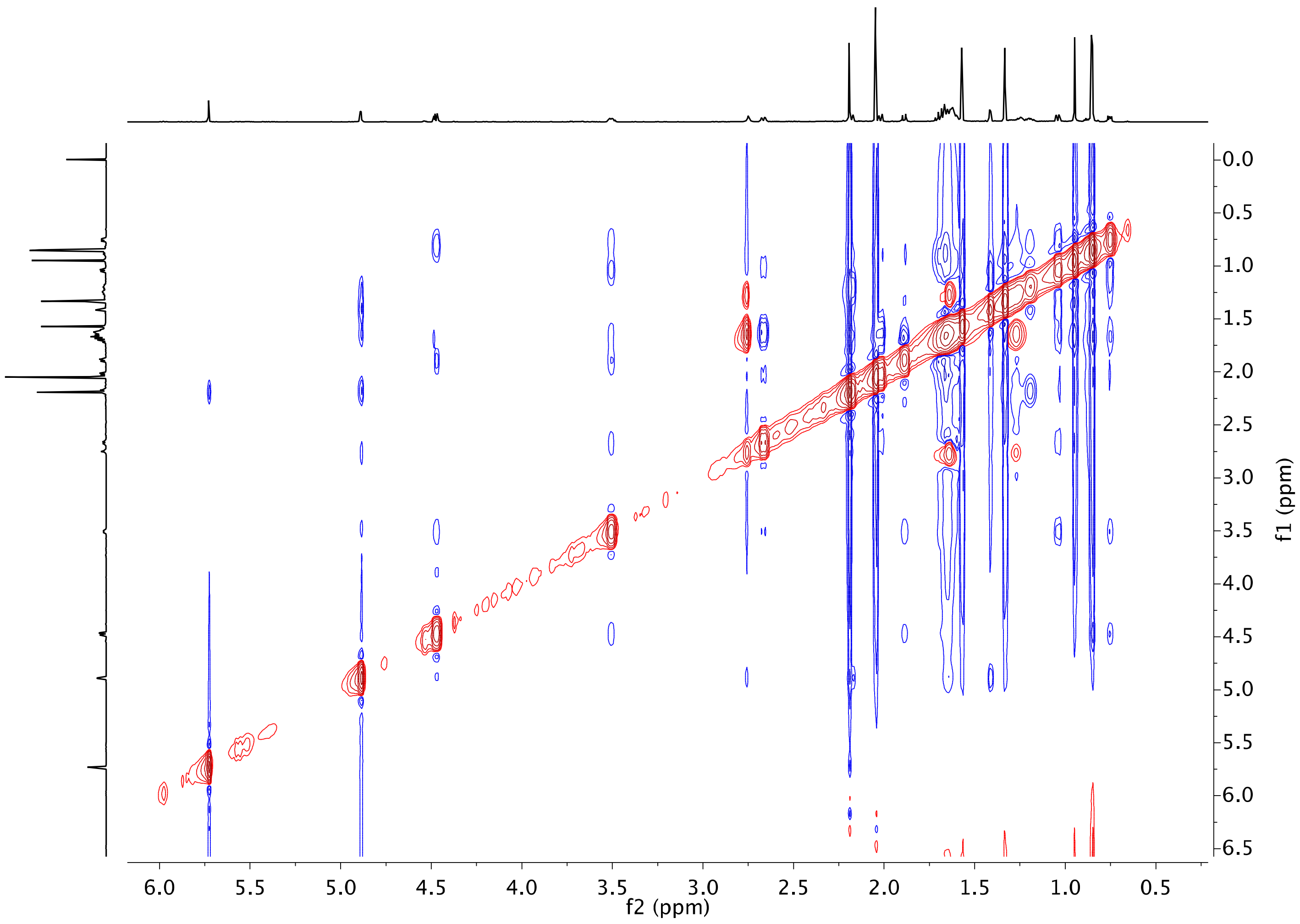

Figure S7. The NOESY spectrum of compound 1 in $CDCl_3$.

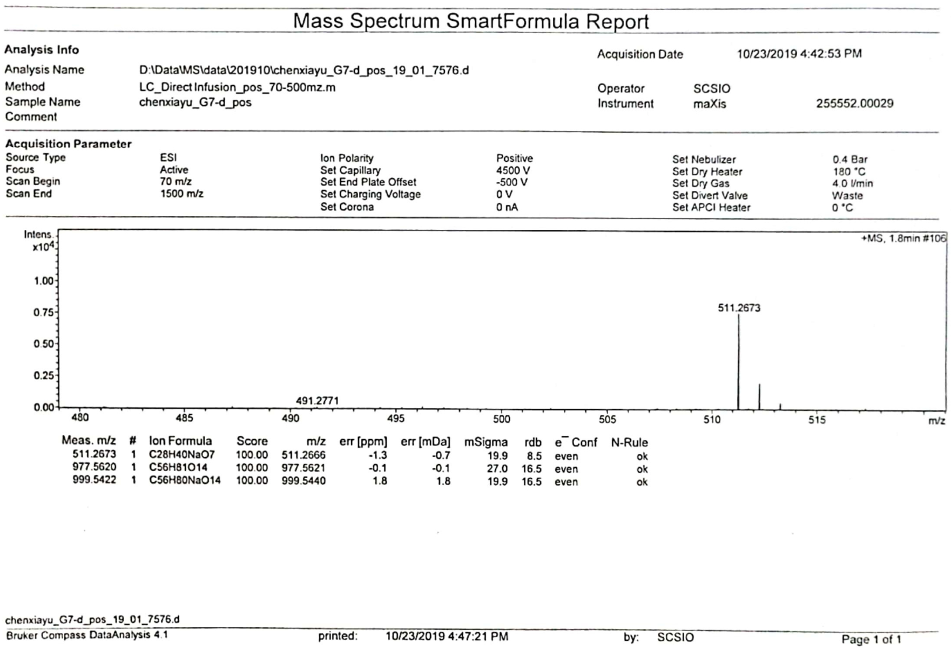

Mass Spectrum SmartFormula Report

Analysis Info

Analysis Name	D:\Data\MS\data\201910\chenxiayu_G7-d_pos_19_01_7576.d	Acquisition Date	10/23/2019 4:42:53 PM	
Method	LC_Direct Infusion_pos_70-500mz.m	Operator	SCSIO	
Sample Name	chenxiayu_G7-d_pos	Instrument	maXis	255552.00029
Comment				

Acquisition Parameter

Source Type	ESI	Ion Polarity	Positive	Set Nebulizer	0.4 Bar
Focus	Active	Set Capillary	4500 V	Set Dry Heater	180 °C
Scan Begin	70 m/z	Set End Plate Offset	-500 V	Set Dry Gas	4.0 l/min
Scan End	1500 m/z	Set Charging Voltage	0 V	Set Divert Valve	Waste
		Set Corona	0 nA	Set APCI Heater	0 °C

Meas. m/z	#	Ion Formula	Score	m/z	err [ppm]	err [mDa]	mSigma	rdb	e⁻ Conf	N-Rule
511.2673	1	C28H40NaO7	100.00	511.2666	-1.3	-0.7	19.9	8.5	even	ok
977.5620	1	C56H81O14	100.00	977.5621	-0.1	-0.1	27.0	16.5	even	ok
999.5422	1	C56H80NaO14	100.00	999.5440	1.8	1.8	19.9	16.5	even	ok

chenxiayu_G7-d_pos_19_01_7576.d

Bruker Compass DataAnalysis 4.1 printed: 10/23/2019 4:47:21 PM by: SCSIO Page 1 of 1

Figure S8. The HRESIMS spectra of compound 1.

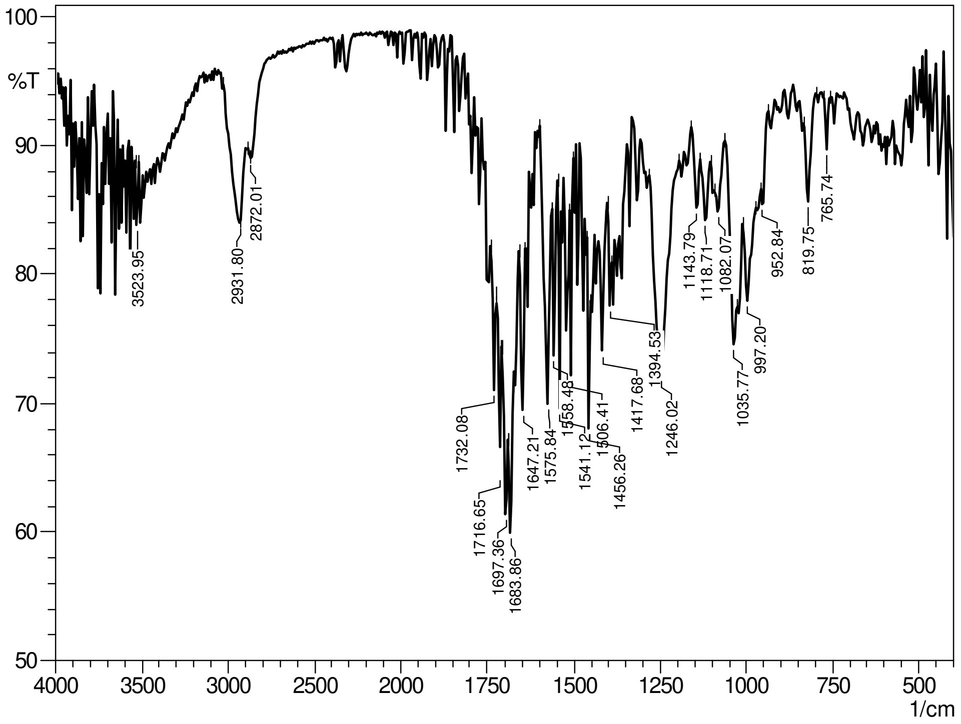

Figure S9. The IR spectrum of compound 1.

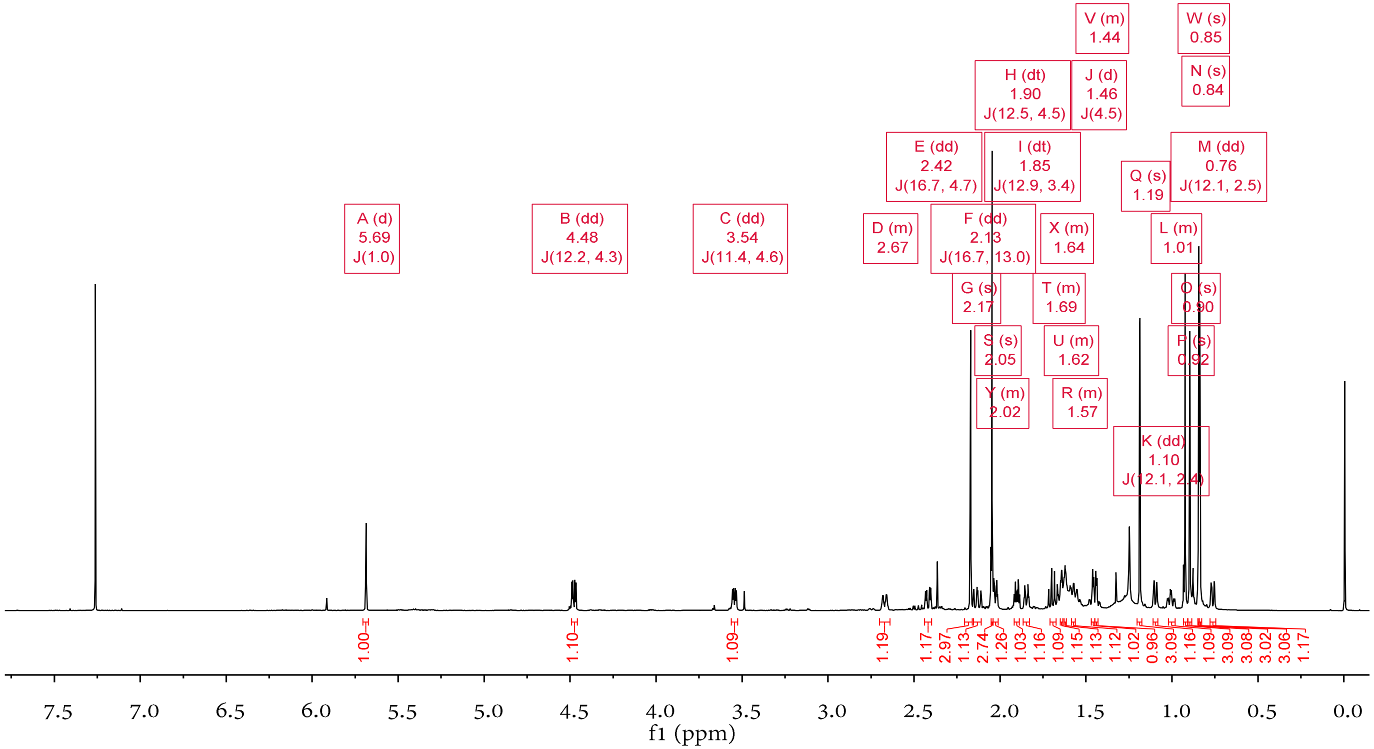

Figure S10. The ^{1}H NMR spectrum of compound 2 in $CDCl_3$.

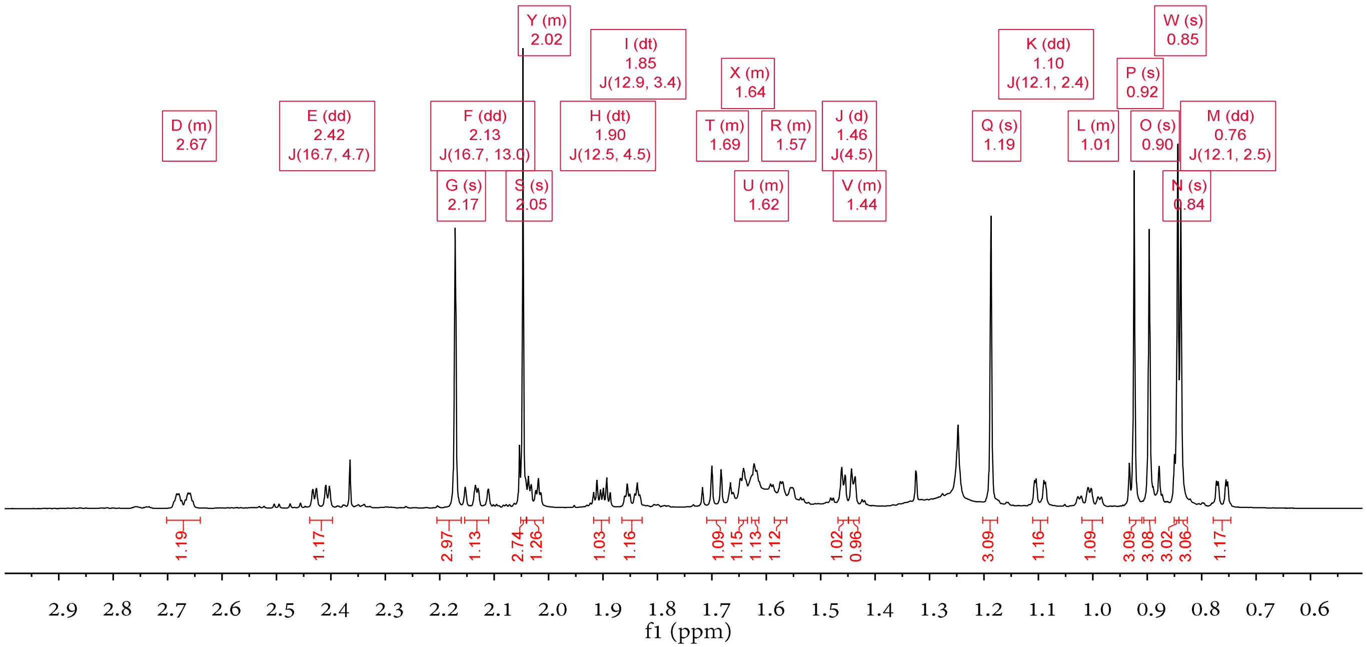

Figure S11. The ^{1}H NMR spectrum (0-3 ppm) of compound 2 in $CDCl_3$.

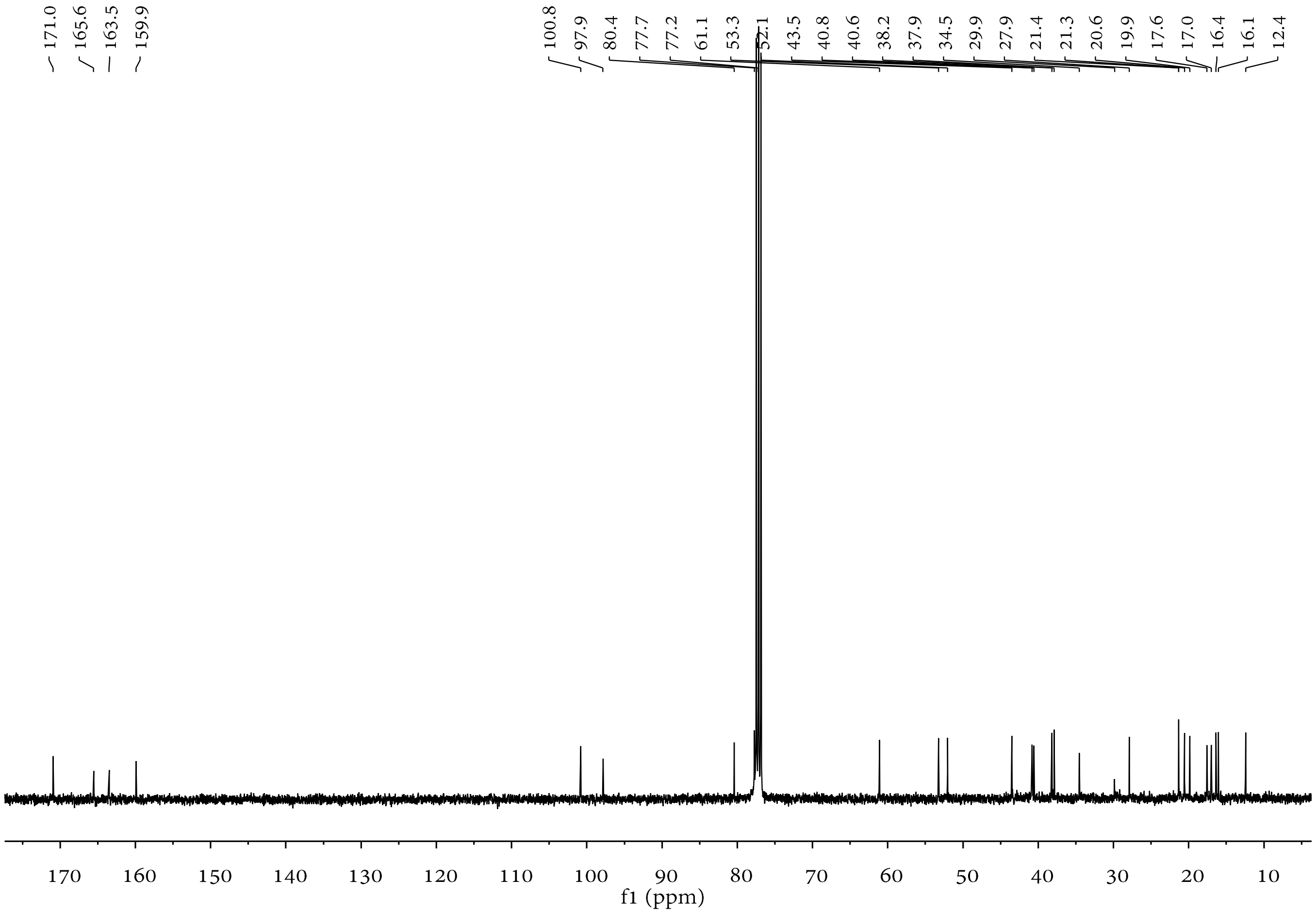

Figure S12. The ^{13}C NMR spectrum of compound 2 in $CDCl_3$.

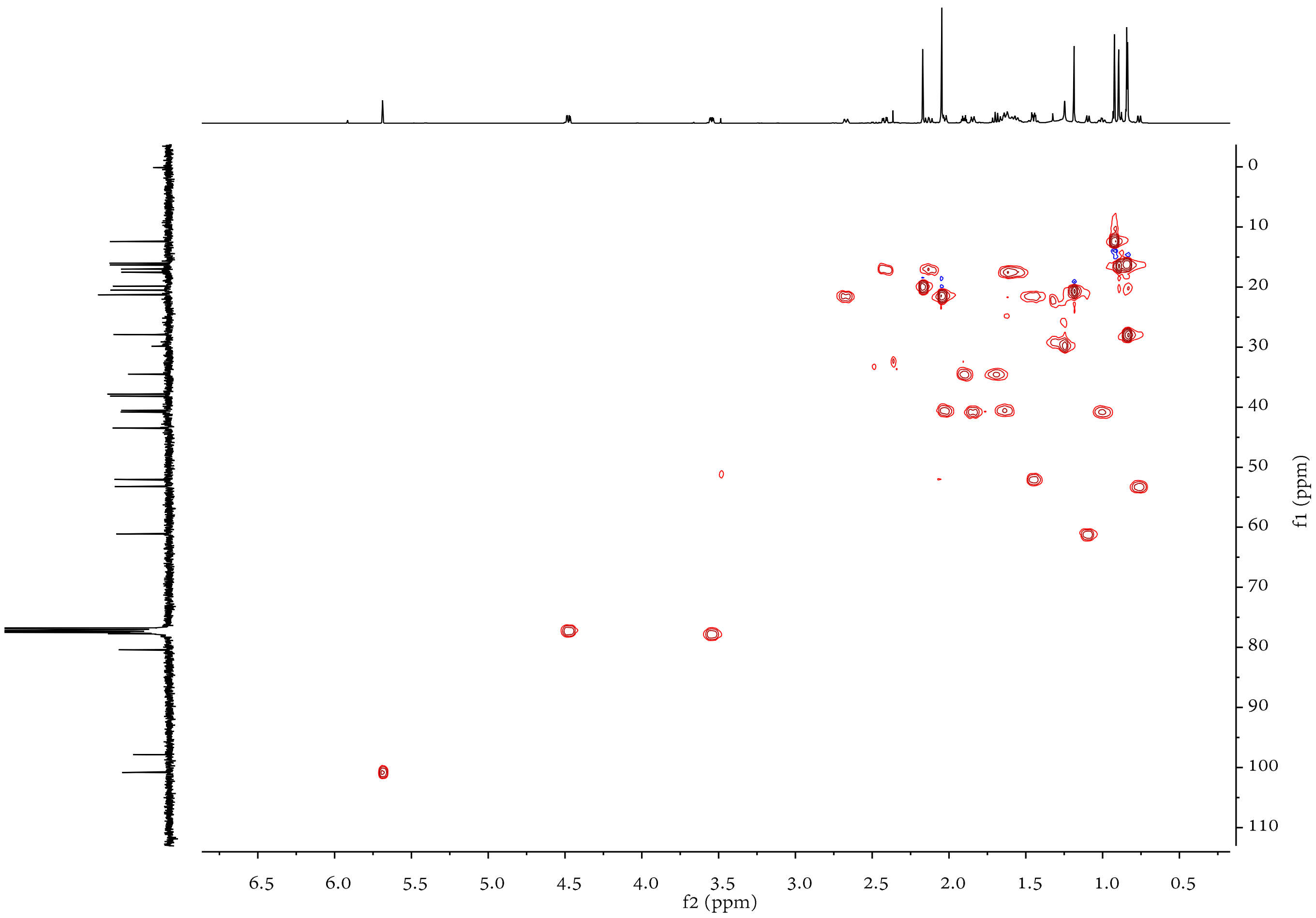

Figure S13. The HSQC spectrum of compound 2 in $CDCl_3$.

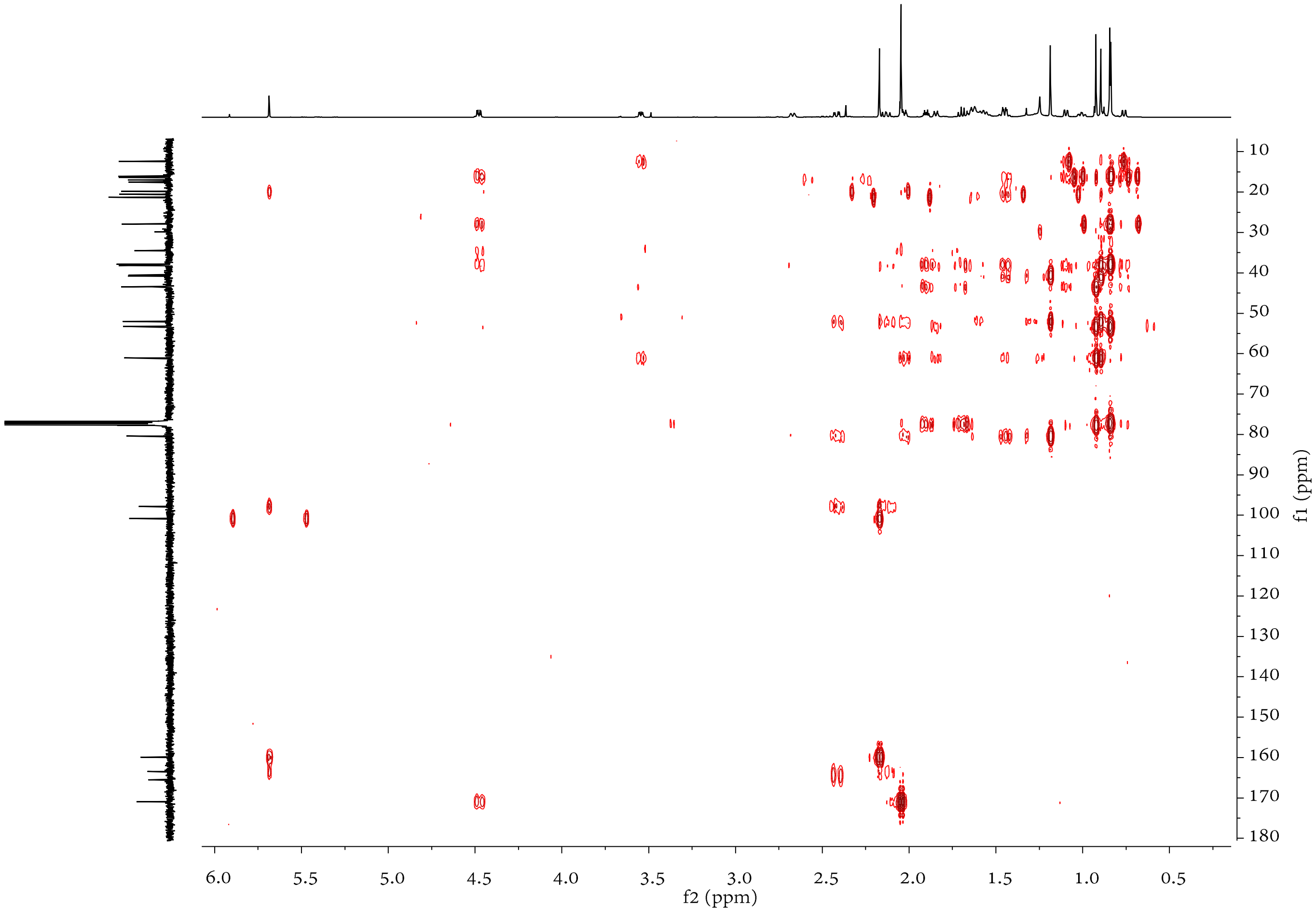

Figure S14. The HMBC spectrum of compound 2 in $CDCl_3$.

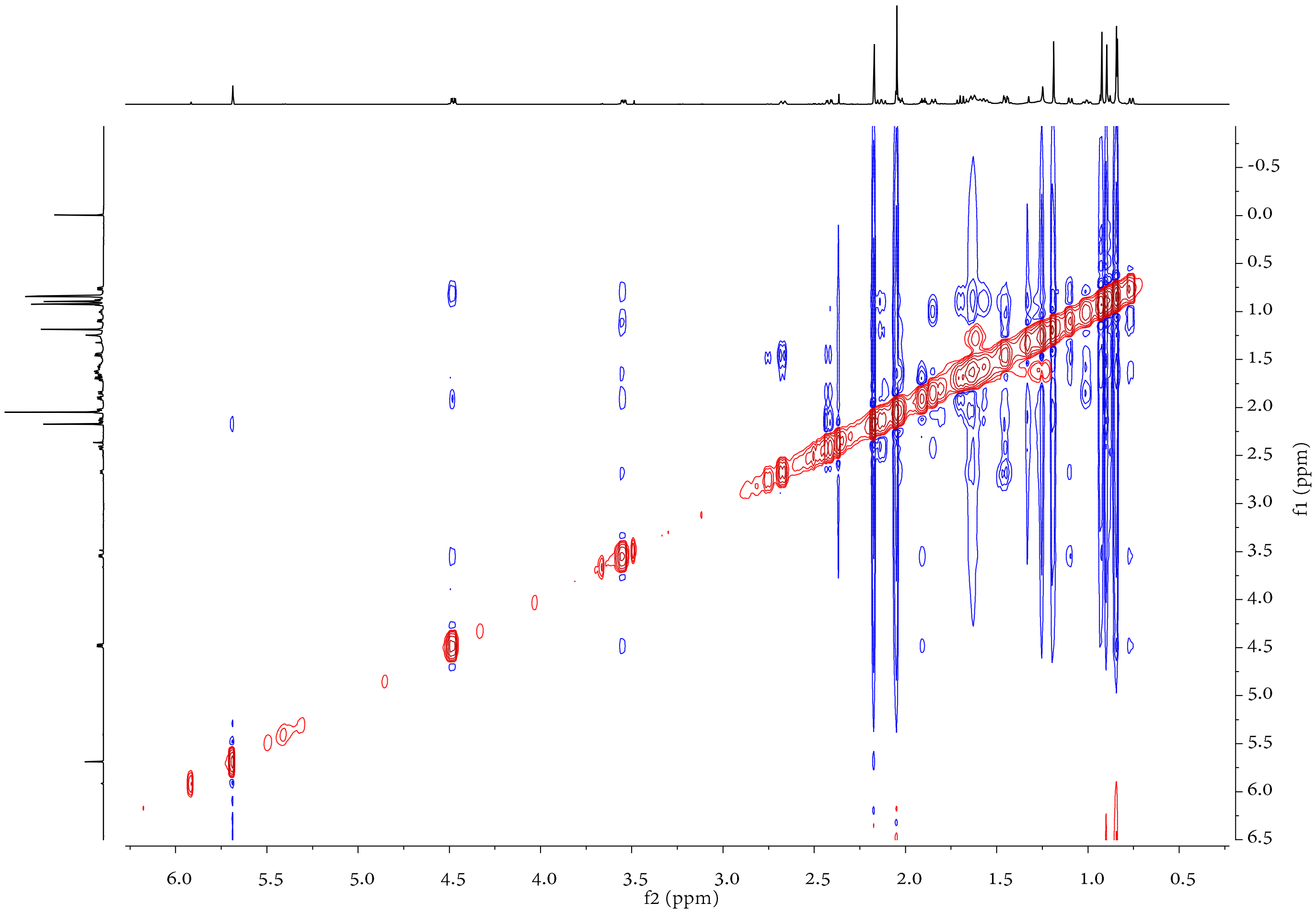

Figure S15. The NOESY spectrum of compound 2 in $CDCl_3$.

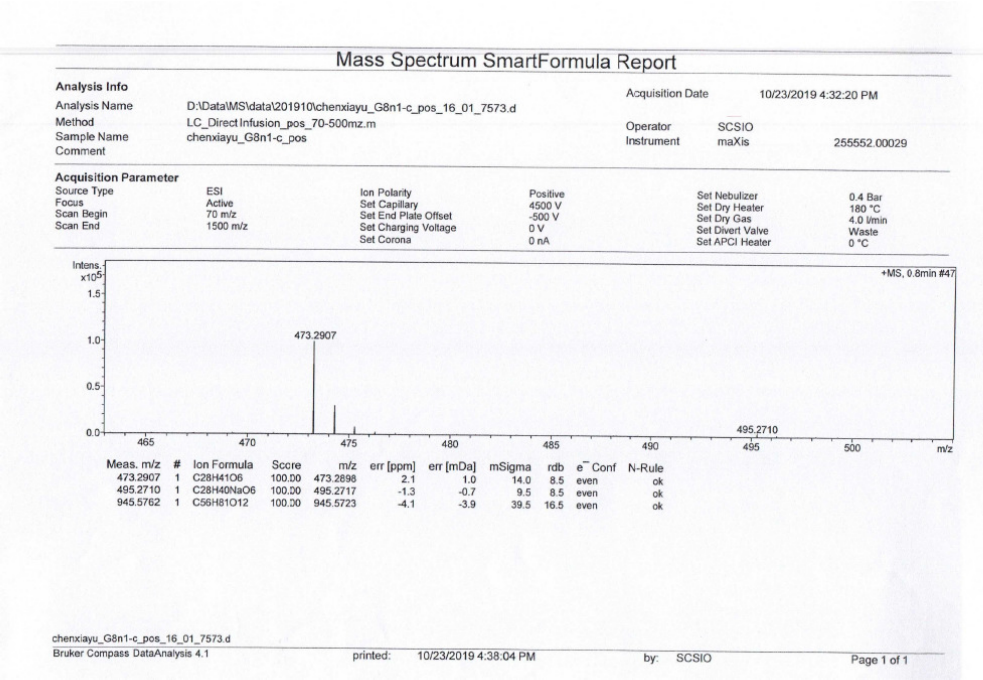

Mass Spectrum SmartFormula Report

Analysis Info

Analysis Name	D:\Data\MS\data\201910\chenxiayu_G8n1-c_pos_16_01_7573.d	Acquisition Date	10/23/2019 4:32:20 PM
Method	LC_Direct Infusion_pos_70-500mz.m	Operator	SCSIO
Sample Name	chenxiayu_G8n1-c_pos	Instrument	maXis 255552.00029
Comment			

Acquisition Parameter

Source Type	ESI	Ion Polarity	Positive	Set Nebulizer	0.4 Bar
Focus	Active	Set Capillary	4500 V	Set Dry Heater	180 °C
Scan Begin	70 m/z	Set End Plate Offset	-500 V	Set Dry Gas	4.0 l/min
Scan End	1500 m/z	Set Charging Voltage	0 V	Set Divert Valve	Waste
		Set Corona	0 nA	Set APCI Heater	0 °C

Meas. m/z	#	Ion Formula	Score	m/z	err [ppm]	err [mDa]	mSigma	rdb	e⁻ Conf	N-Rule
473.2907	1	C28H41O6	100.00	473.2898	2.1	1.0	14.0	8.5	even	ok
495.2710	1	C28H40NaO6	100.00	495.2717	-1.3	-0.7	9.5	8.5	even	ok
945.5762	1	C56H81O12	100.00	945.5723	-4.1	-3.9	39.5	16.5	even	ok

chenxiayu_G8n1-c_pos_16_01_7573.d
Bruker Compass DataAnalysis 4.1 printed: 10/23/2019 4:38:04 PM by: SCSIO Page 1 of 1

Figure S16. The HRESIMS spectrum of compound 2.

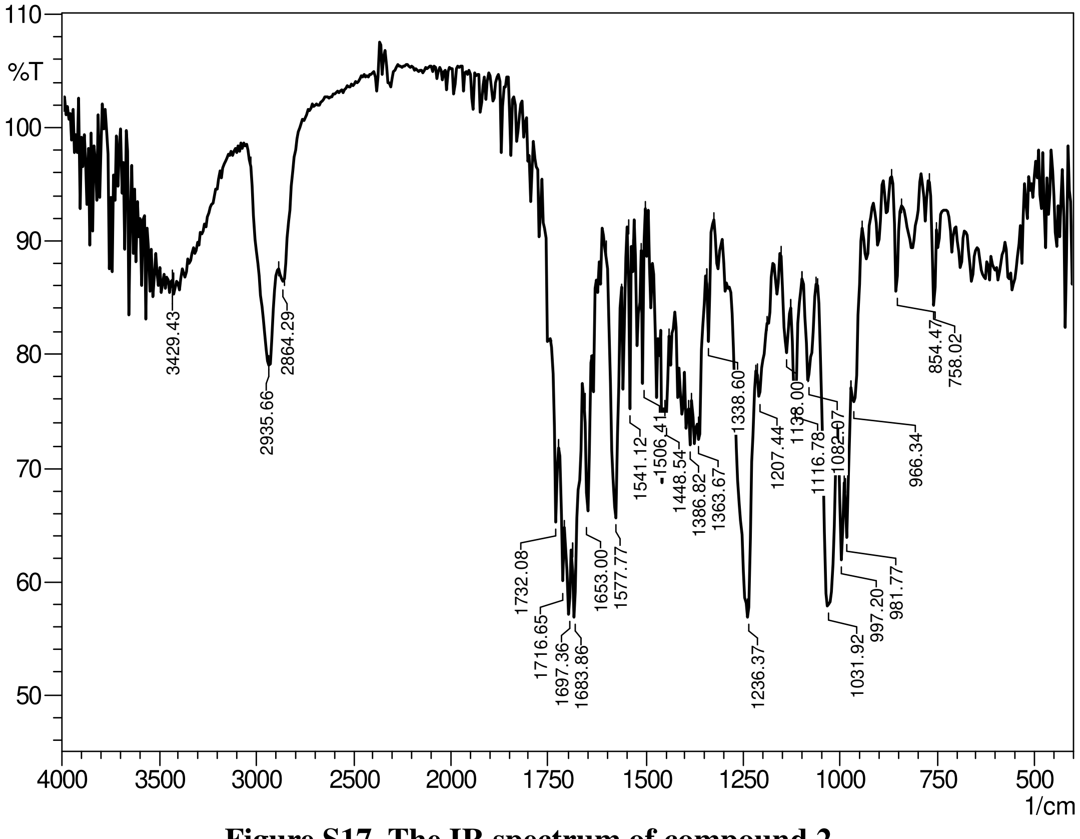

Figure S17. The IR spectrum of compound 2.

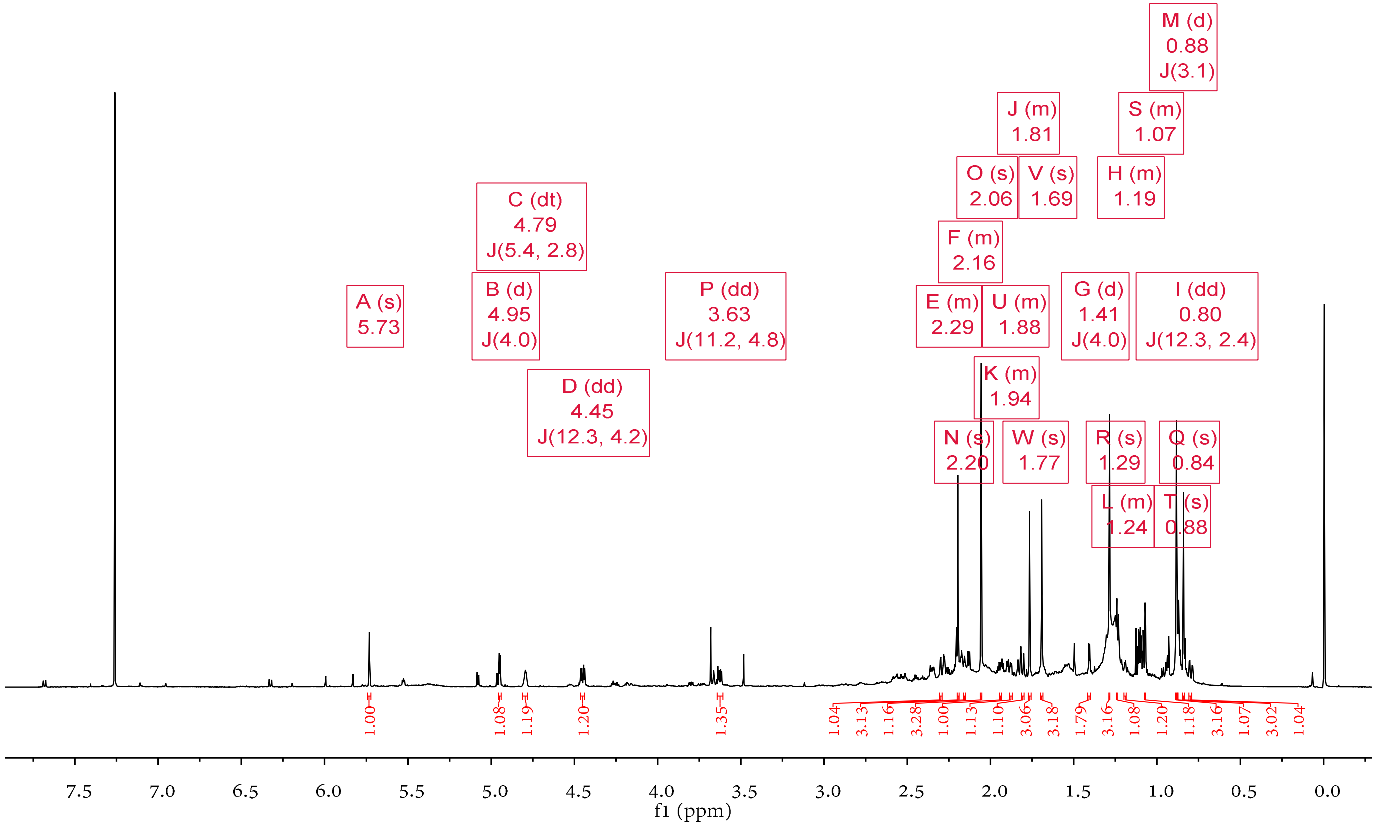

Figure S18. The ^{1}H spectrum of compound 3 in $CDCl_3$.

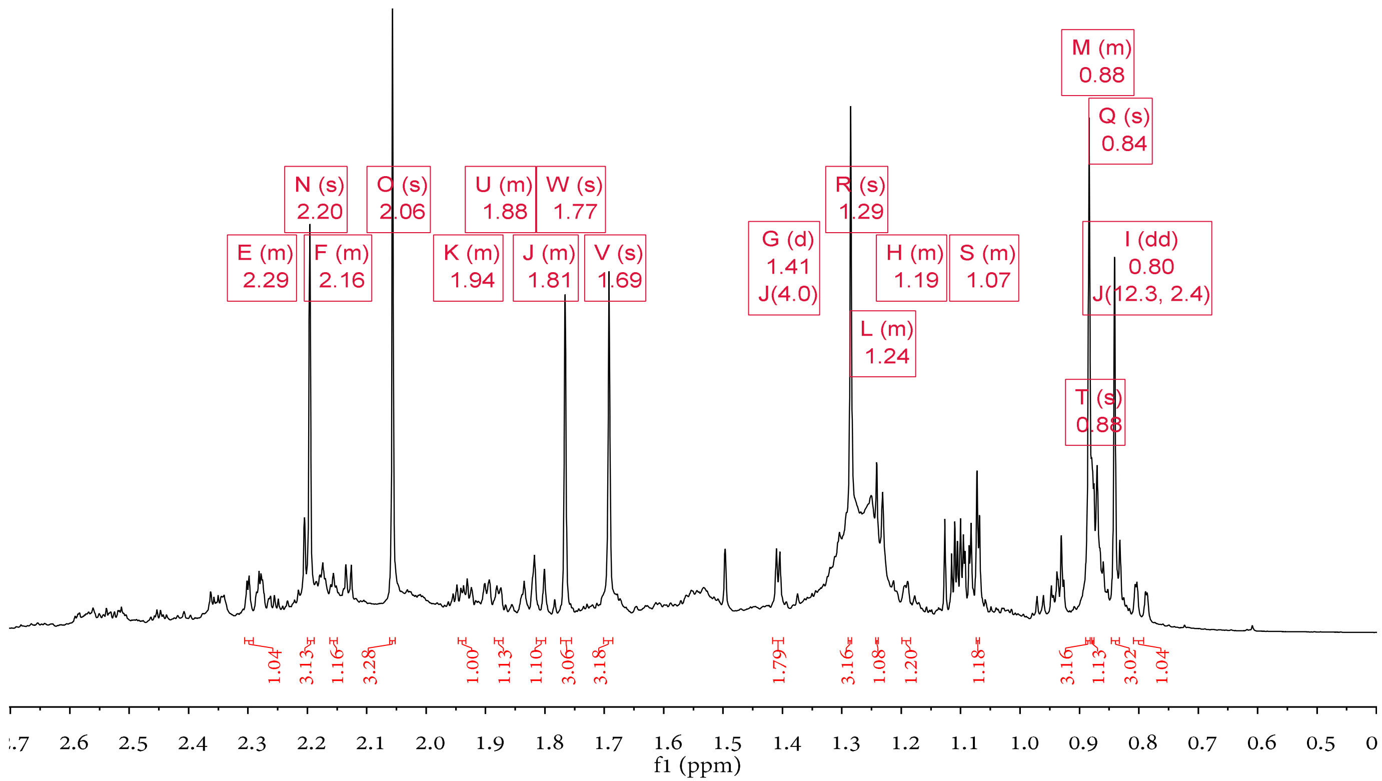

Figure S19. The ^{1}H spectrum (0-3 ppm) of compound 3 in $CDCl_3$.

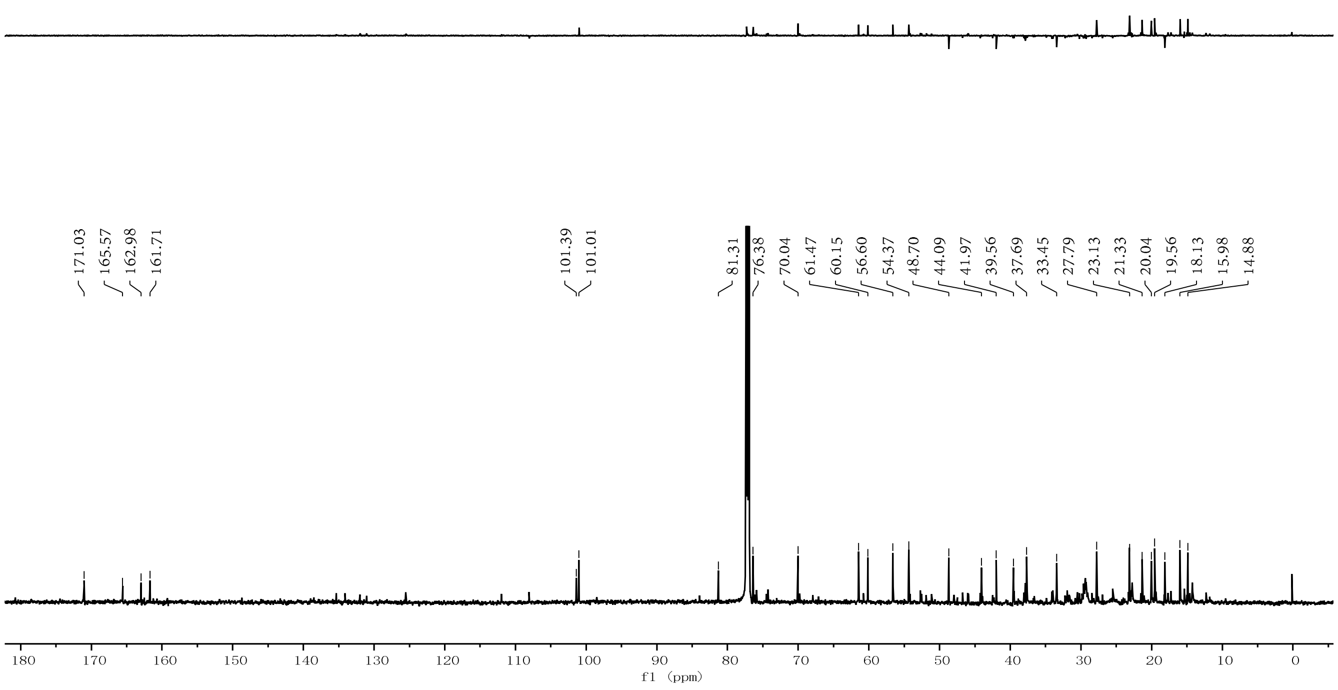

Figure S20. The ^{13}C spectrum of compound 3 in $CDCl_3$.

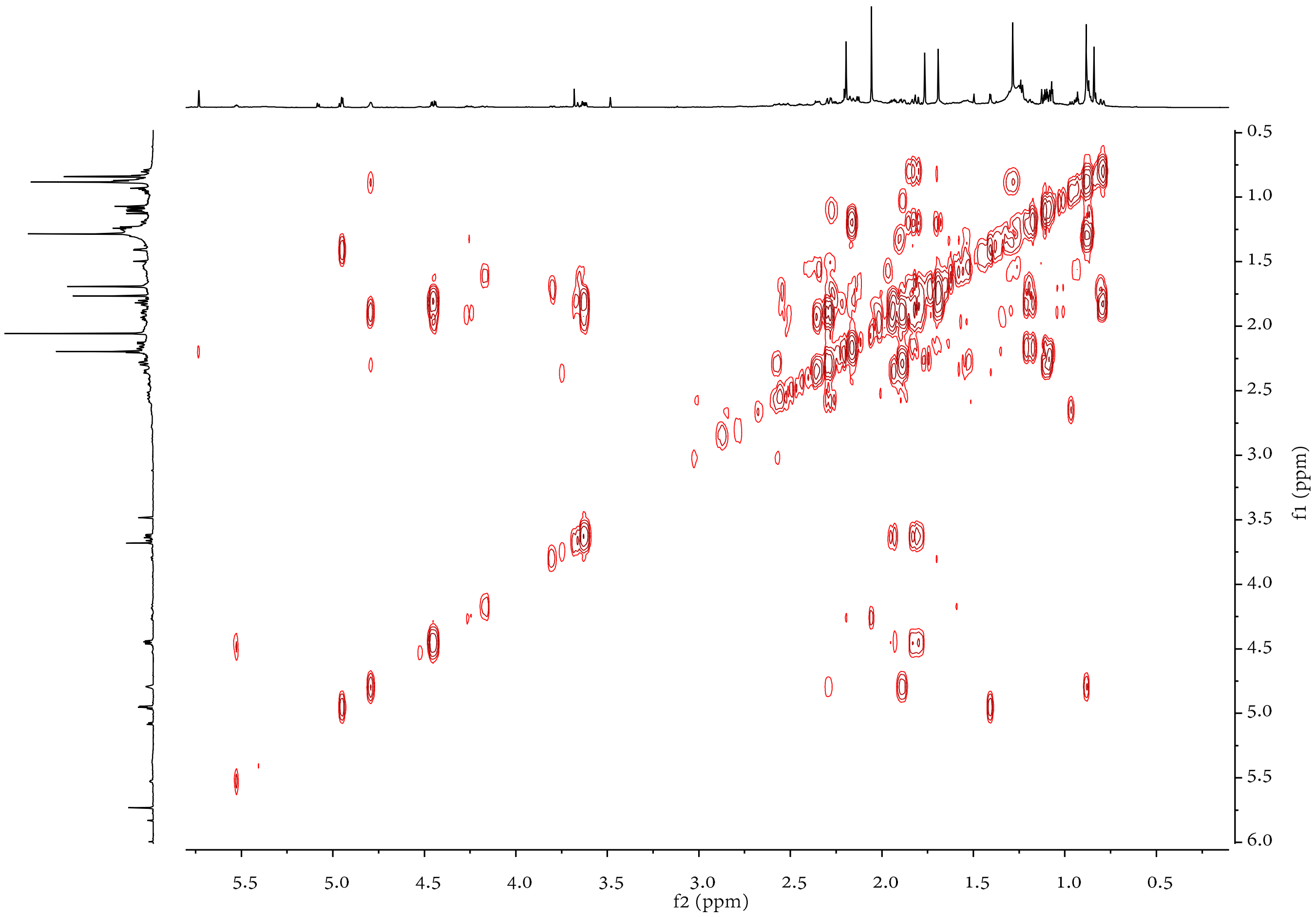

Figure S21. The ^{1}H-^{1}H COSY spectrum of compound 3 in $CDCl_3$.

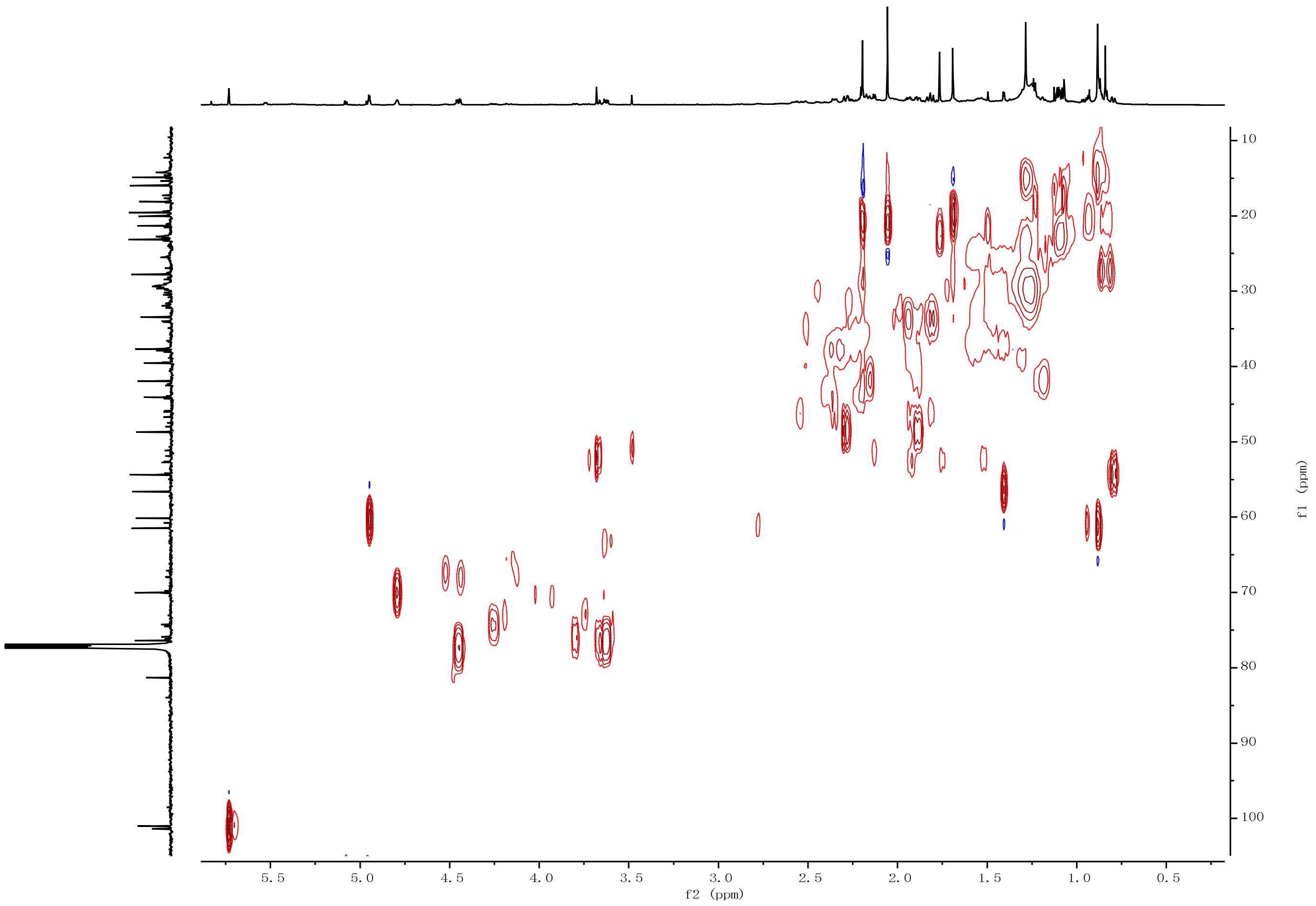

Figure S22. The HSQC spectrum of compound 3 in $CDCl_3$.

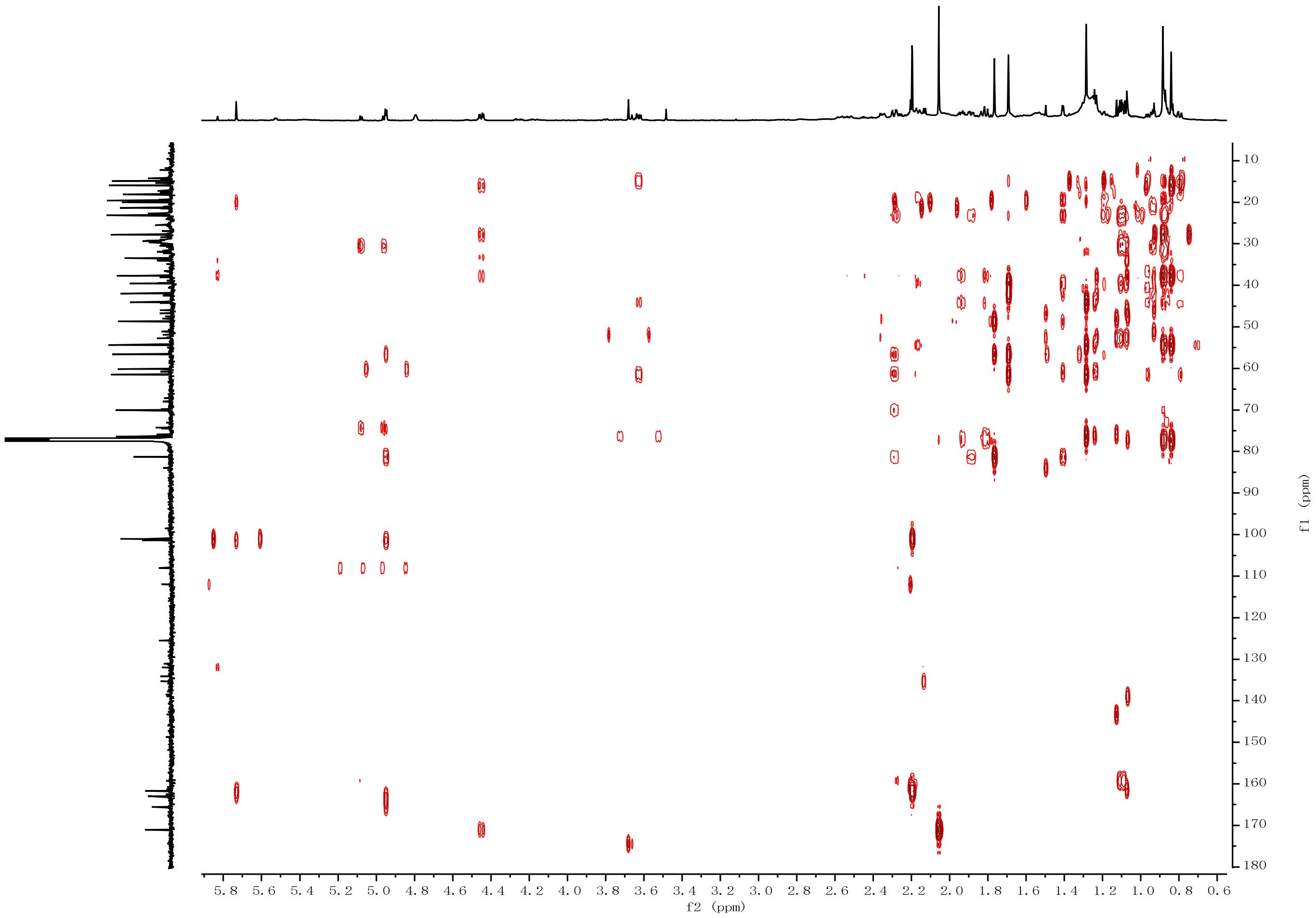

Figure S23. The HMBC spectrum of compound 3 in $CDCl_3$.

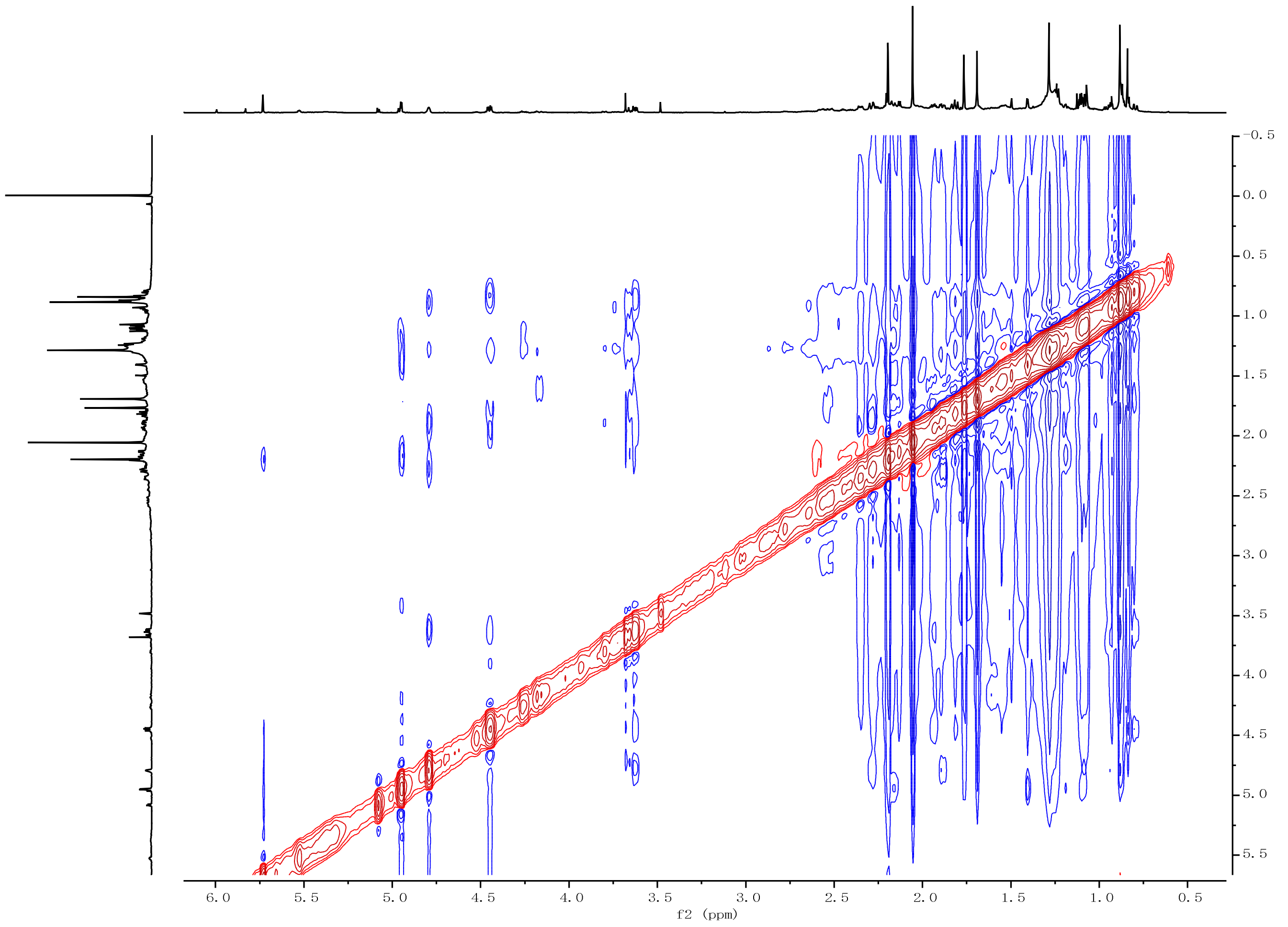

Figure S24. The NOESY spectrum of compound 3 in $CDCl_3$.

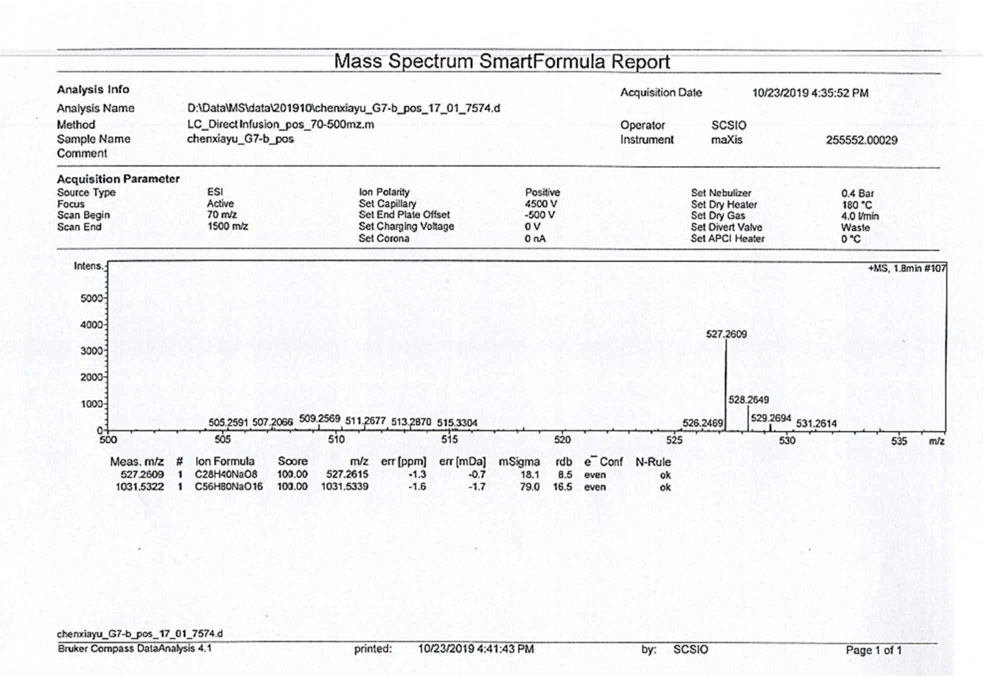

Mass Spectrum SmartFormula Report

**Analysis Info**		Acquisition Date	10/23/2019 4:35:52 PM		
Analysis Name	D:\Data\MS\data\201910\chenxiayu_G7-b_pos_17_01_7574.d				
Method	LC_Direct Infusion_pos_70-500mz.m	Operator	SCSIO		
Sample Name	chenxiayu_G7-b_pos	Instrument	maXis	255552.00029	
Comment					

Acquisition Parameter

Source Type	ESI	Ion Polarity	Positive	Set Nebulizer	0.4 Bar
Focus	Active	Set Capillary	4500 V	Set Dry Heater	180 °C
Scan Begin	70 m/z	Set End Plate Offset	-500 V	Set Dry Gas	4.0 l/min
Scan End	1500 m/z	Set Charging Voltage	0 V	Set Divert Valve	Waste
		Set Corona	0 nA	Set APCI Heater	0 °C

Meas. m/z	#	Ion Formula	Score	m/z	err [ppm]	err [mDa]	mSigma	rdb	e⁻ Conf	N-Rule
527.2609	1	C28H40NaO8	100.00	527.2615	-1.3	-0.7	18.1	8.5	even	ok
1031.5322	1	C56H80NaO16	100.00	1031.5339	-1.6	-1.7	79.0	16.5	even	ok

chenxiayu_G7-b_pos_17_01_7574.d

Bruker Compass DataAnalysis 4.1 printed: 10/23/2019 4:41:43 PM by: SCSIO Page 1 of 1

Figure S25. The HRESIMS spectra of compound 3.

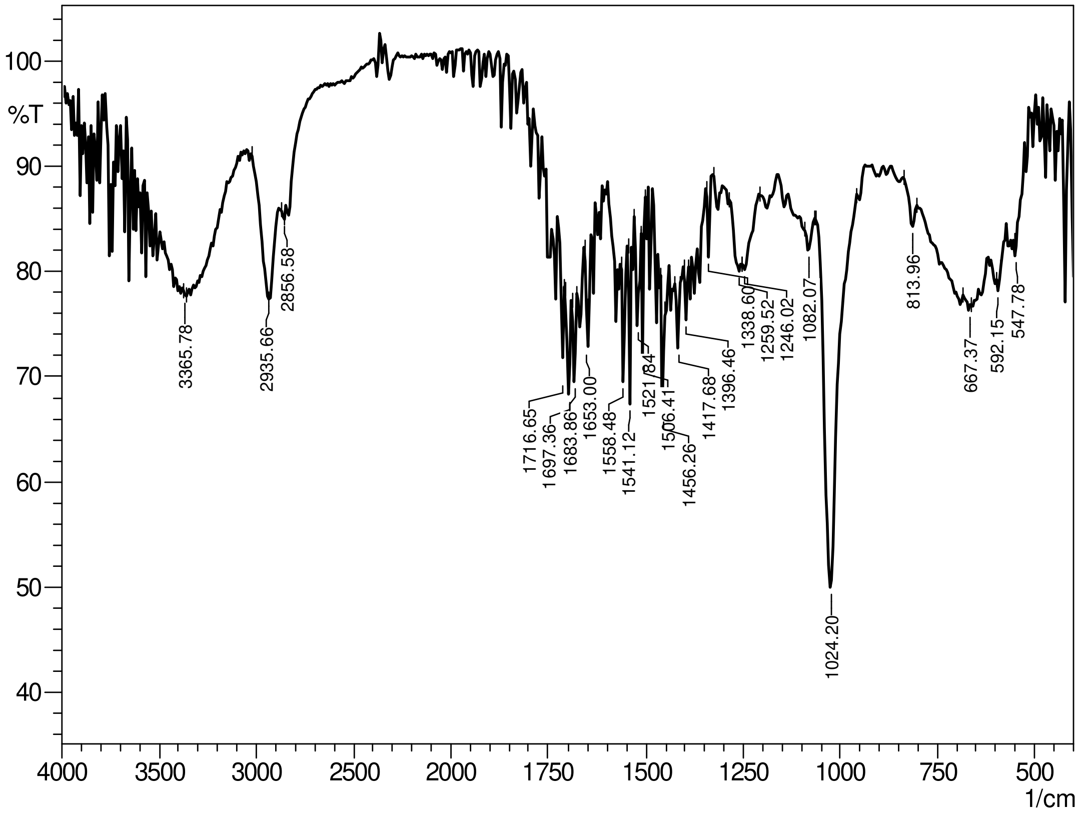

Figure S26. The IR spectrum of compound 3.

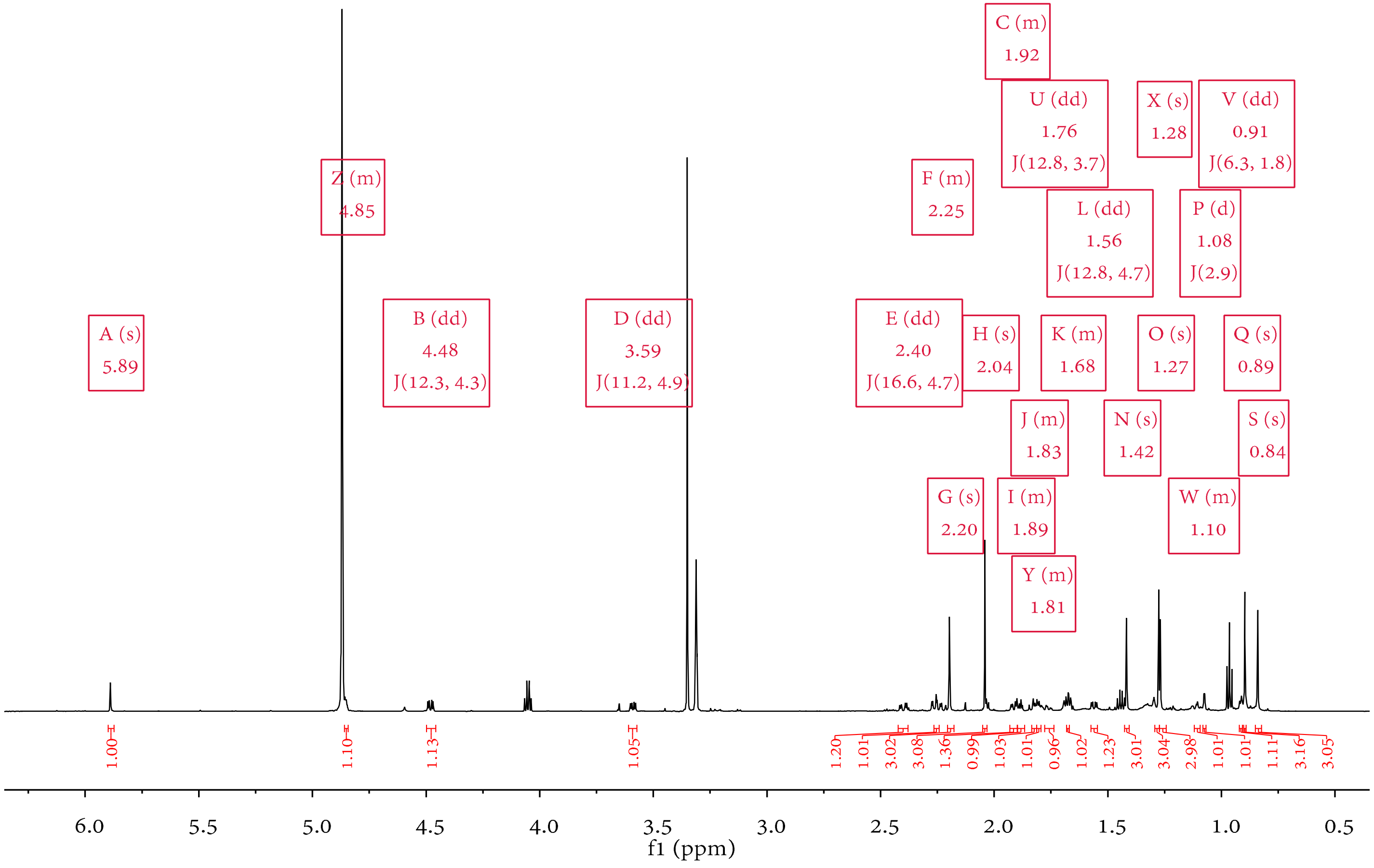

Figure S27. The ^{1}H NMR spectrum of compound 4 in CD_3OD.

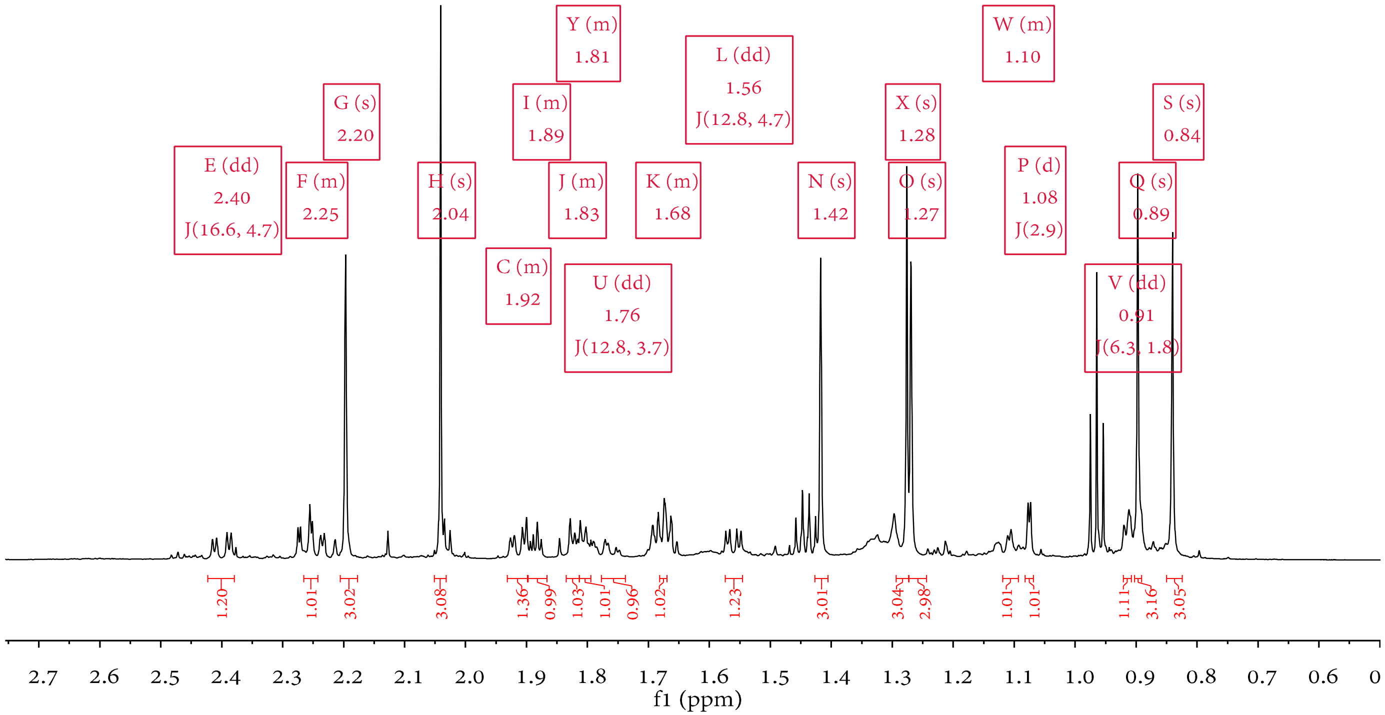

Figure S28. The ^{1}H NMR spectrum (0-3 ppm) of compound 4 in CD_3OD.

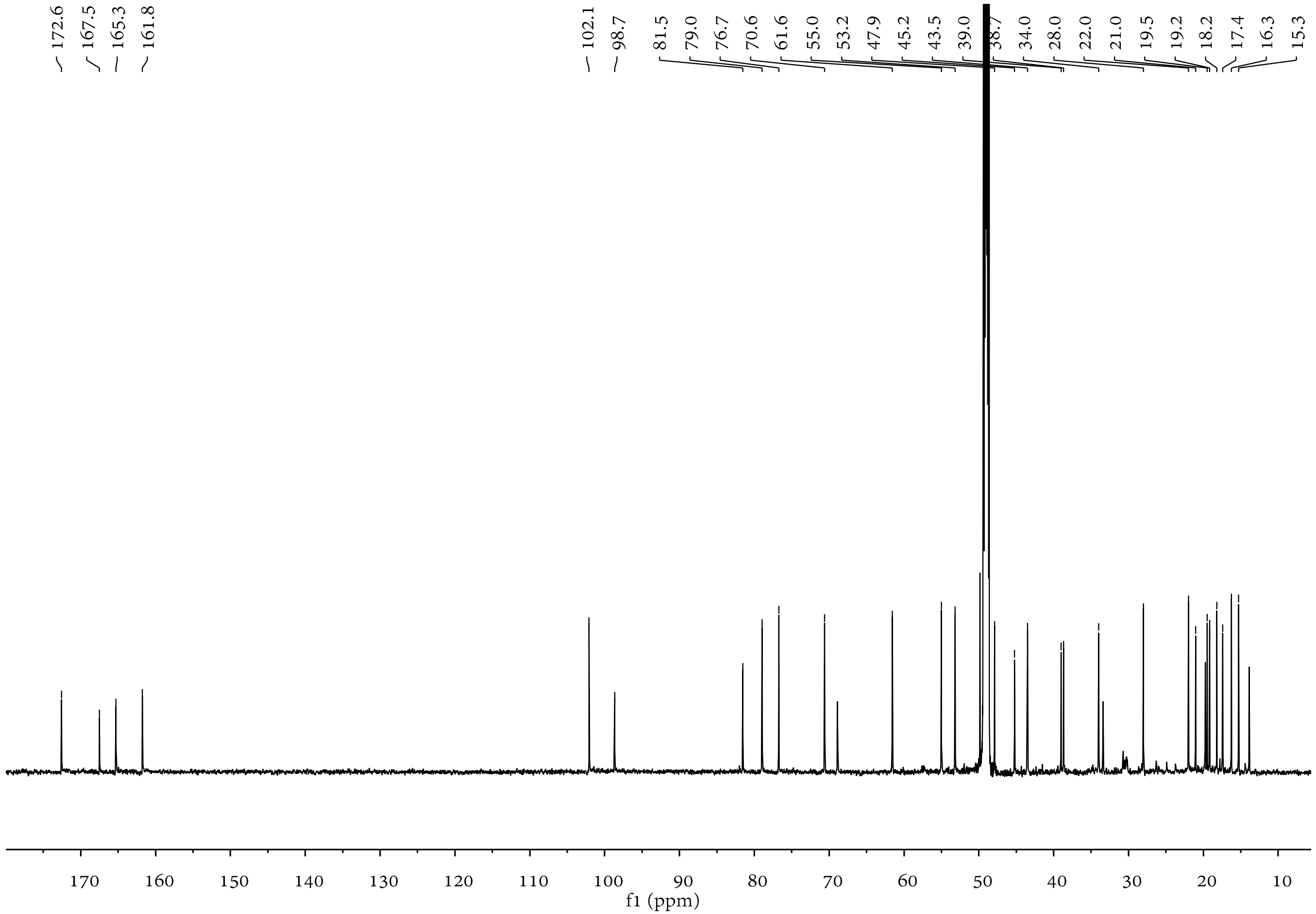

Figure S29. The ^{13}C NMR spectrum of compound 4 in CD_3OD.

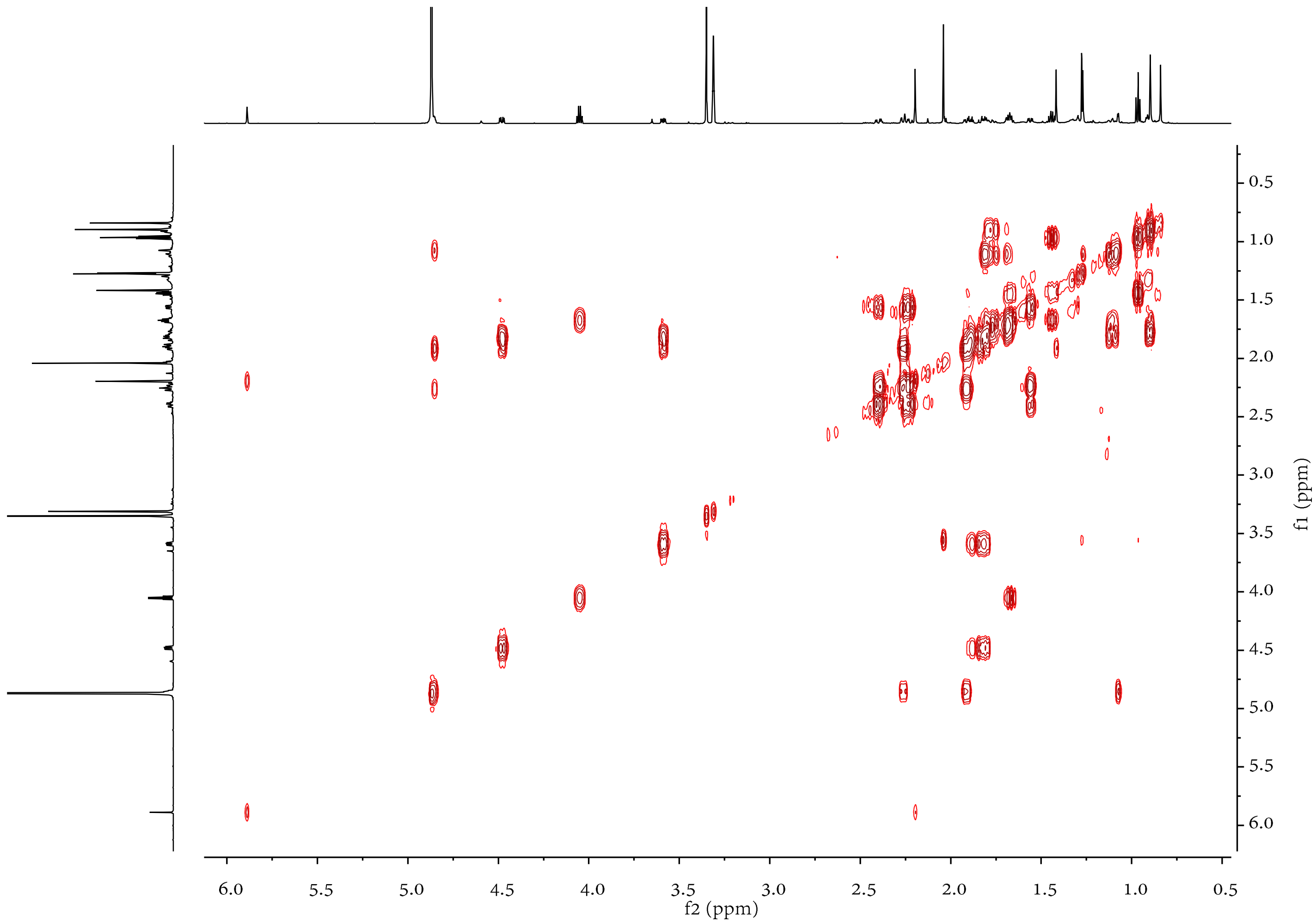

Figure S30. The ^{1}H-^{1}H COSY spectrum of compound 4 in CD_3OD.

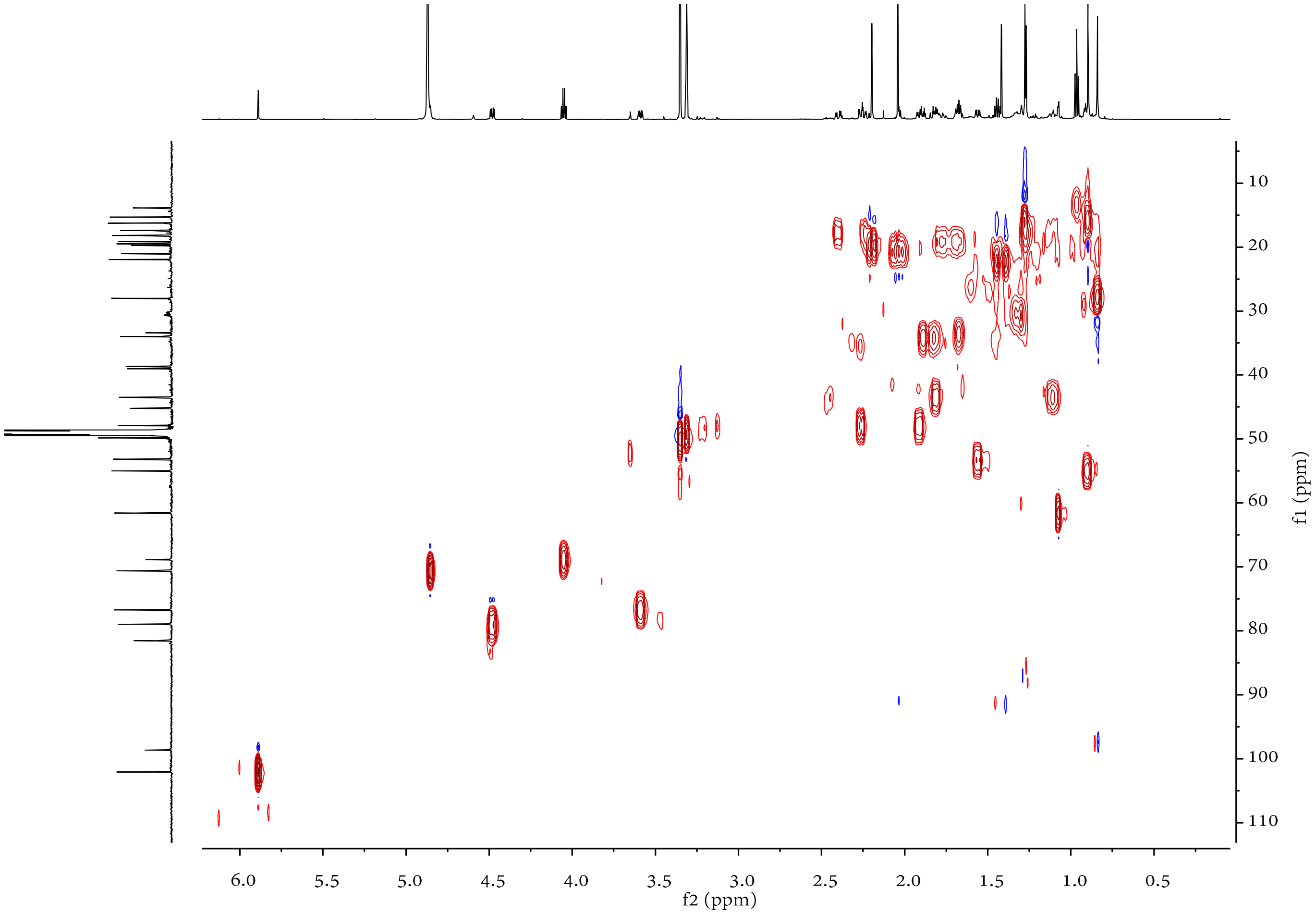

Figure S31. The HSQC spectrum of compound 4 in CD_3OD.

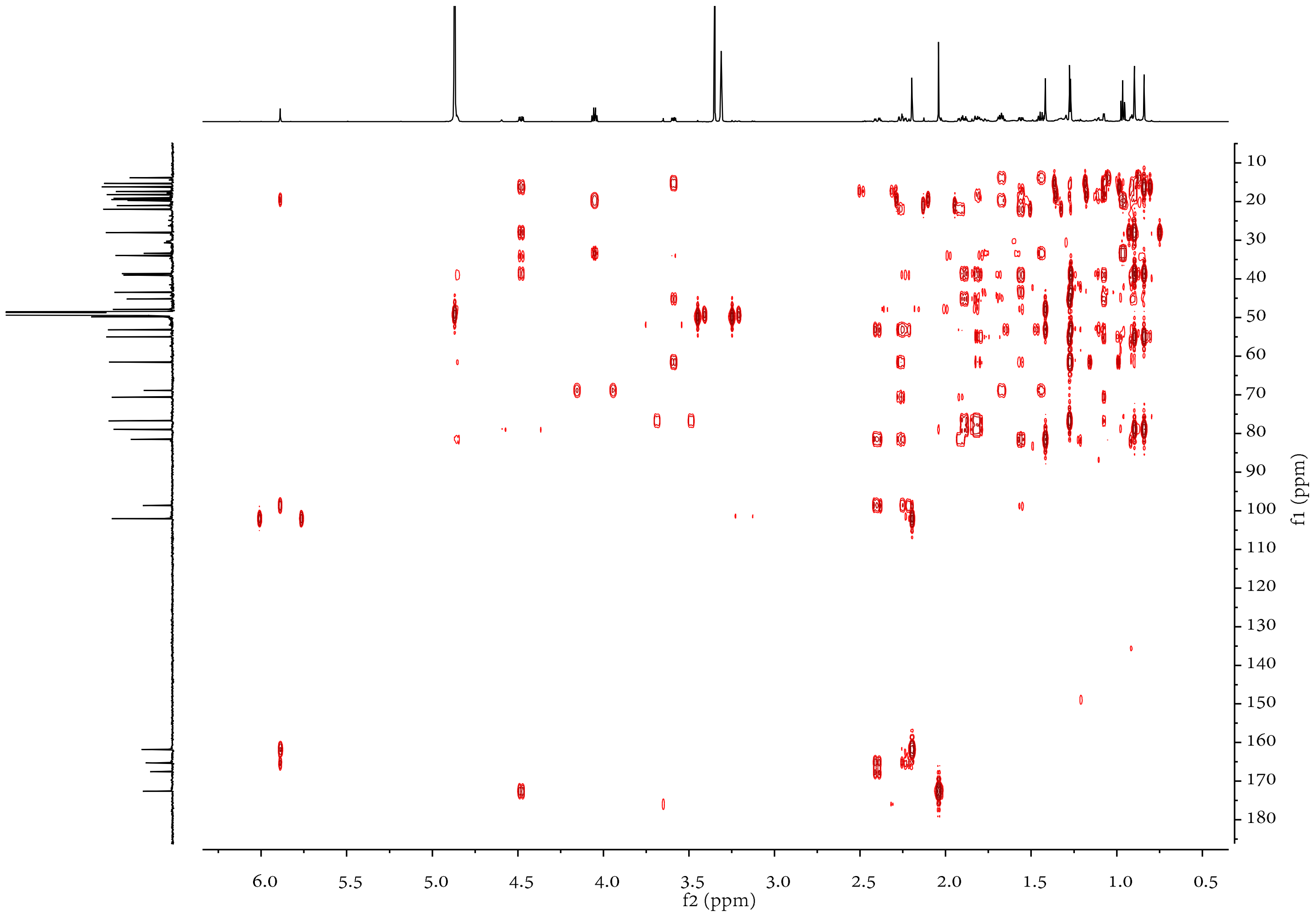

Figure S32. The HMBC spectrum of compound 4 in CD_3OD.

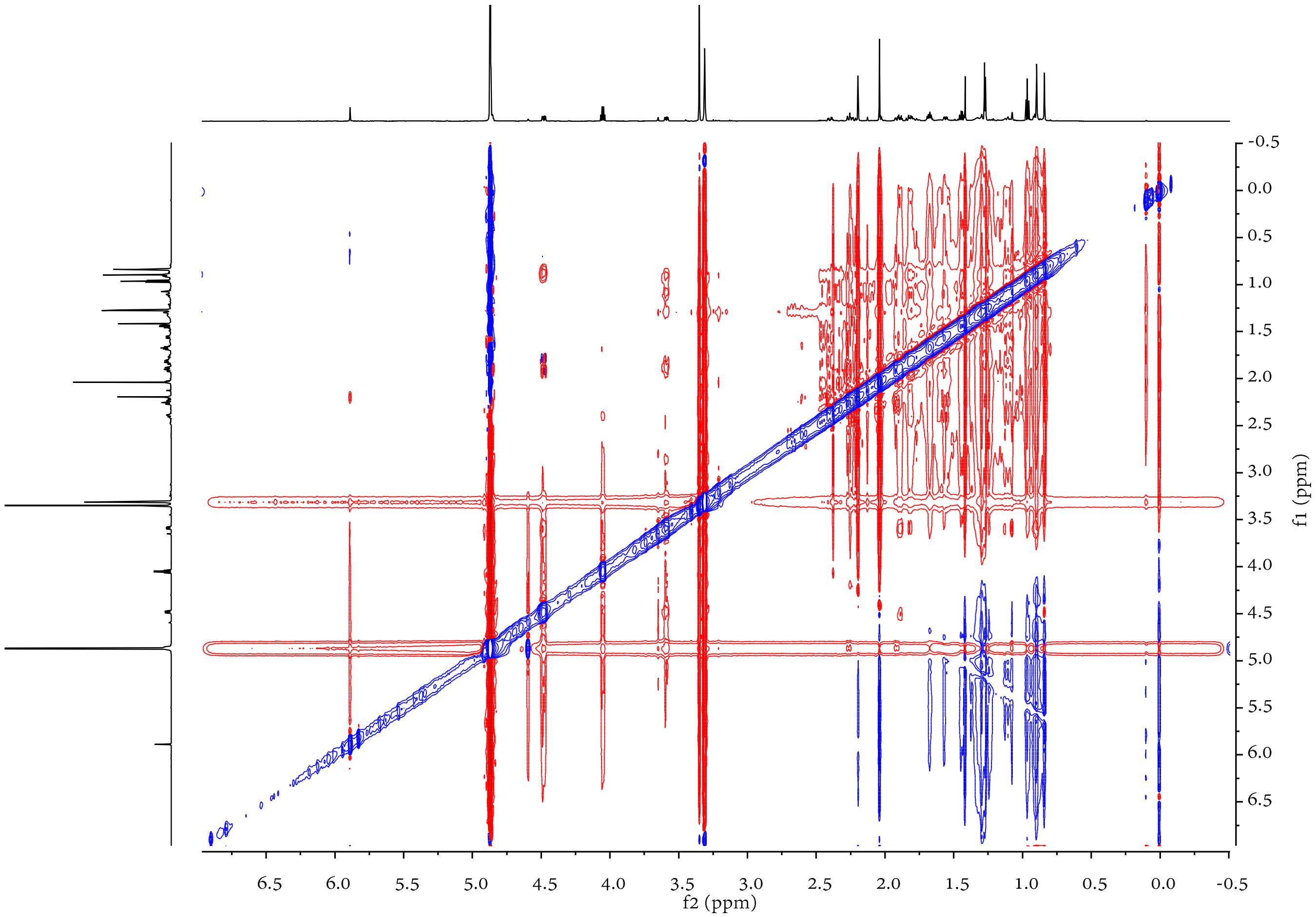

Figure S33. The NOESY spectrum of compound 4 in CD_3OD.

Mass Spectrum SmartFormula Report

Analysis Info

Analysis Name	D:\Data\MS\data\201911\chenxiayu_G8n1-a2_pos_21_01_7673.d	Acquisition Date	11/15/2019 9:59:36 AM
Method	LC_Direct Infusion_pos_70-500mz.m	Operator	SCSIO
Sample Name	chenxiayu_G8n1-a2_pos	Instrument	maXis 255552.00029
Comment			

Acquisition Parameter

Source Type	ESI	Ion Polarity	Positive	Set Nebulizer	0.4 Bar
Focus	Active	Set Capillary	4500 V	Set Dry Heater	180 °C
Scan Begin	70 m/z	Set End Plate Offset	-500 V	Set Dry Gas	4.0 l/min
Scan End	1500 m/z	Set Charging Voltage	0 V	Set Divert Valve	Waste
		Set Corona	0 nA	Set APCI Heater	0 °C

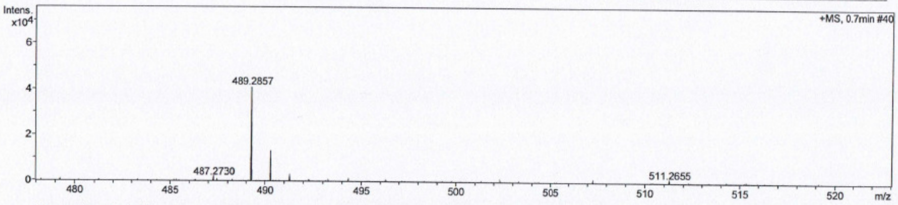

Meas. m/z	#	Ion Formula	Score	m/z	err [ppm]	err [mDa]	mSigma	rdb	e⁻ Conf	N-Rule
489.2857	1	C28H41O7	100.00	489.2847	2.2	1.1	7.9	8.5	even	ok
511.2655	1	C28H40NaO7	100.00	511.2666	-2.3	-1.2	30.8	8.5	even	ok
977.5629	1	C56H81O14	100.00	977.5621	0.8	0.8	31.8	16.5	even	ok
999.5433	1	C56H80NaO14	100.00	999.5440	-0.8	-0.8	166.3	16.5	even	ok

Figure S34. The HRESIMS spectrum of compound 4.

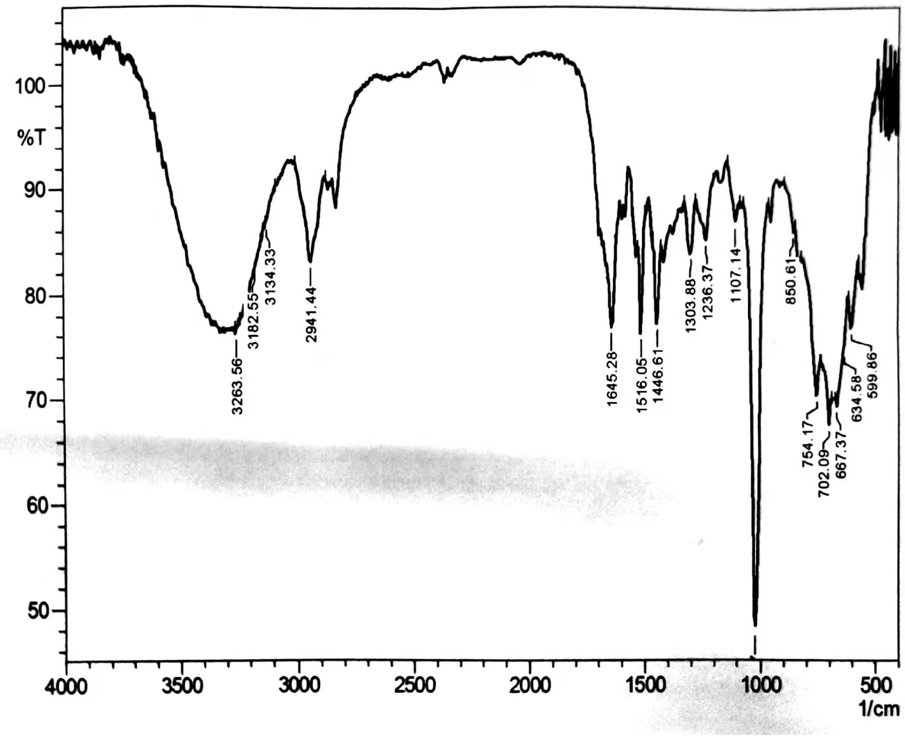

Figure S35. The IR spectrum of compound 4.

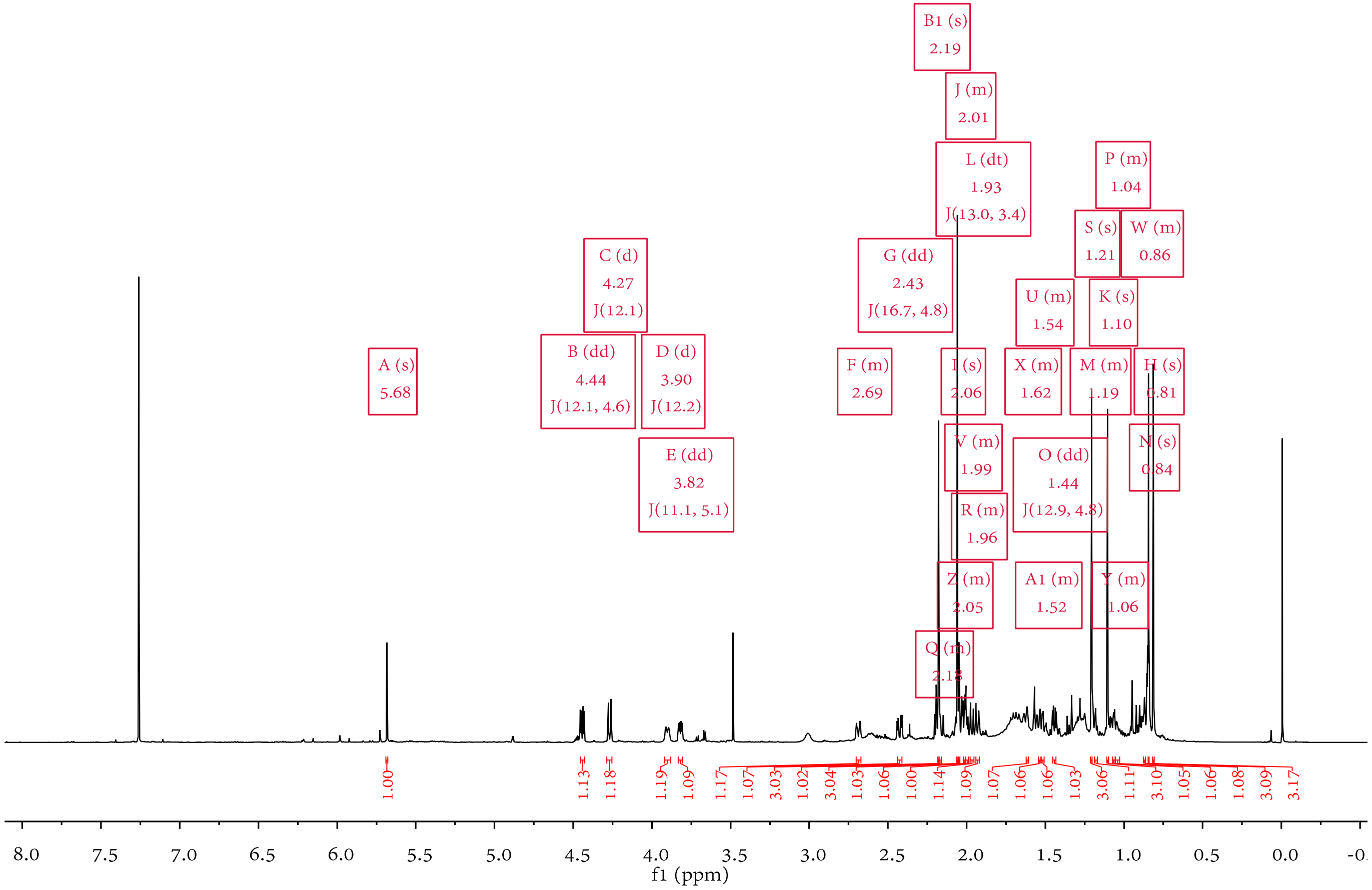

Figure S36. The ^{1}H NMR spectrum of compound 5 in $CDCl_3$.

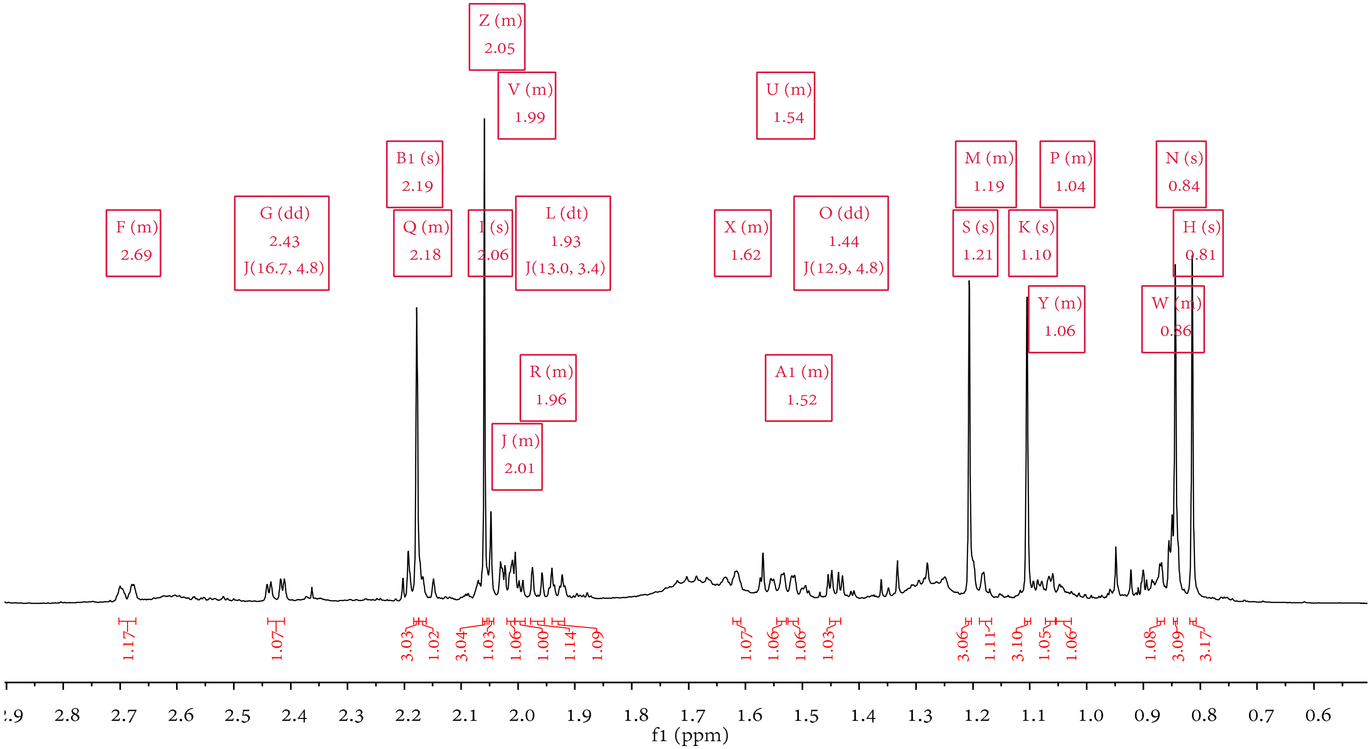

Figure S37. The ^{1}H NMR spectrum (0-3 ppm) of compound 5 in $CDCl_3$.

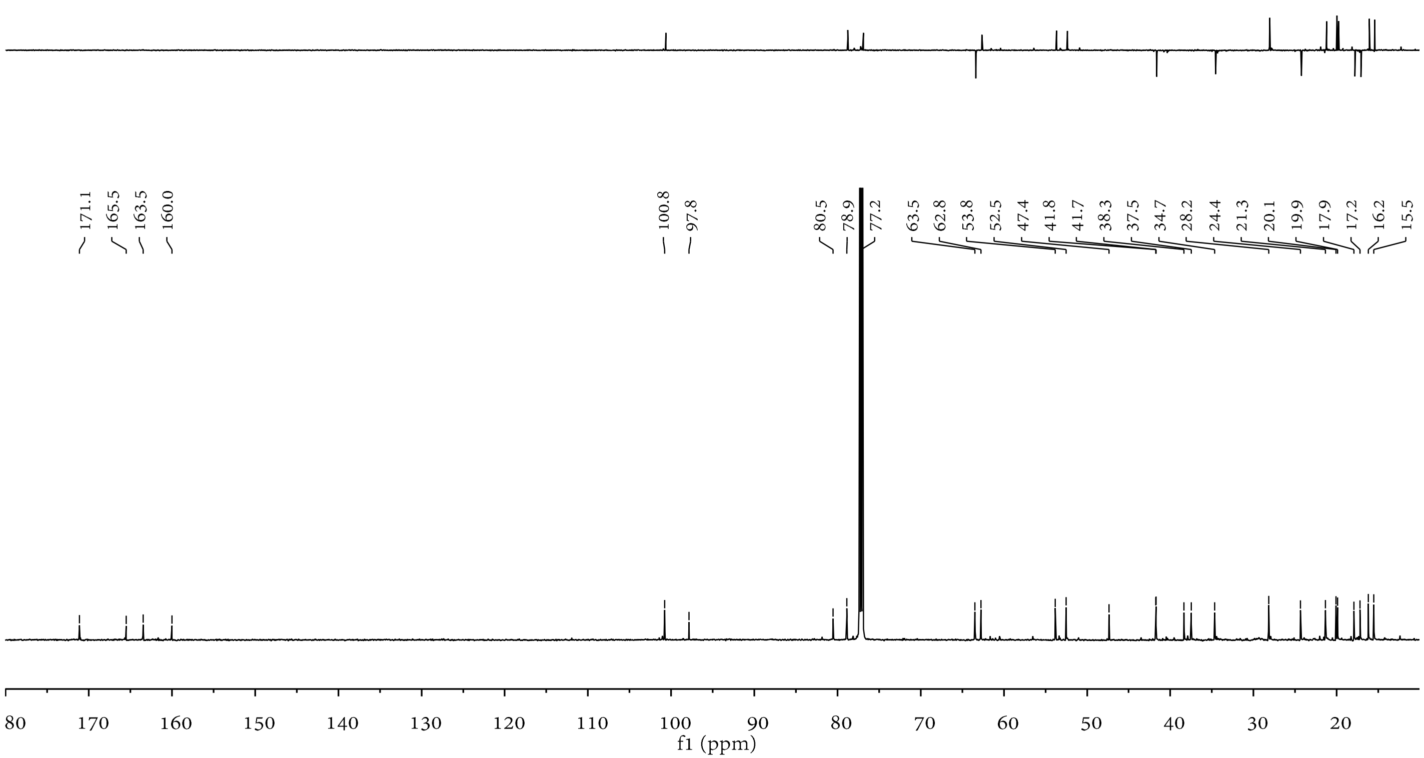

Figure S38. The ^{13}C NMR spectrum of compound 5 in $CDCl_3$.

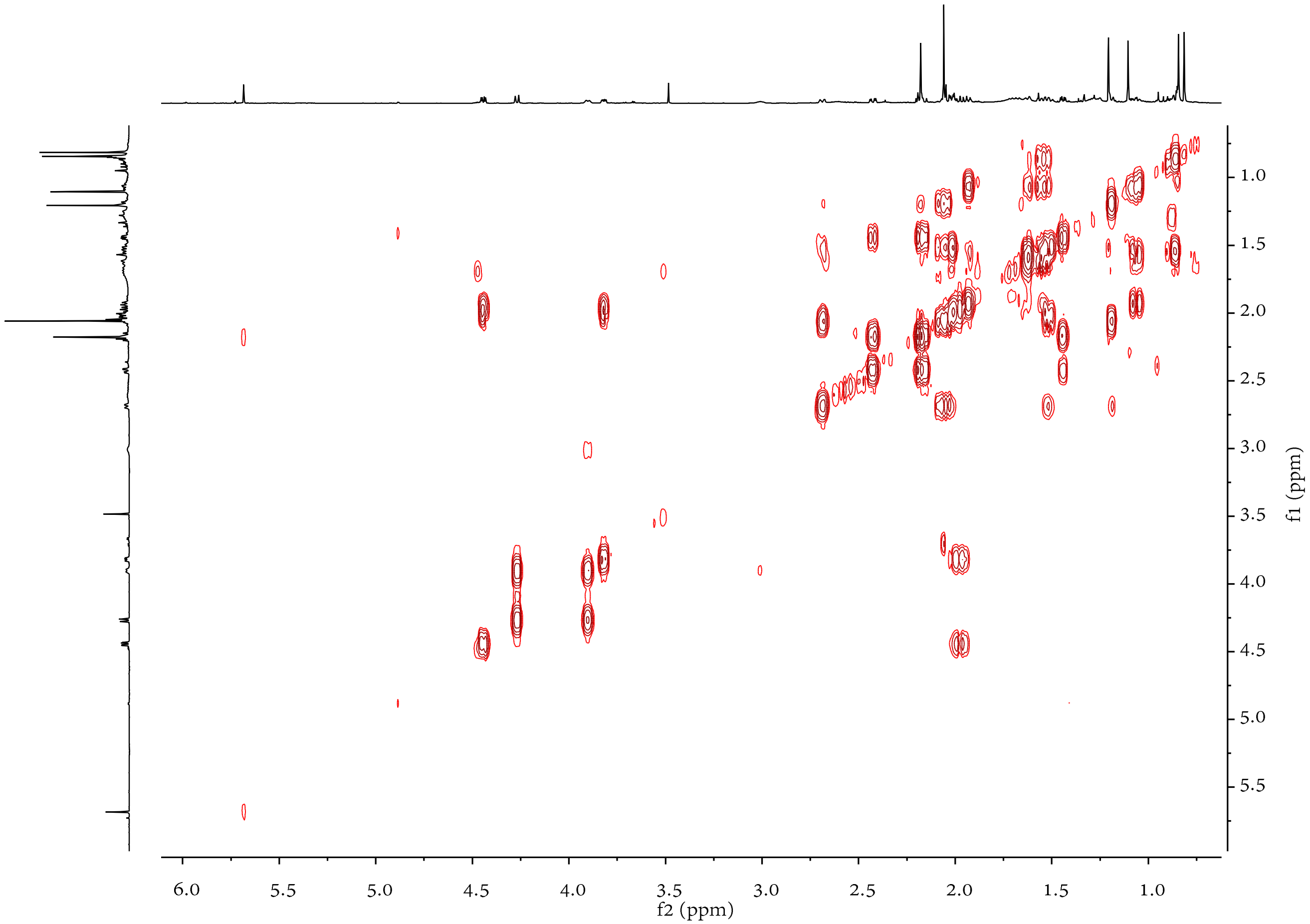

Figure S39. The ^{1}H-^{1}H COSY spectrum of compound 5 in $CDCl_3$.

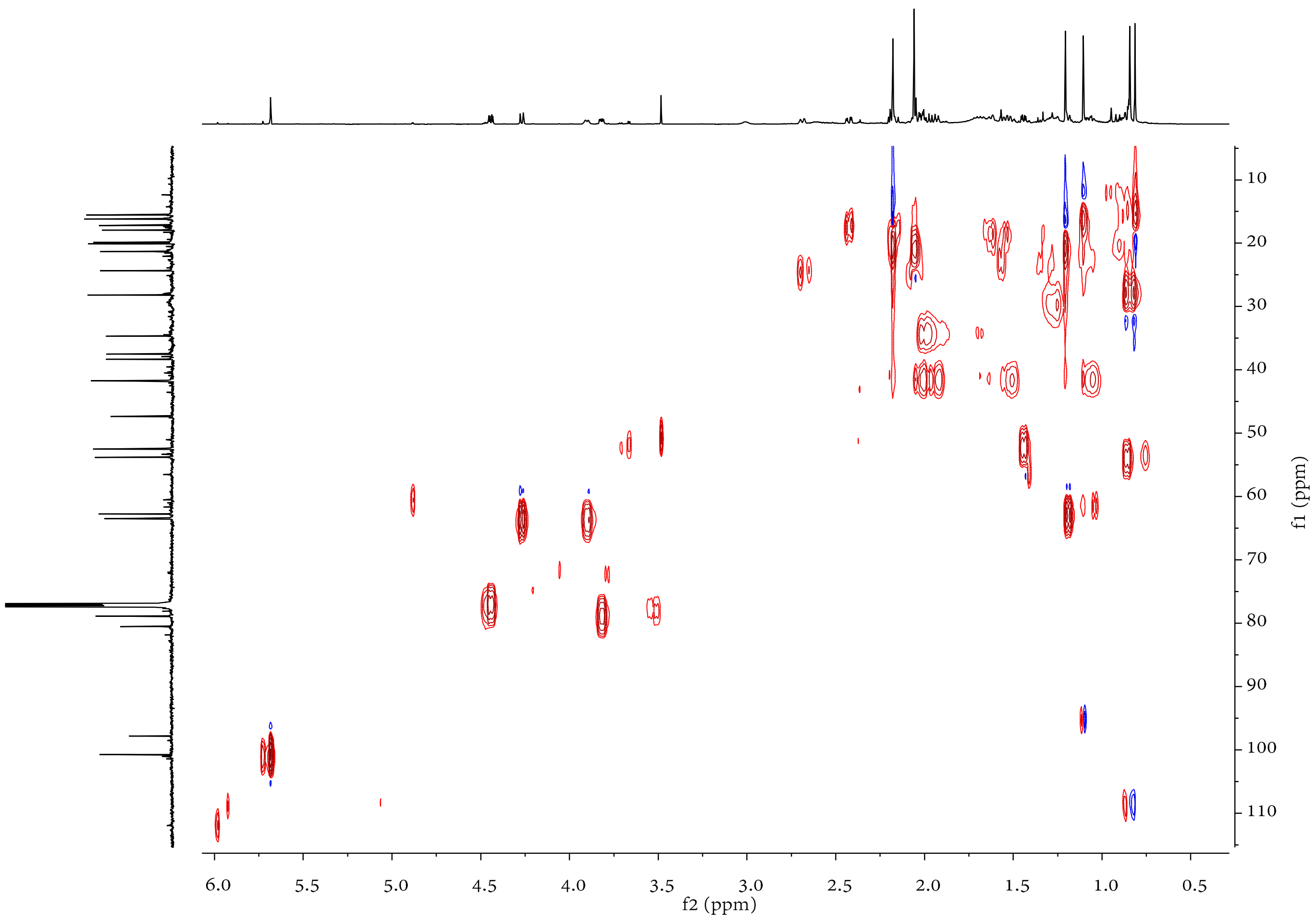

Figure S40. The HSQC spectrum of compound 5 in $CDCl_3$.

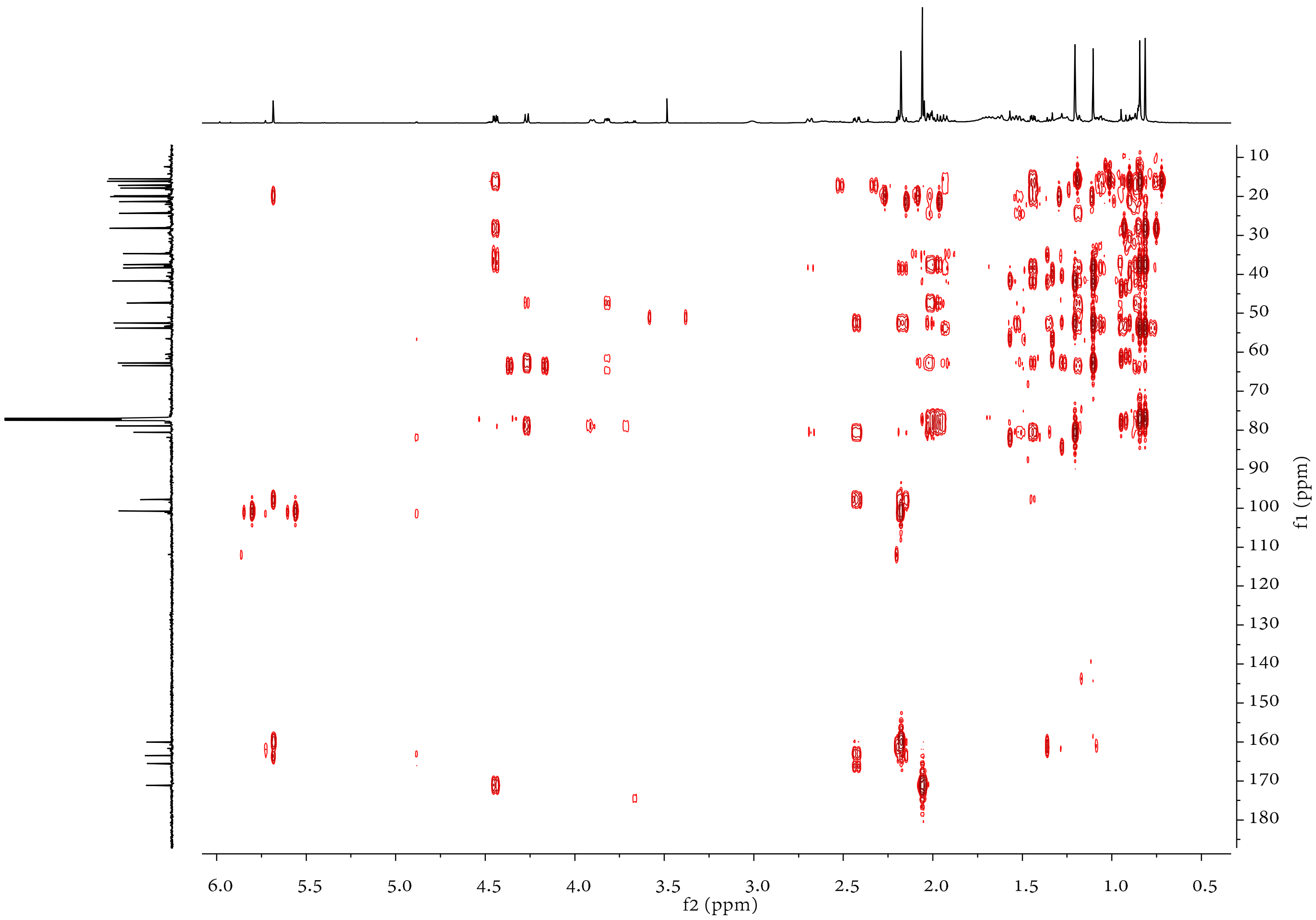

Figure S41. The HMBC spectrum of compound 5 in $CDCl_3$.

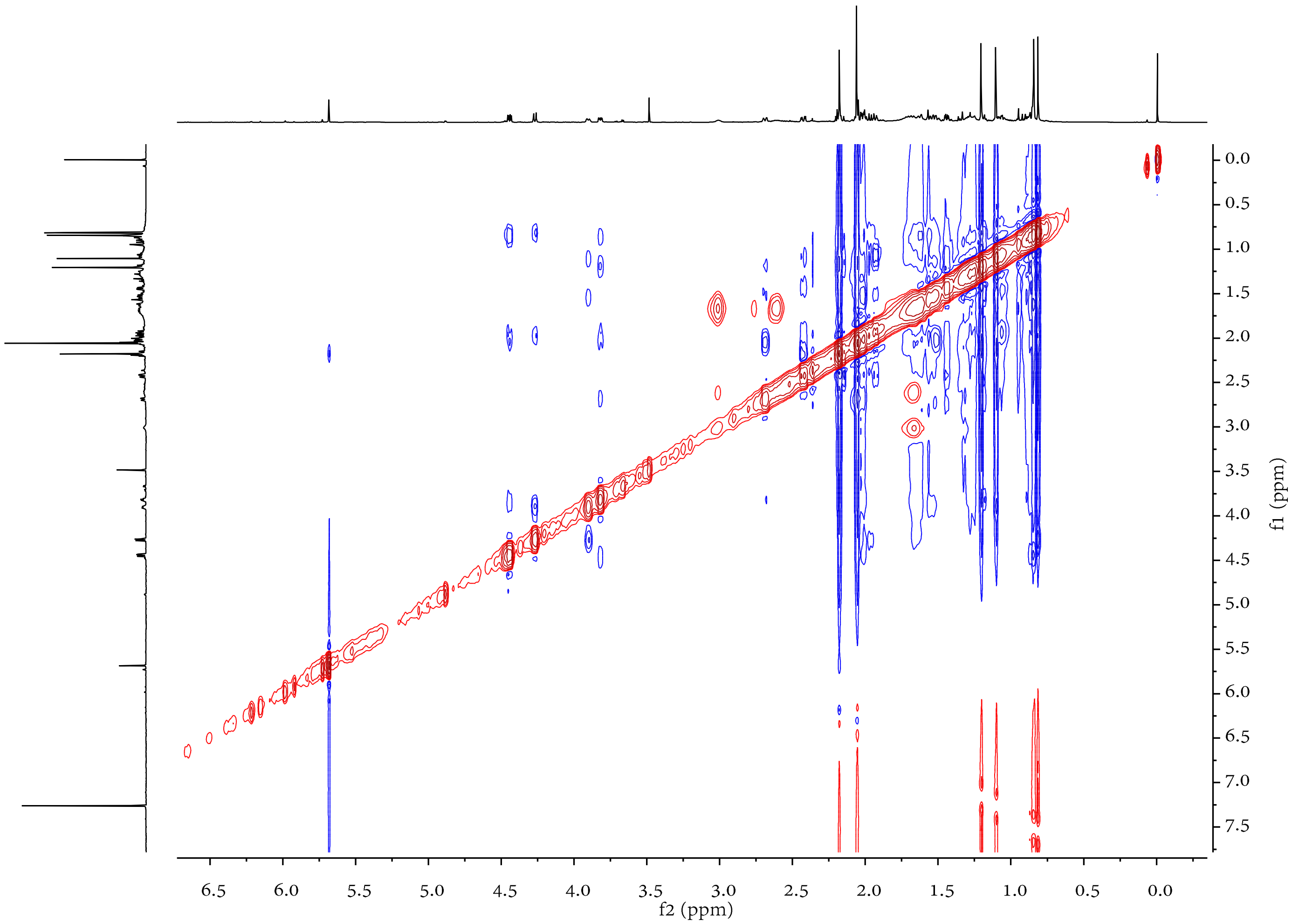

Figure S42. The NOESY spectrum of compound 5 in $CDCl_3$.

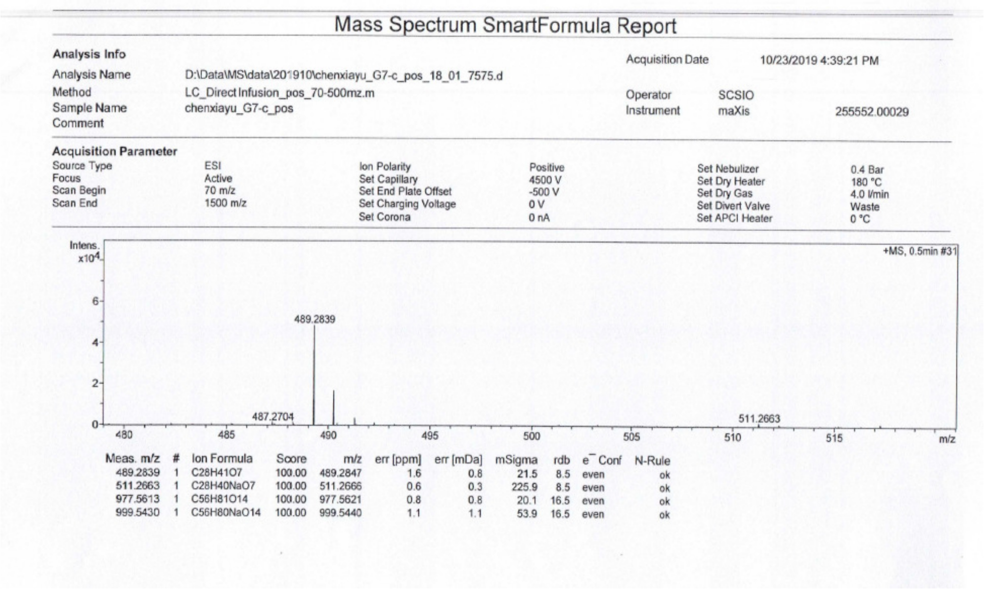

Mass Spectrum SmartFormula Report

Analysis Info

Analysis Name	D:\Data\MS\data\201910\chenxiayu_G7-c_pos_18_01_7575.d	Acquisition Date	10/23/2019 4:39:21 PM
Method	LC_Direct Infusion_pos_70-500mz.m	Operator	SCSIO
Sample Name	chenxiayu_G7-c_pos	Instrument	maXis 255552.00029
Comment			

Acquisition Parameter

Source Type	ESI	Ion Polarity	Positive	Set Nebulizer	0.4 Bar
Focus	Active	Set Capillary	4500 V	Set Dry Heater	180 °C
Scan Begin	70 m/z	Set End Plate Offset	-500 V	Set Dry Gas	4.0 l/min
Scan End	1500 m/z	Set Charging Voltage	0 V	Set Divert Valve	Waste
		Set Corona	0 nA	Set APCI Heater	0 °C

Meas. m/z	#	Ion Formula	Score	m/z	err [ppm]	err [mDa]	mSigma	rdb	e⁻ Conf	N-Rule
489.2839	1	C28H41O7	100.00	489.2847	1.6	0.8	21.5	8.5	even	ok
511.2663	1	C28H40NaO7	100.00	511.2666	0.6	0.3	225.9	8.5	even	ok
977.5613	1	C56H81O14	100.00	977.5621	0.8	0.8	20.1	16.5	even	ok
999.5430	1	C56H80NaO14	100.00	999.5440	1.1	1.1	53.9	16.5	even	ok

Figure S43. The HRESIMS spectrum of compound 5.

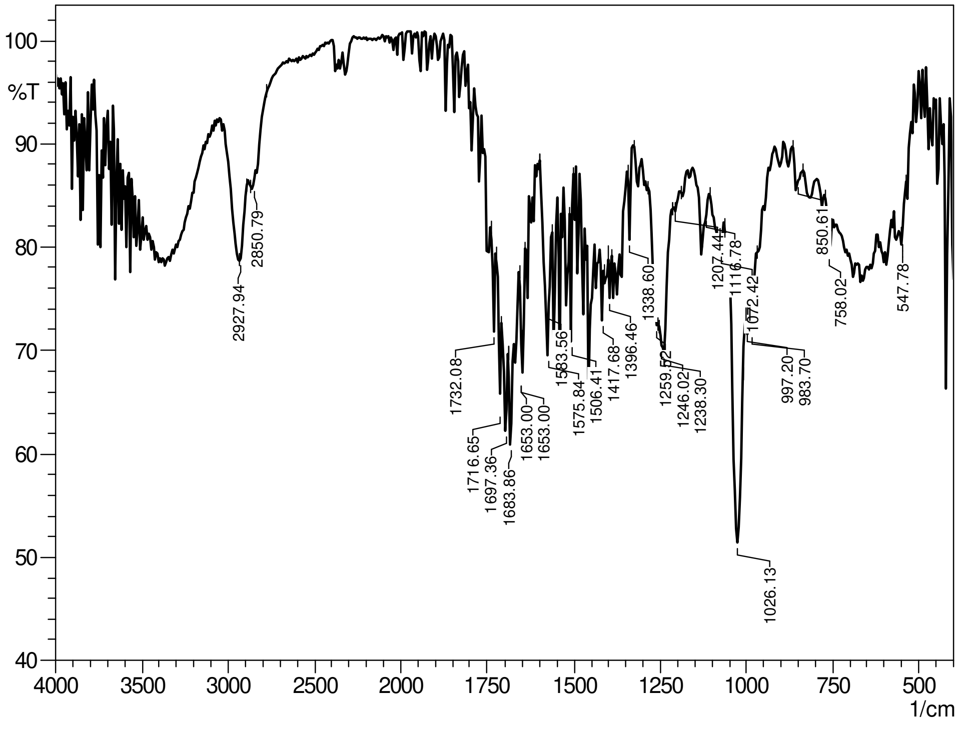

Figure S44. The IR spectrum of compound 5.

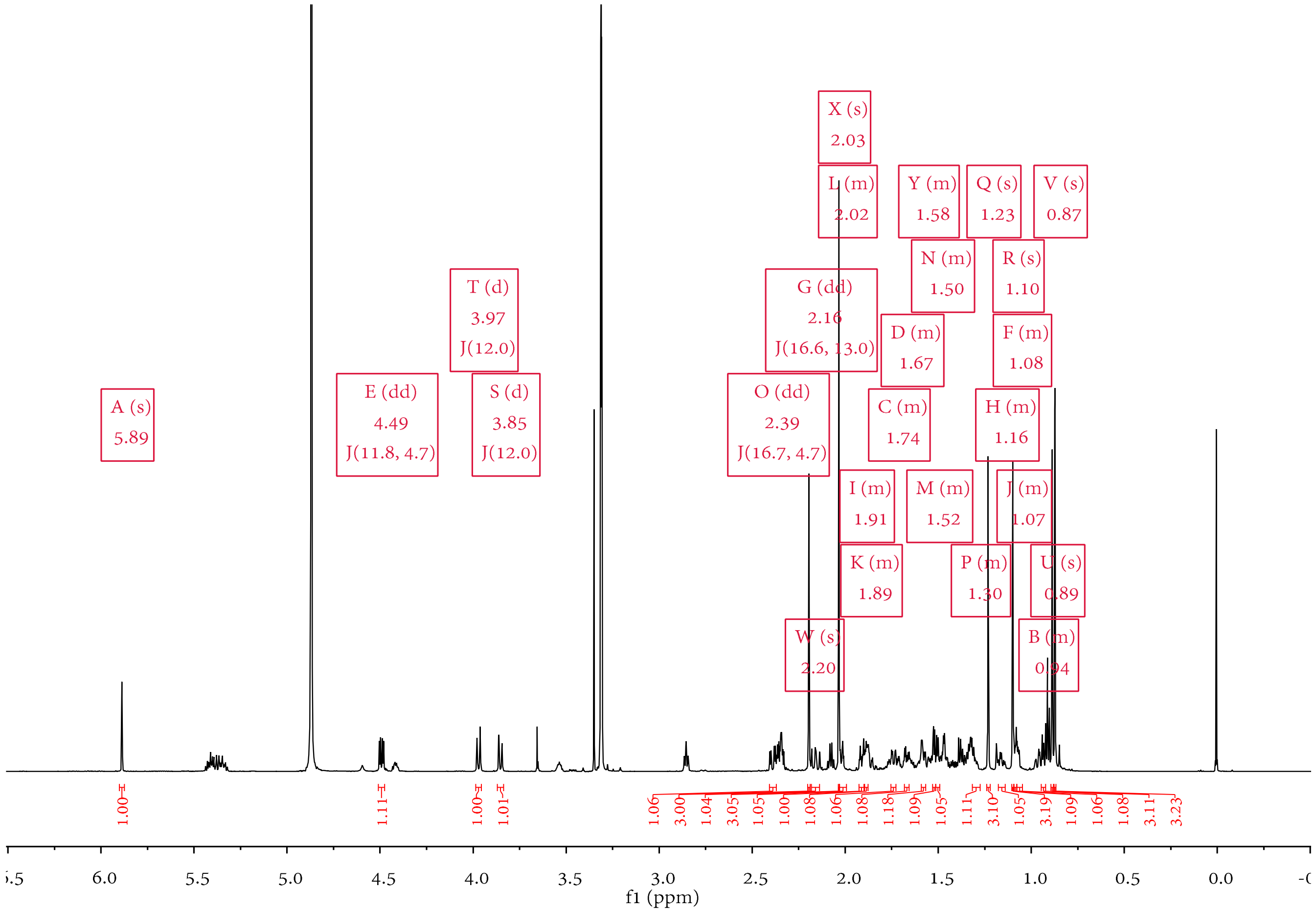

Figure S45. The ^{1}H NMR spectrum of compound 6 in CD_3OD.

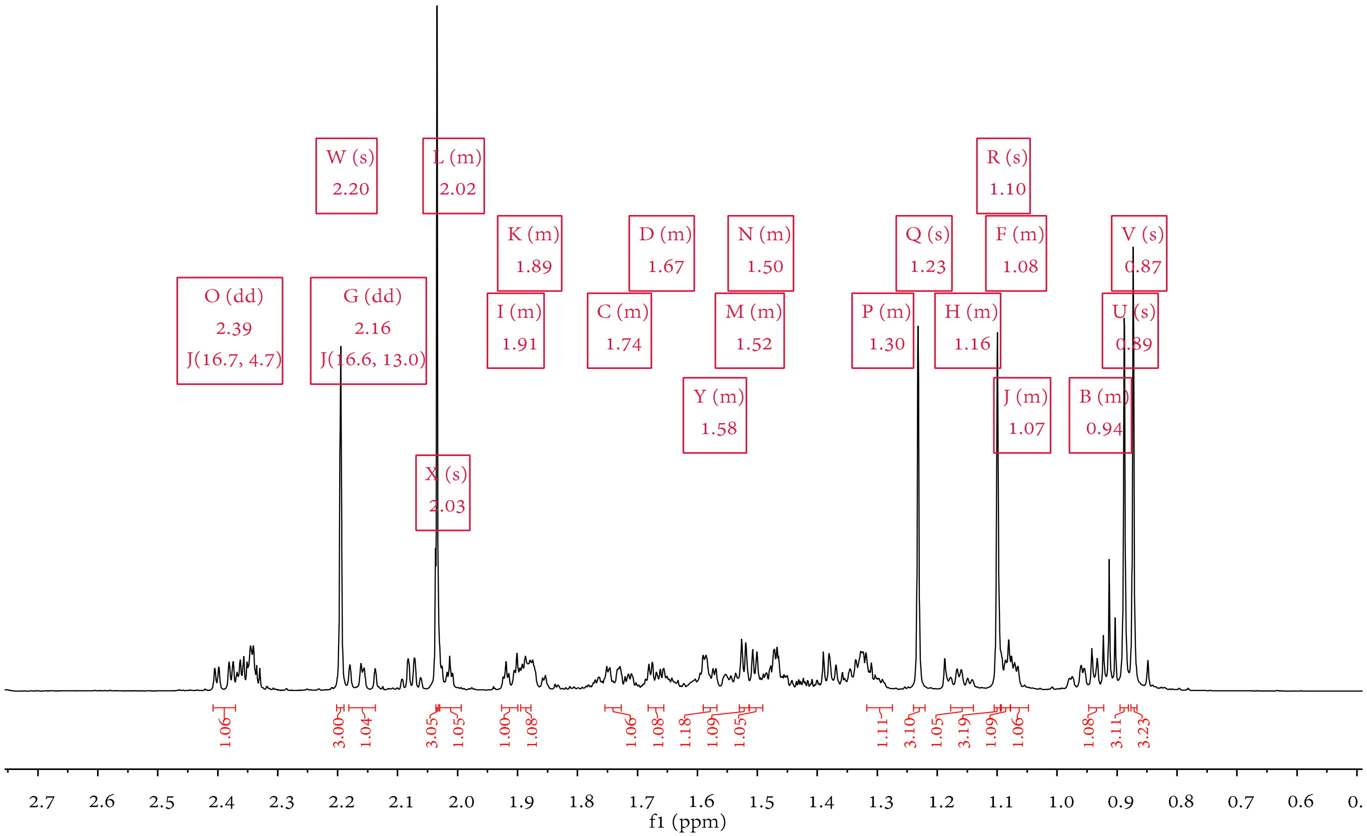

Figure S46. The ^{1}H NMR spectrum (0-3 ppm) of compound 6 in CD_3OD.

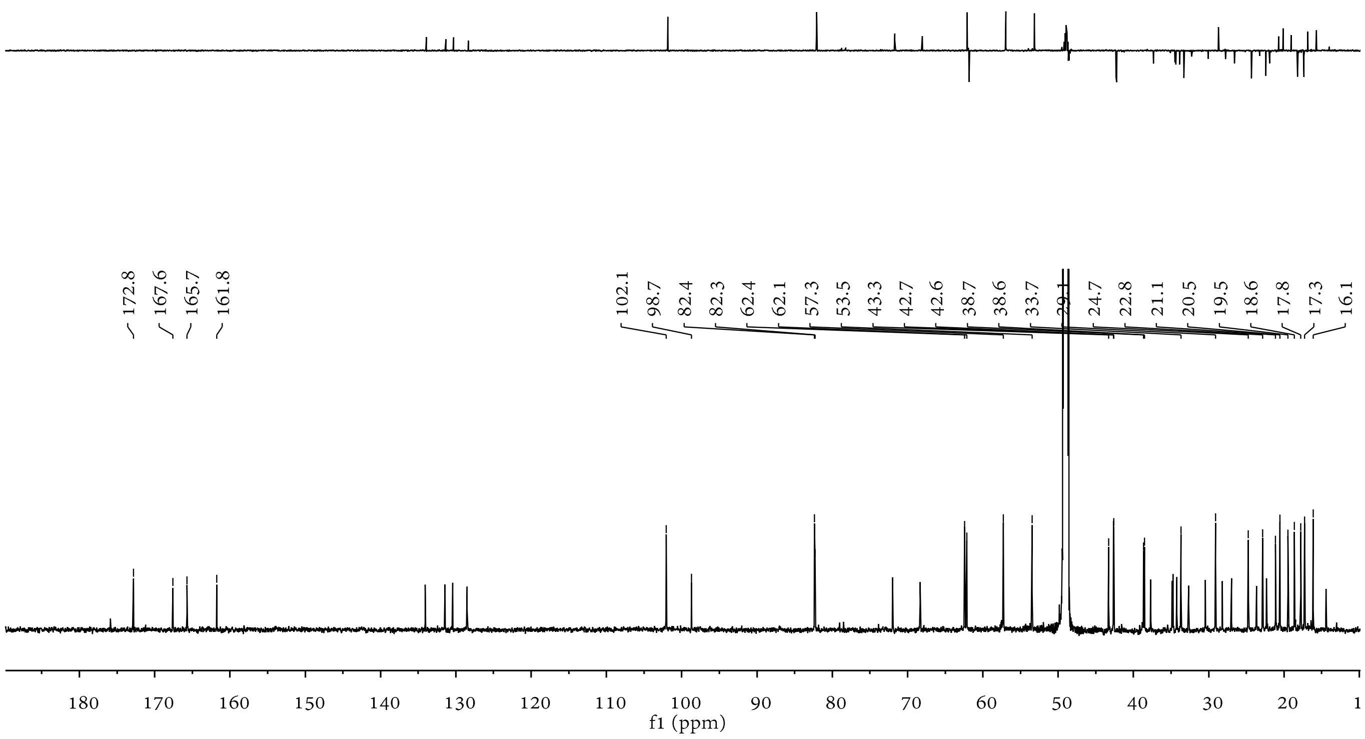

Figure S47. The ^{13}C NMR spectrum of compound 6 in CD_3OD.

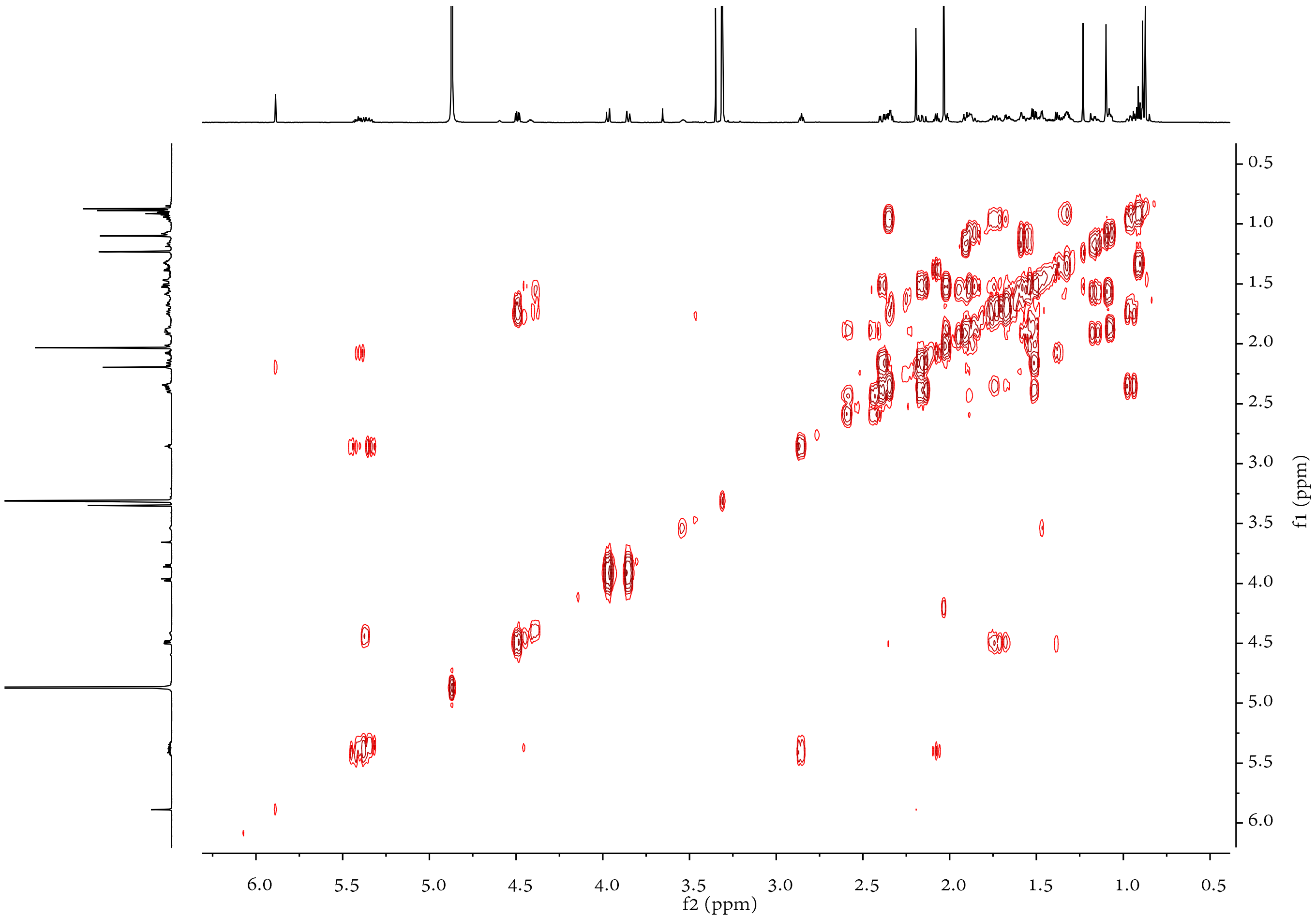

Figure S48. The ^1H-^1H COSY spectrum of compound 6 in CD_3OD.

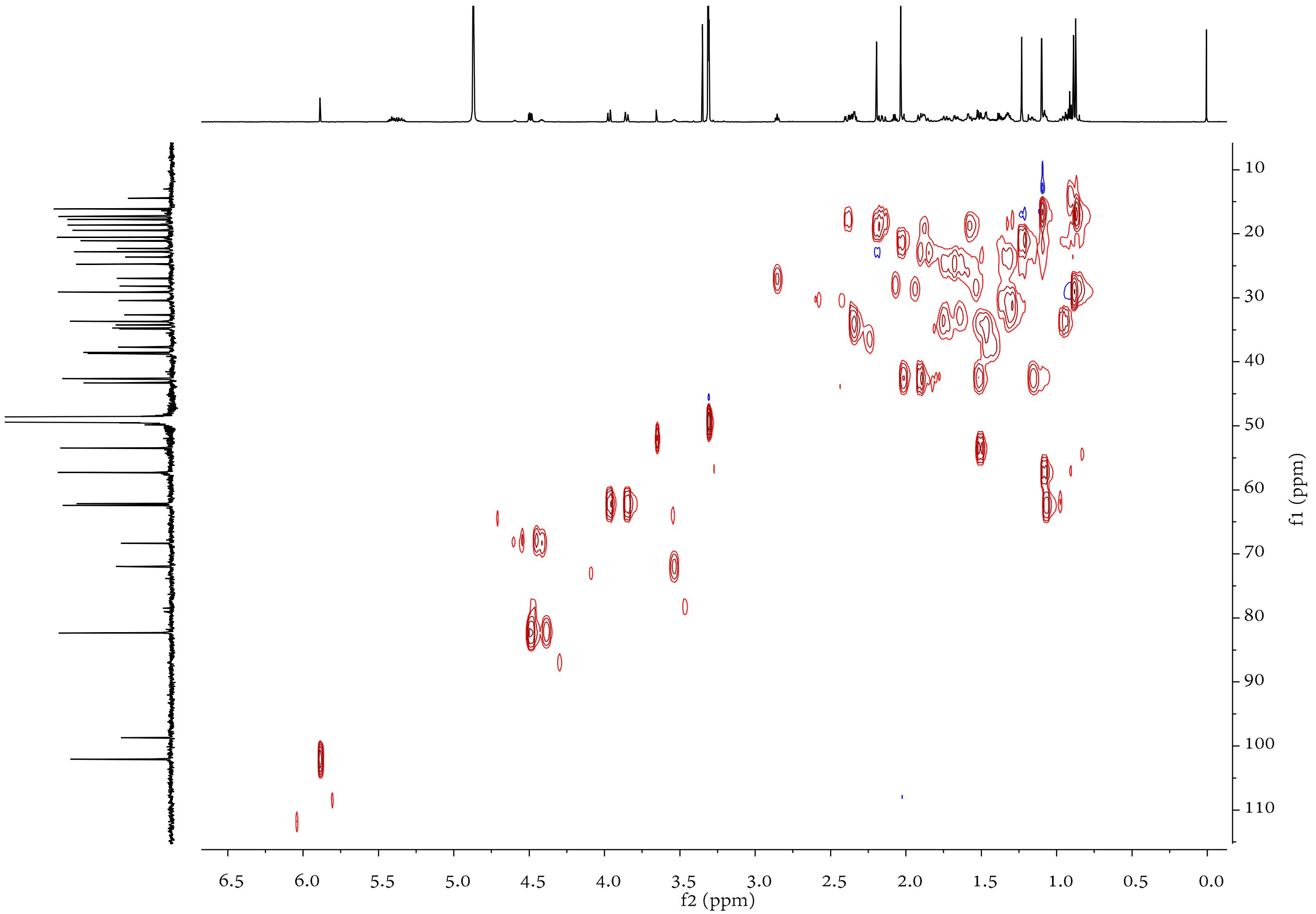

Figure S49. The HSQC spectrum of compound 6 in CD_3OD.

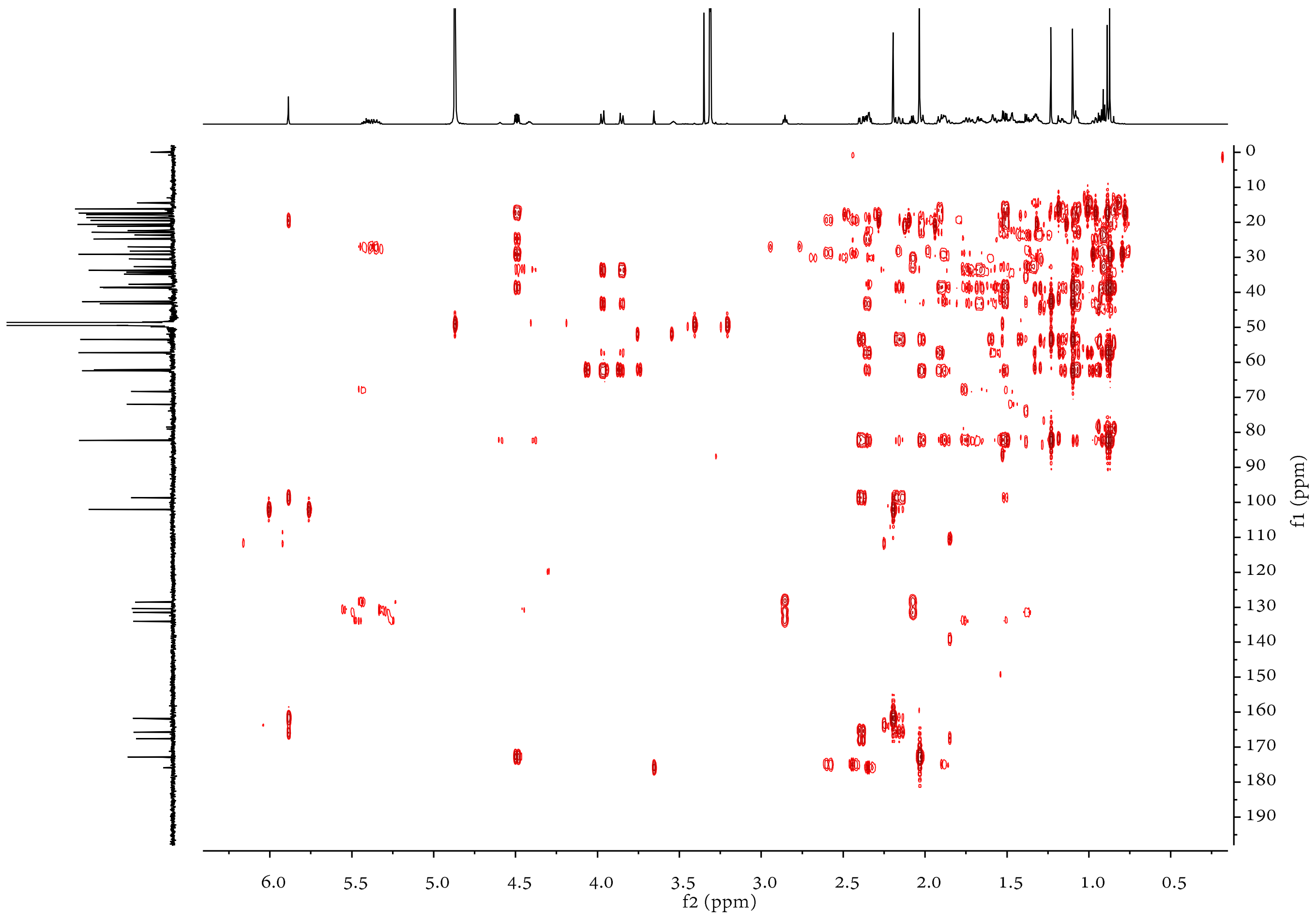

Figure S50. The HMBC spectrum of compound 6 in CD_3OD.

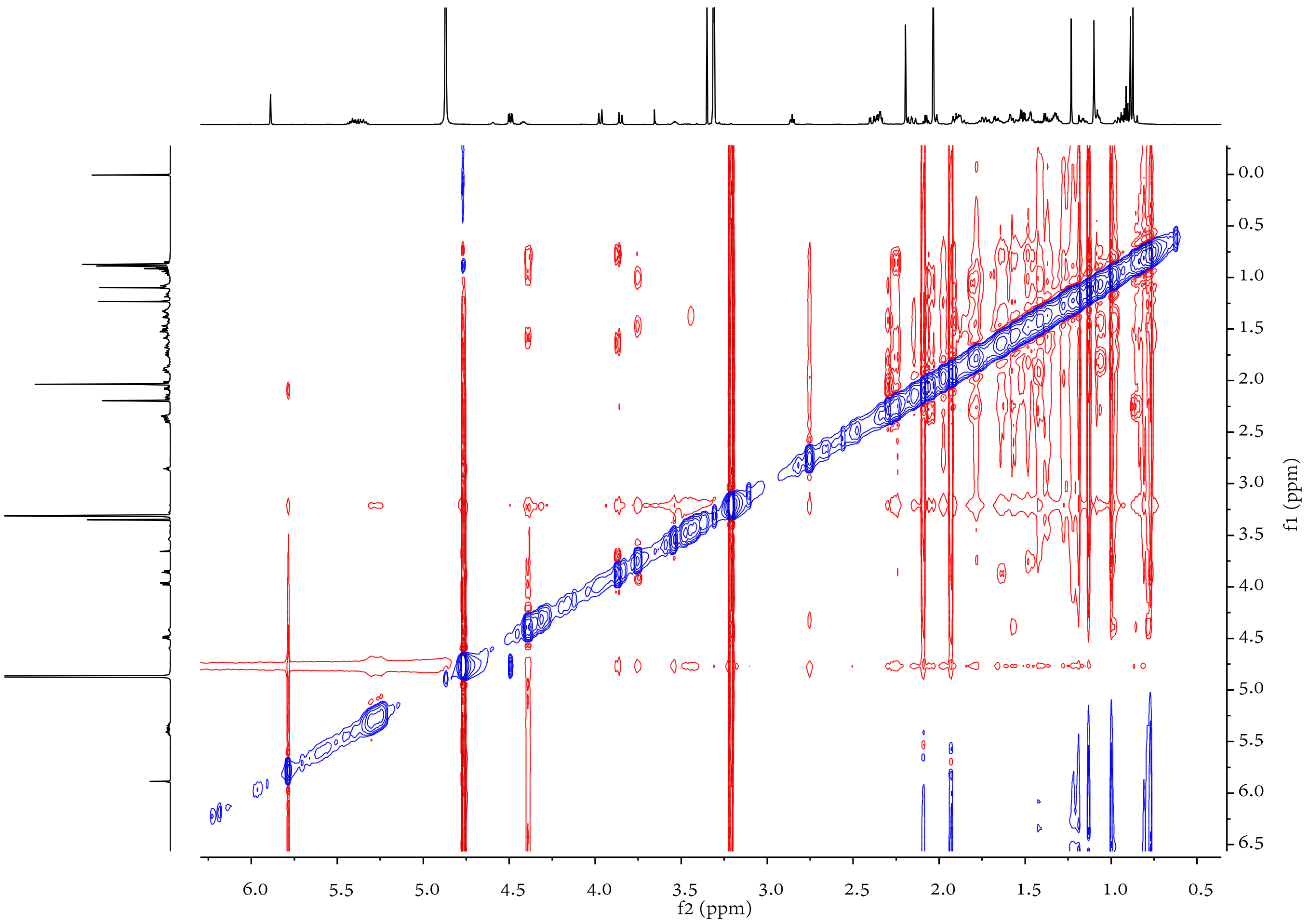

Figure S51. The NOESY spectrum of compound 6 in CD_3OD.

Mass Spectrum SmartFormula Report

Analysis Info

Analysis Name	D:\Data\MS\data\202005\chenxiayu_G8-b_pos_1_01_8292.d	Acquisition Date	5/29/2020 11:13:18 AM
Method	LC_Direct Infusion_pos_70-500mz.m	Operator	SCSIO
Sample Name	chenxiayu_G8-b_pos	Instrument	maXis 255552.00029
Comment			

Acquisition Parameter

Source Type	ESI	Ion Polarity	Positive	Set Nebulizer	0.4 Bar
Focus	Active	Set Capillary	4500 V	Set Dry Heater	180 °C
Scan Begin	70 m/z	Set End Plate Offset	-500 V	Set Dry Gas	4.0 l/min
Scan End	1500 m/z	Set Charging Voltage	0 V	Set Divert Valve	Waste
		Set Corona	0 nA	Set APCI Heater	0 °C

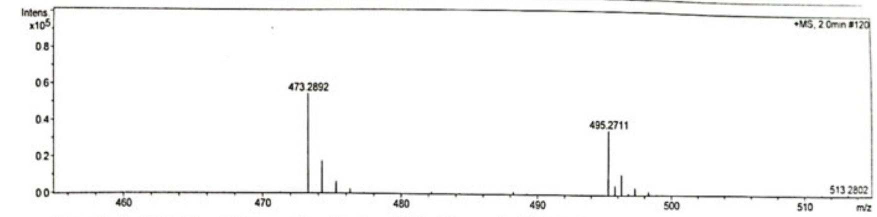

Meas. m/z	#	Ion Formula	Score	m/z	err [ppm]	err [mDa]	mSigma	rdb	e⁻ Conf	N-Rule
473.2892	1	C28H41O6	100.00	473.2898	-1.1	-0.5	34.1	8.5	even	ok
495.2711	1	C28H40NaO6	100.00	495.2717	1.3	0.6	30.7	8.5	even	ok
945.5701	1	C56H81O12	100.00	945.5723	2.3	2.1	99.4	16.5	even	ok
967.5526	1	C56H80NaO12	100.00	967.5542	1.6	1.6	88.6	16.5	even	ok

Figure S52. The HRESIMS spectrum of compound 6.

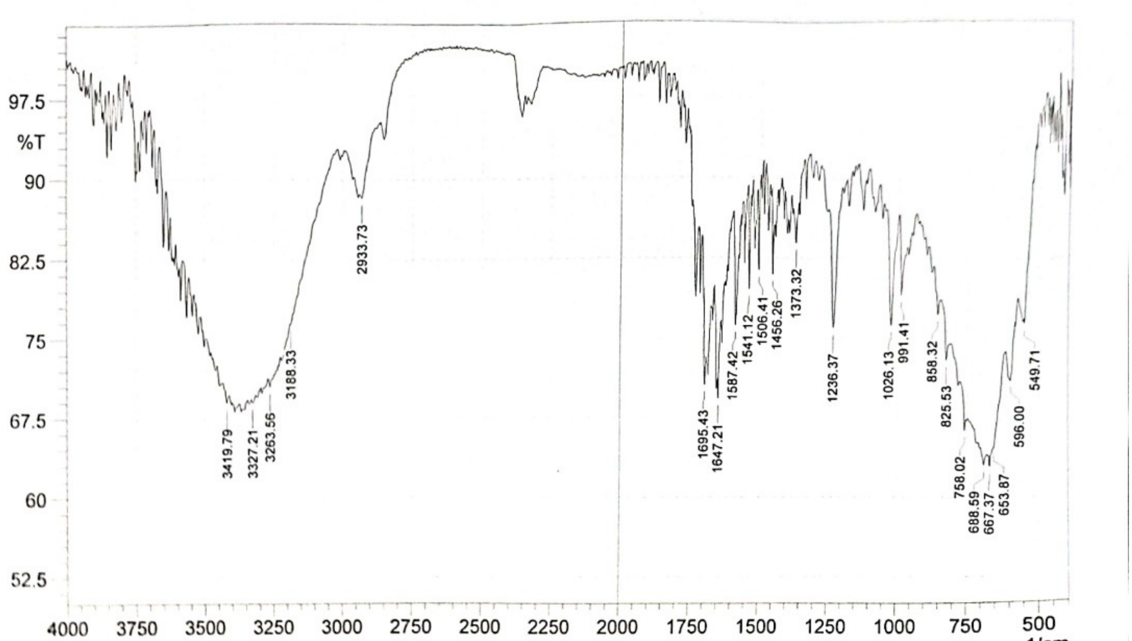

Figure S53. The IR spectrum of compound 6.

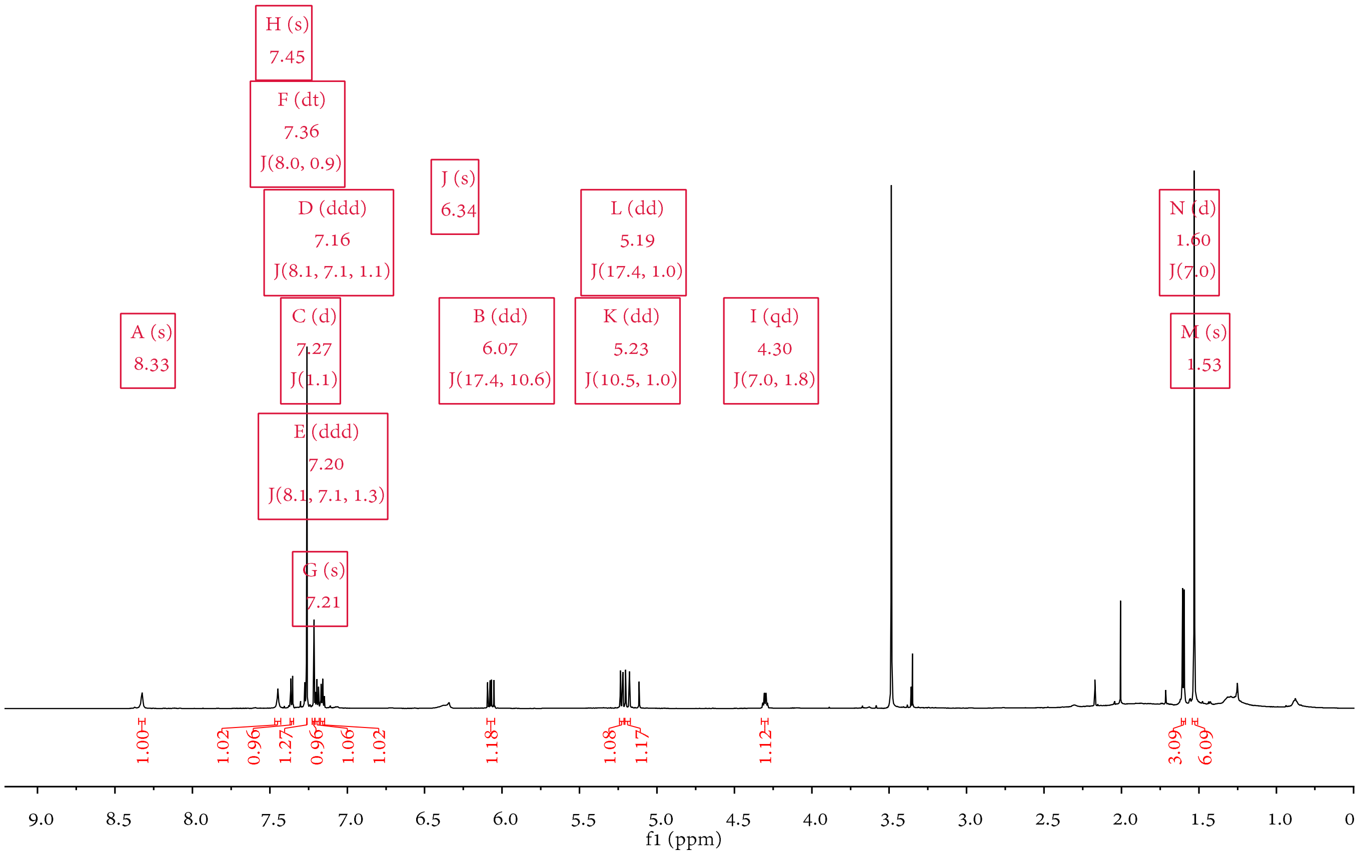

Figure S54. The ^{1}H NMR spectrum of compound 7 in $CDCl_3$.

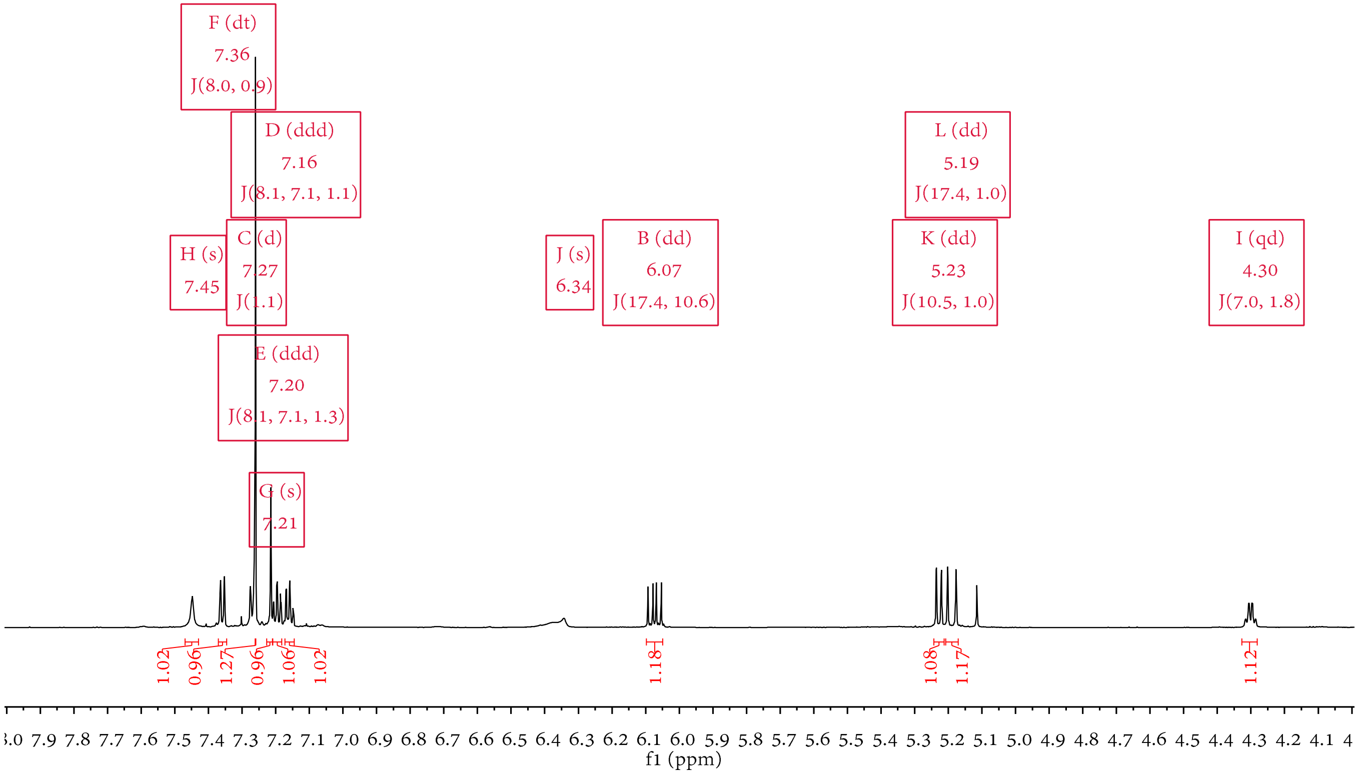

Figure S55. The ^{1}H NMR spectrum (4-8 ppm) of compound 7 in $CDCl_3$.

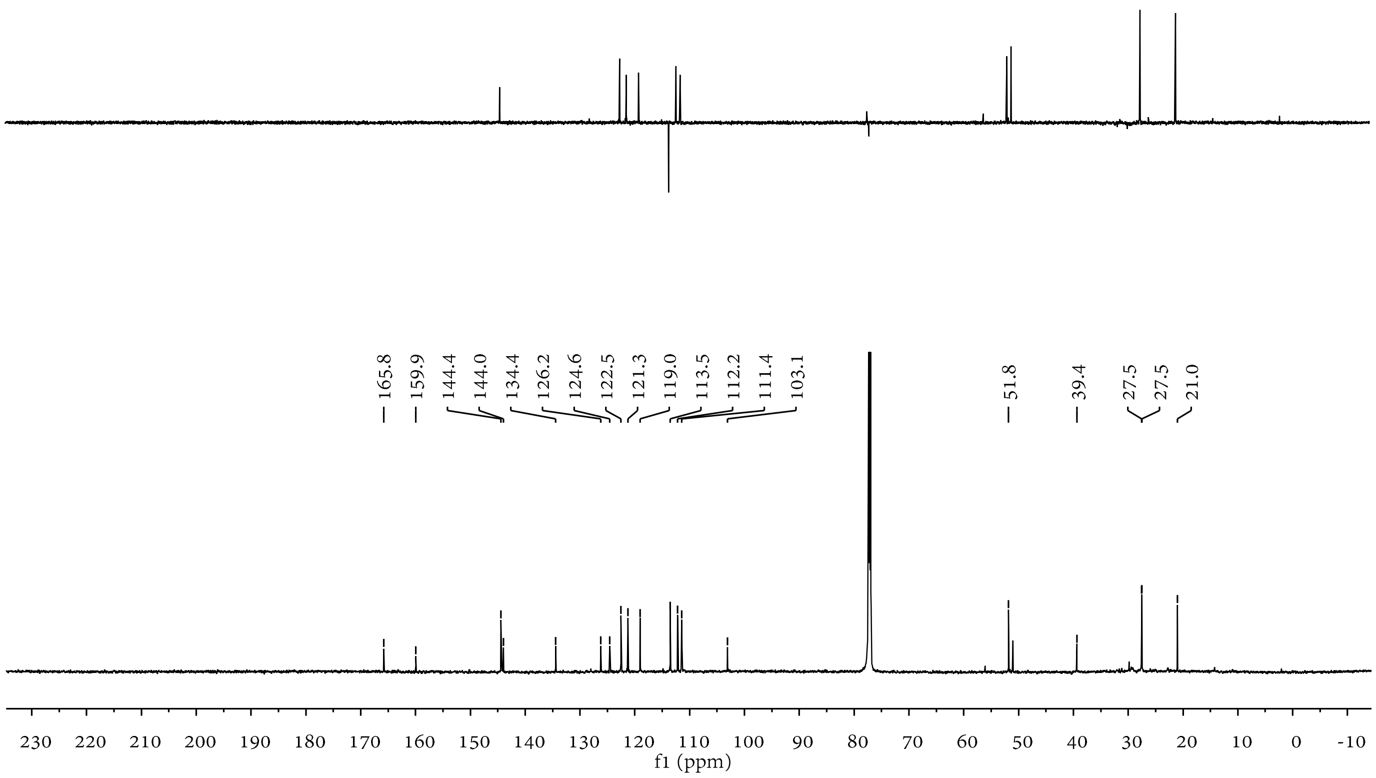

Figure S56. The ^{13}C NMR spectrum of compound 7 in $CDCl_3$.

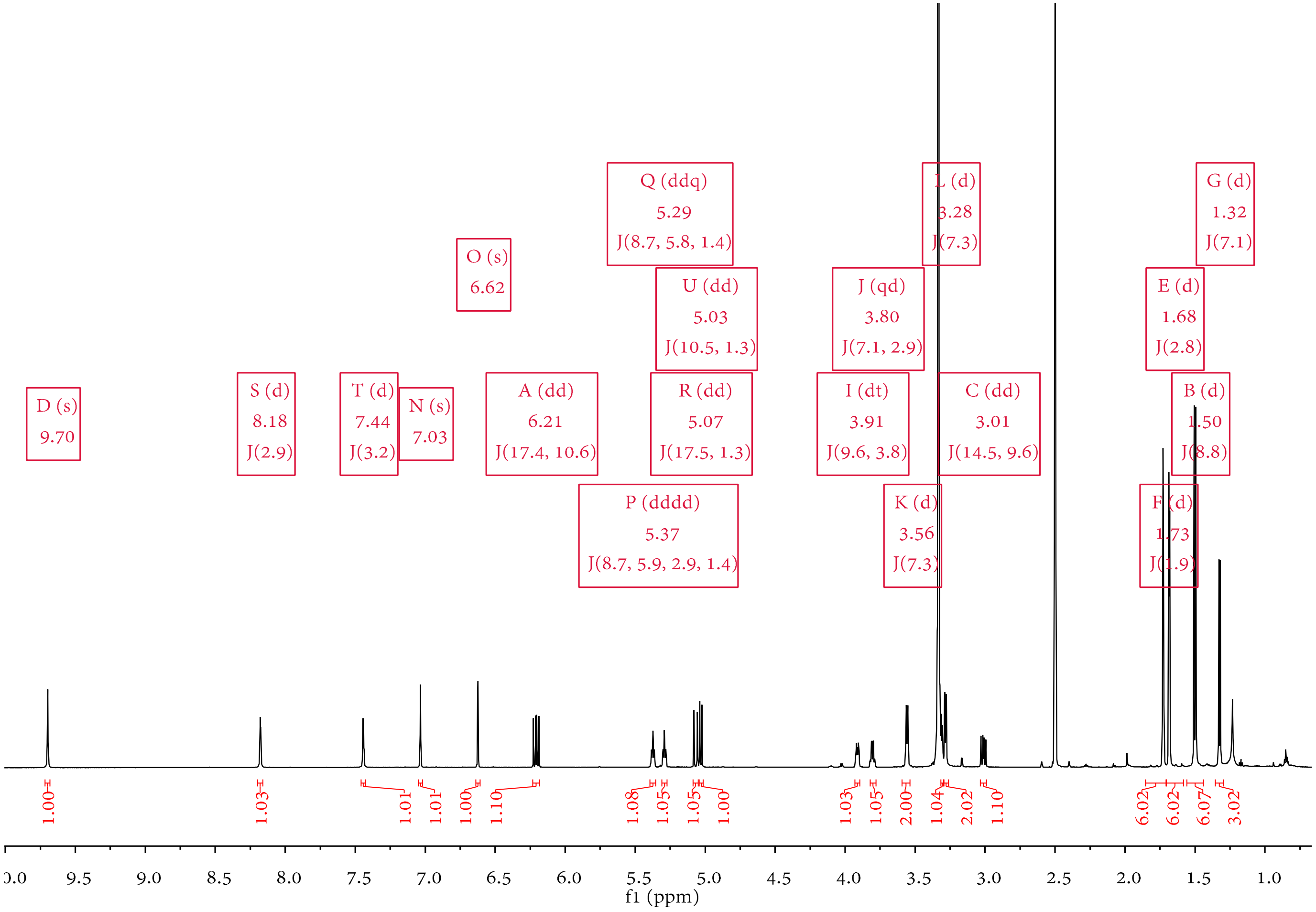

Figure S57. The ^{1}H NMR spectrum of compound 8 in DMSO-*d*₆.

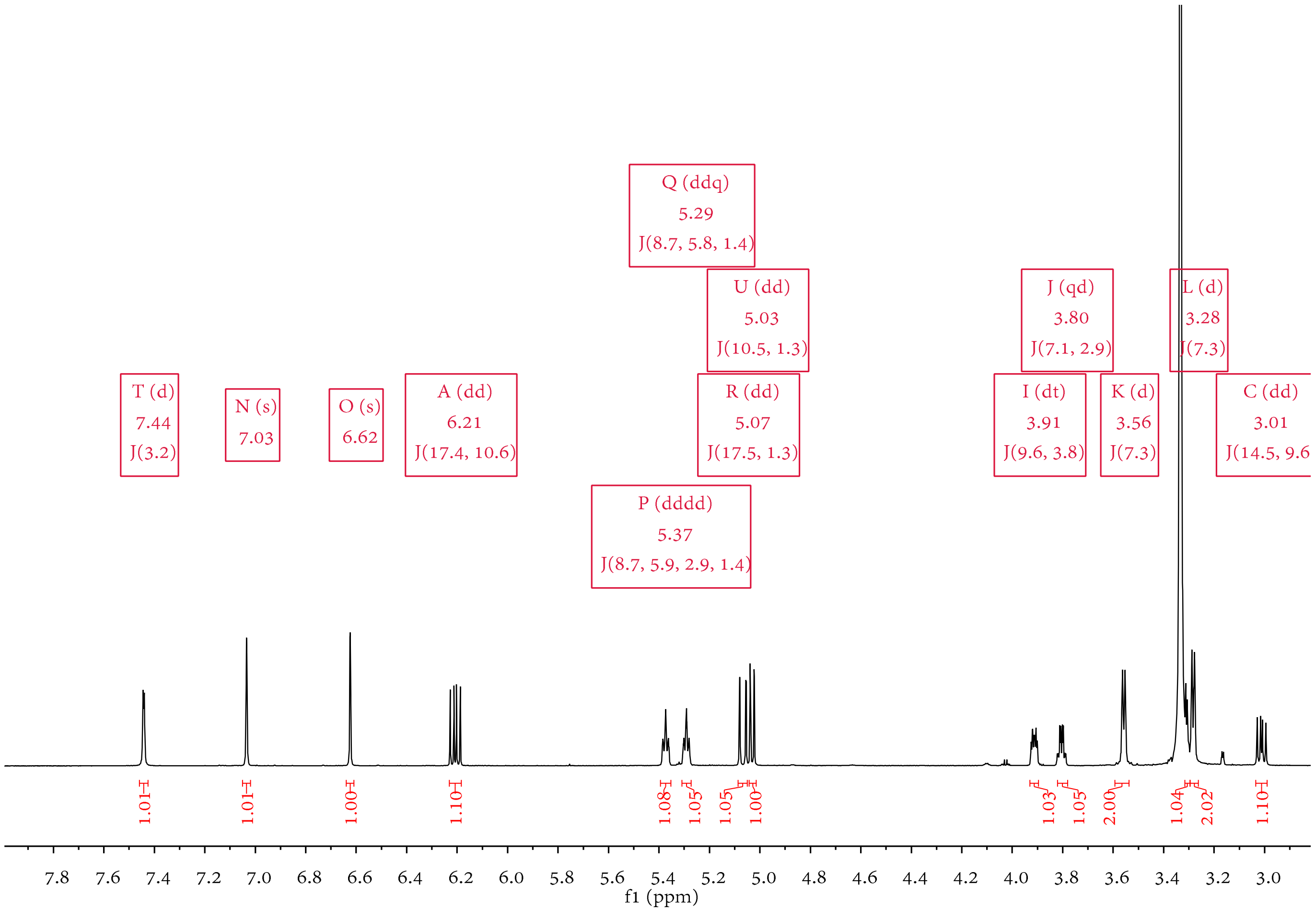

Figure S58. The ^{1}H NMR spectrum (3-8 ppm) of compound 8 in DMSO-d_6.

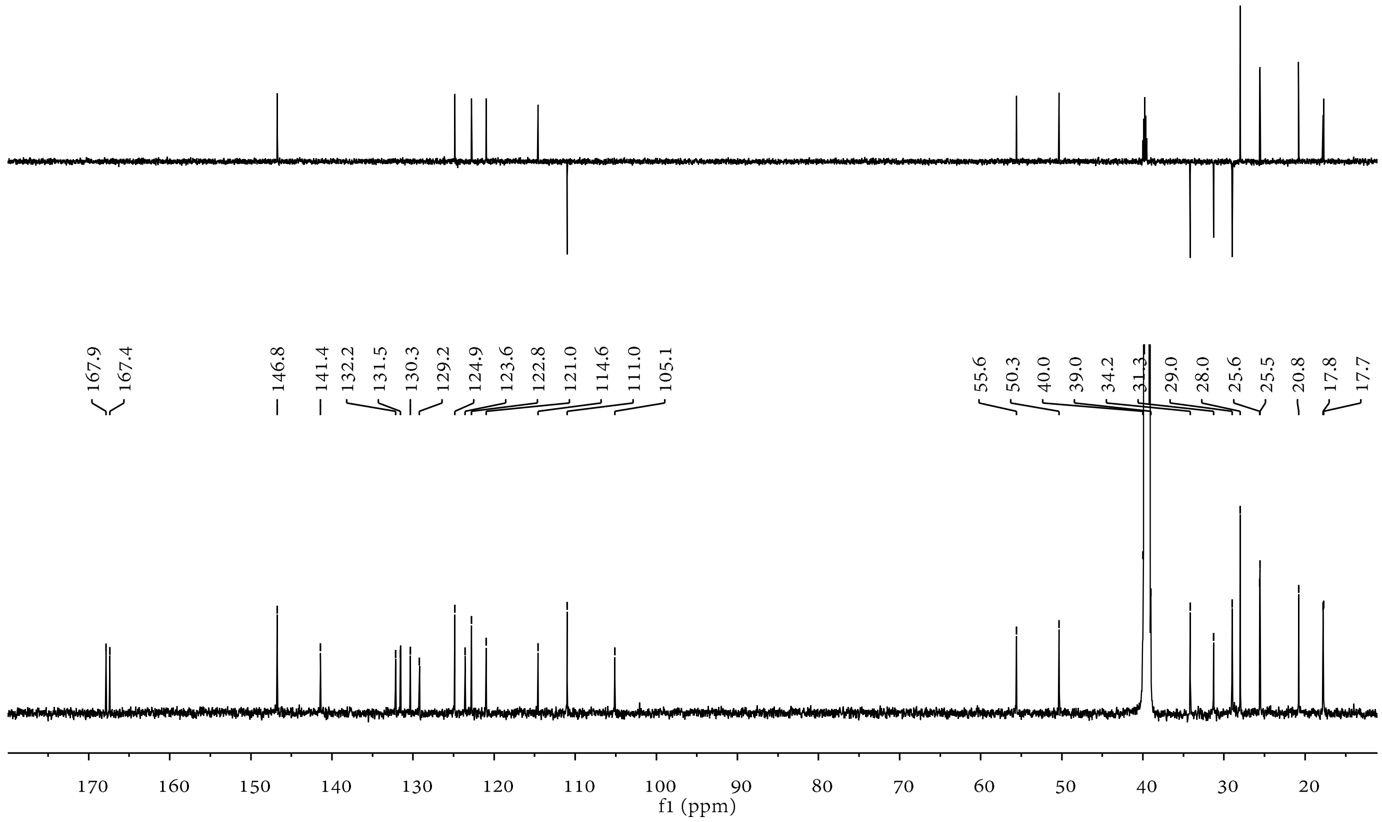

Figure S59. The ^{13}C NMR spectrum of compound 8 in DMSO-d_6.

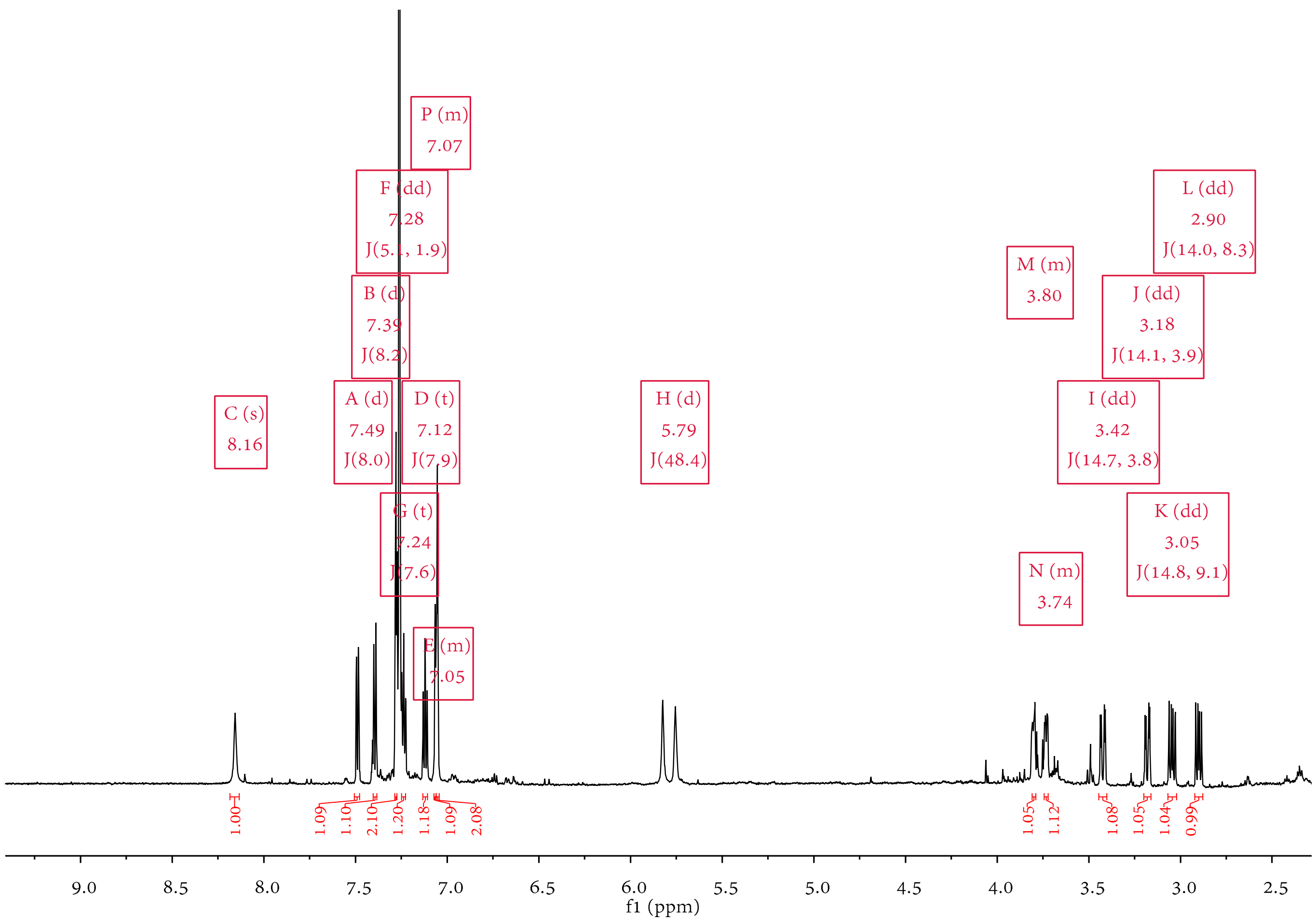

Figure S60. The ^{1}H NMR spectrum of compound 9 in $CDCl_3$.

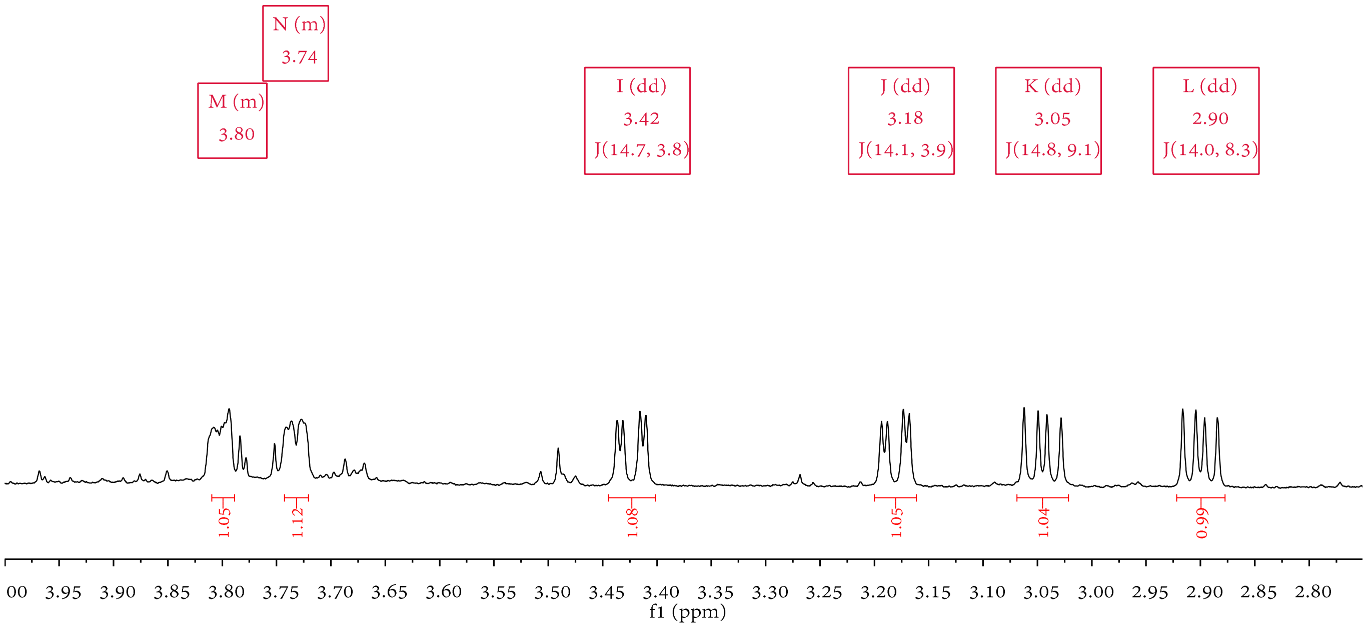

Figure S61. The ^{1}H NMR spectrum (2.8-4 ppm) of compound 9 in $CDCl_3$.

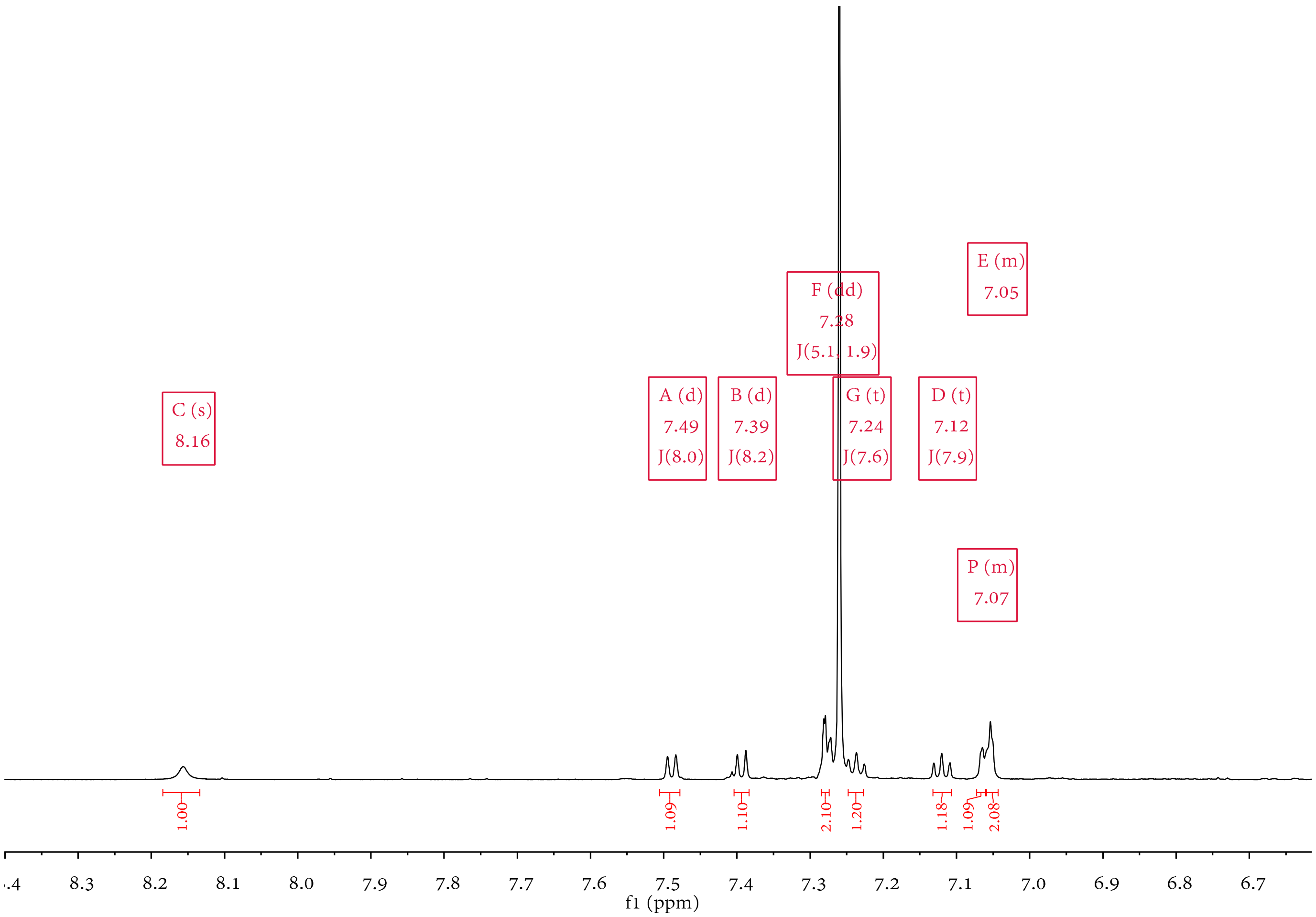

Figure S62. The ^{1}H NMR spectrum (6-8 ppm) of compound 9 in $CDCl_3$.

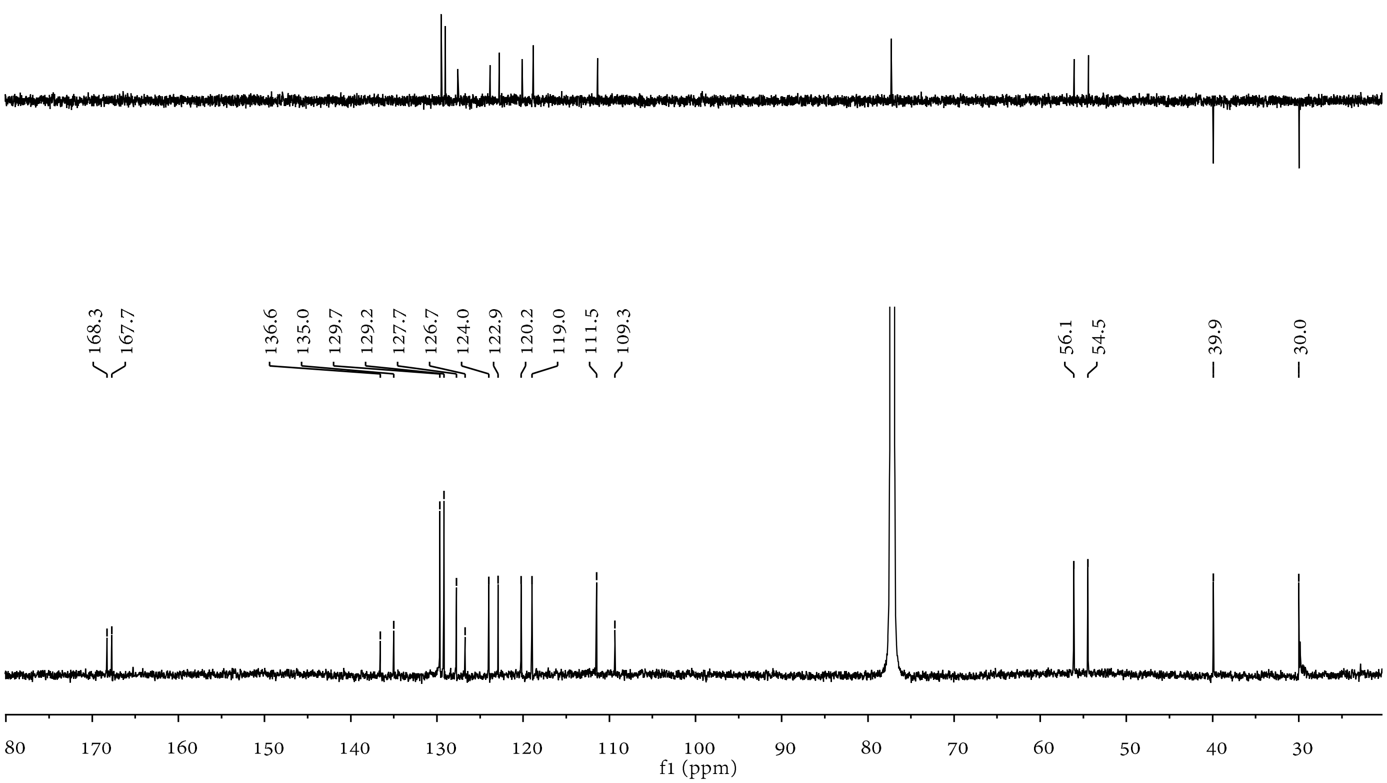

Figure S63. The ^{13}C NMR spectrum of compound 9 in $CDCl_3$.

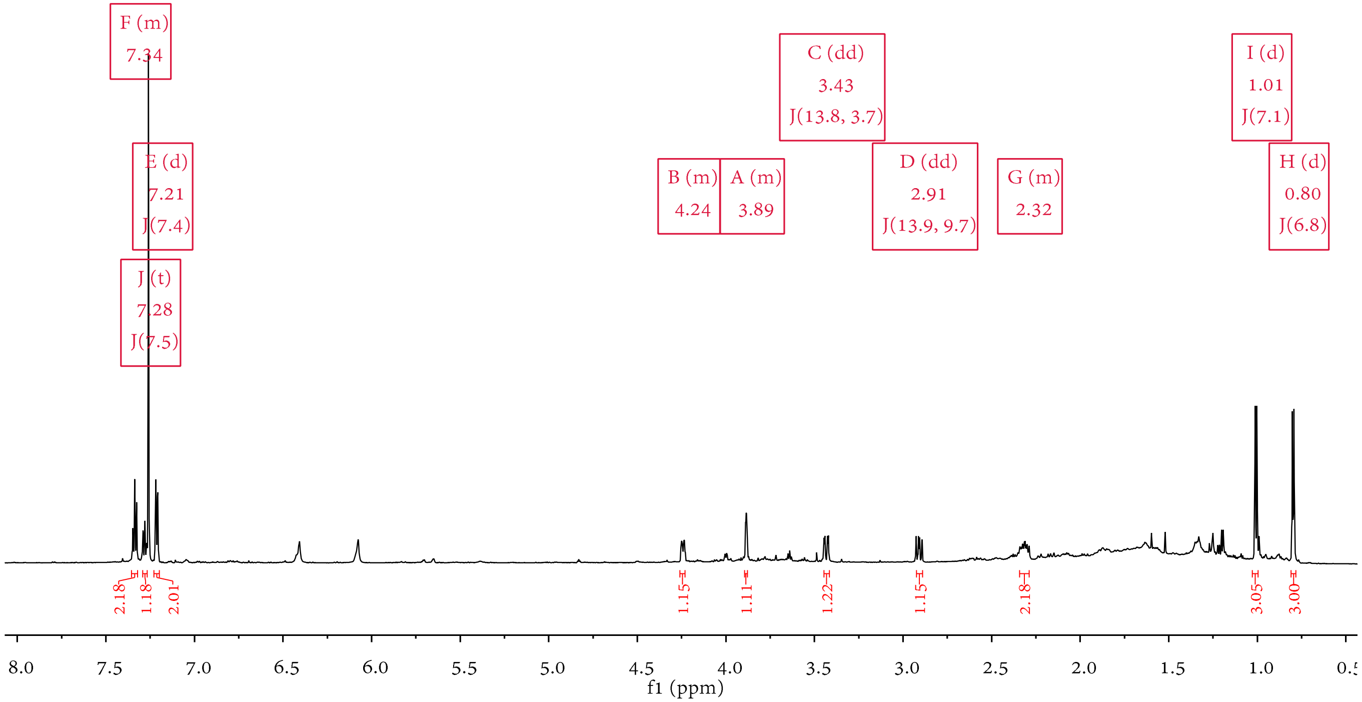

Figure S64. The ^{1}H NMR spectrum of compound 10 in $CDCl_3$.

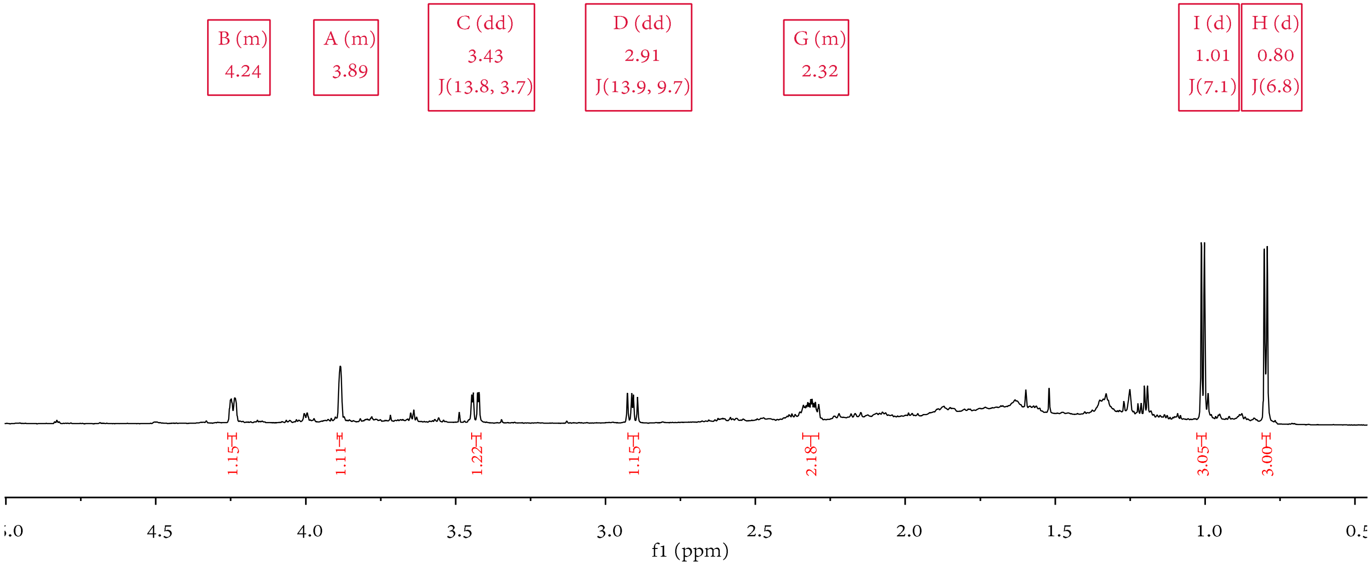

Figure S65. The ^{1}H NMR spectrum (0-5 ppm) of compound 10 in $CDCl_3$.

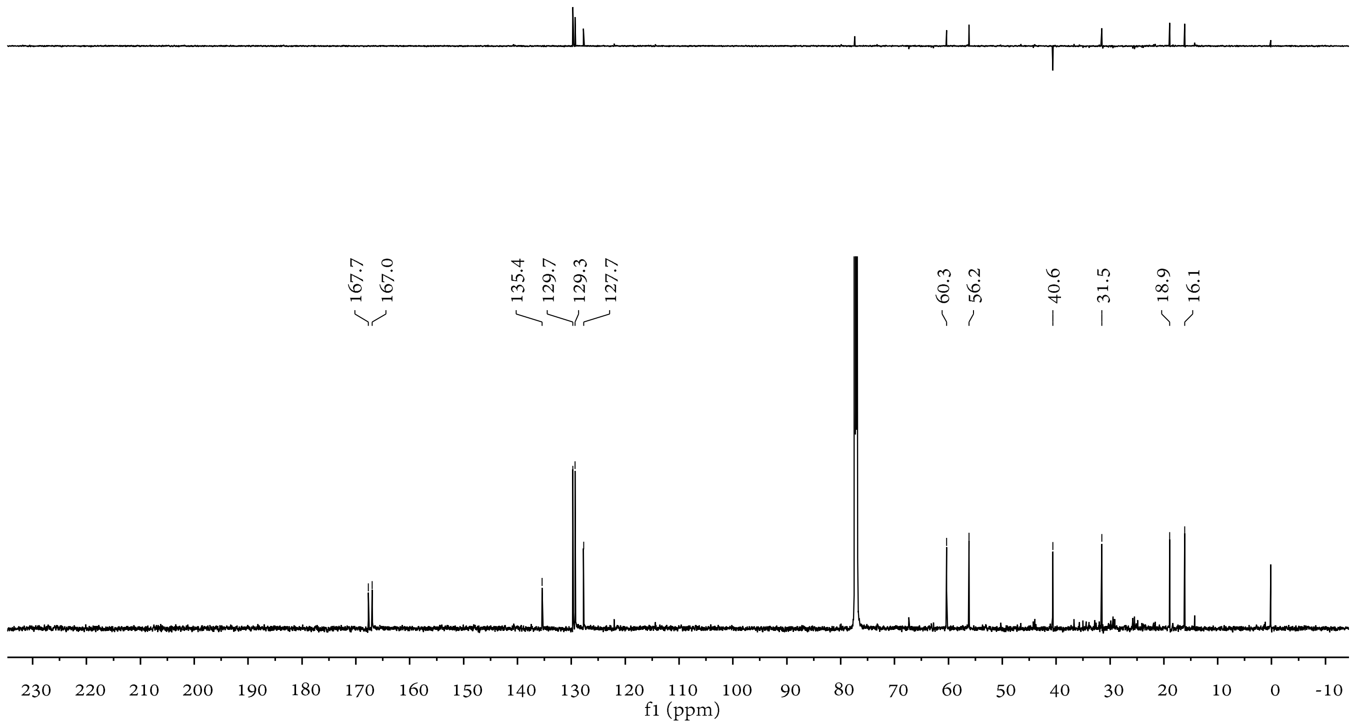

Figure S66. The ^{13}C NMR spectrum of compound 10 in $CDCl_3$.

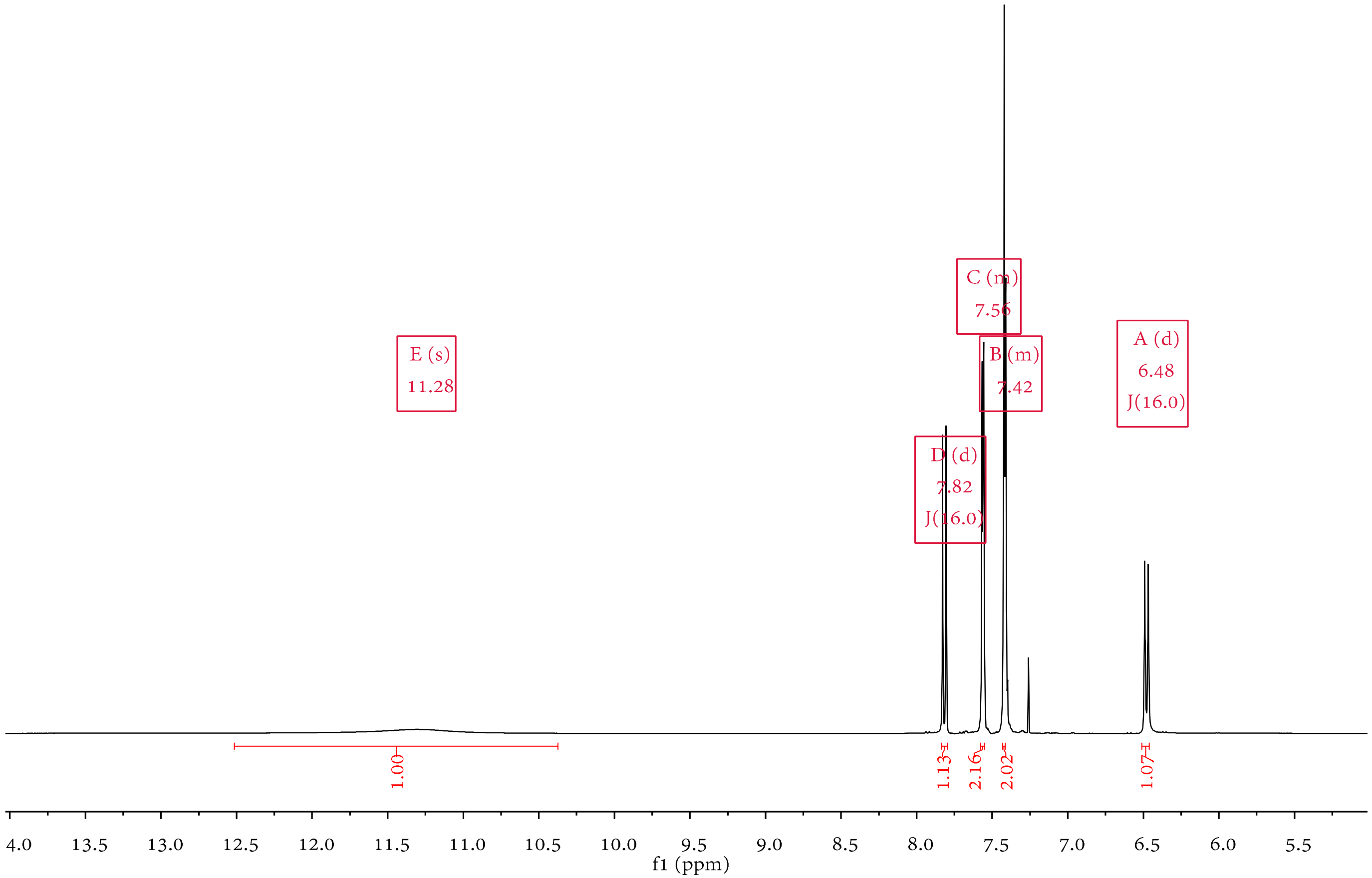

Figure S67. The ^{1}H NMR spectrum of compound 11 in $CDCl_3$.

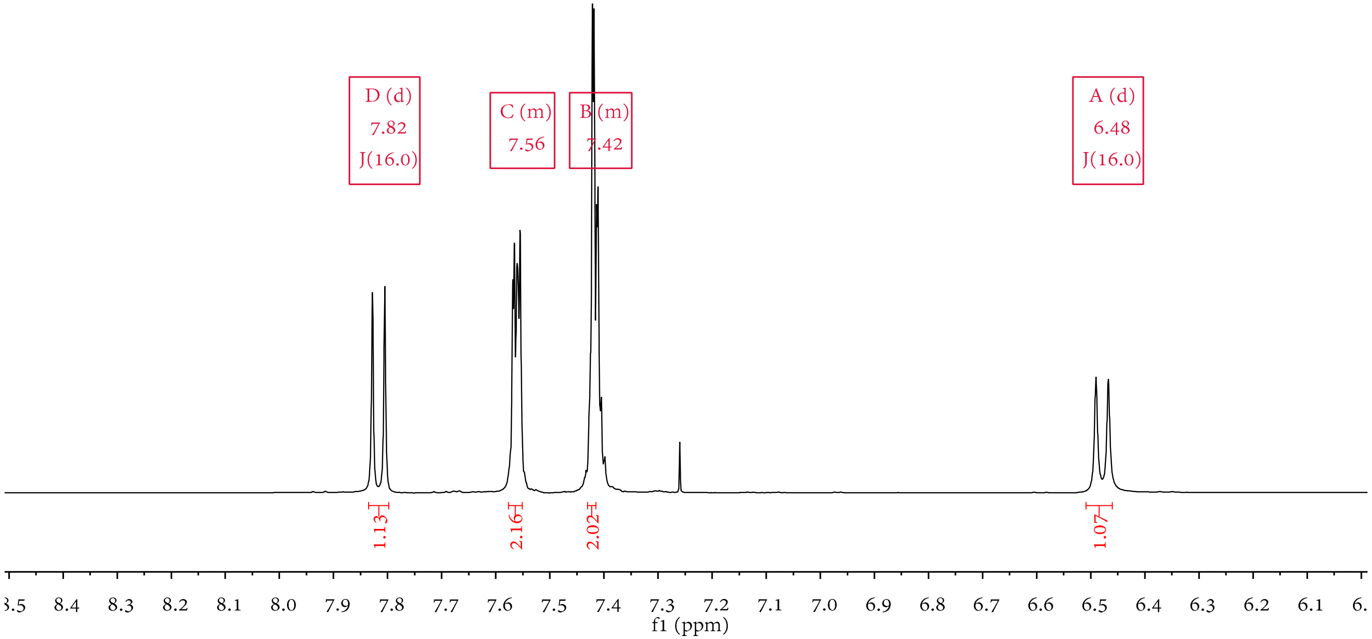

Figure S68. The ^{1}H NMR spectrum (6-8 ppm) of compound 11 in $CDCl_3$.

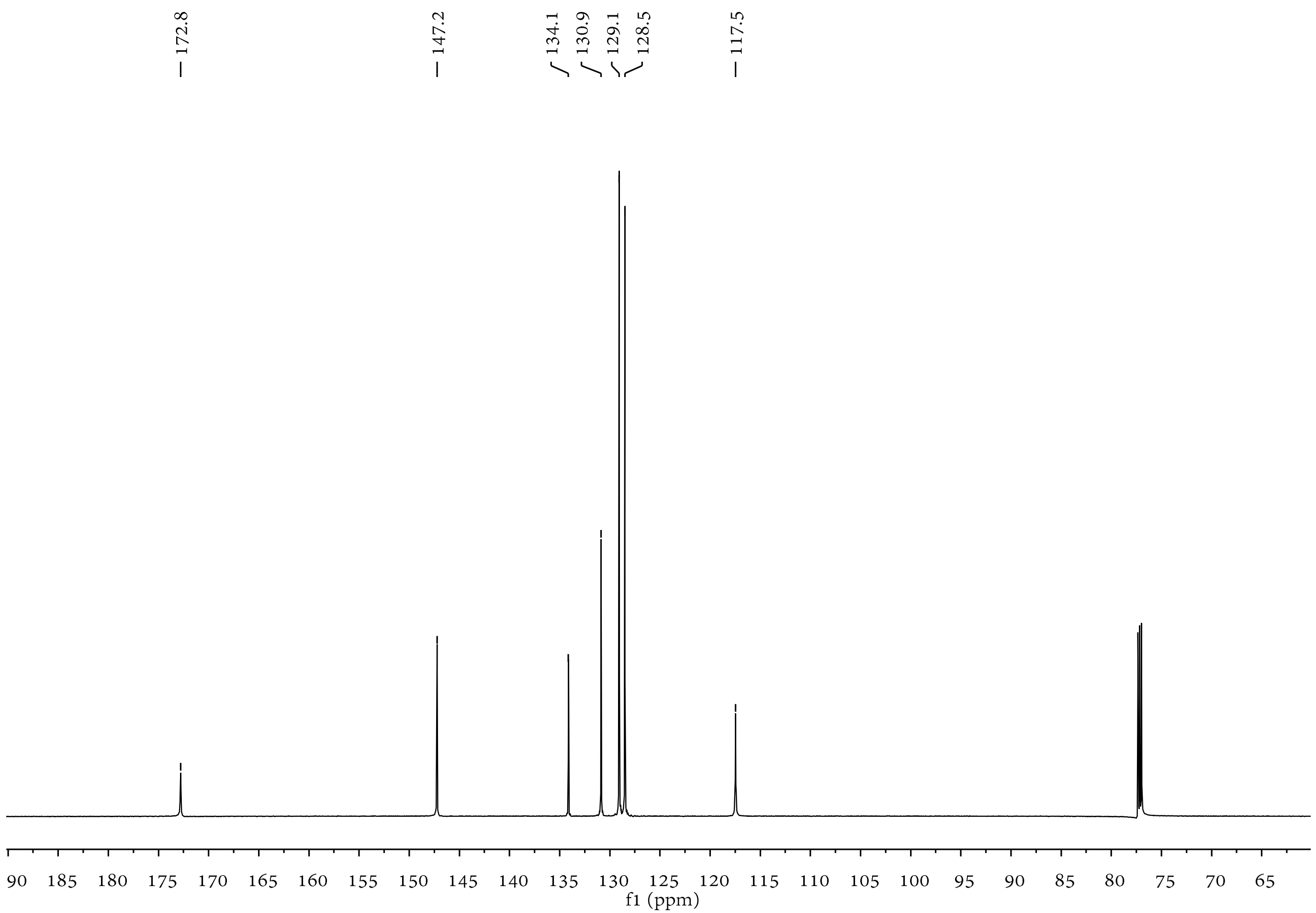

Figure S69. The ^{13}C NMR spectrum of compound 11 in $CDCl_3$.

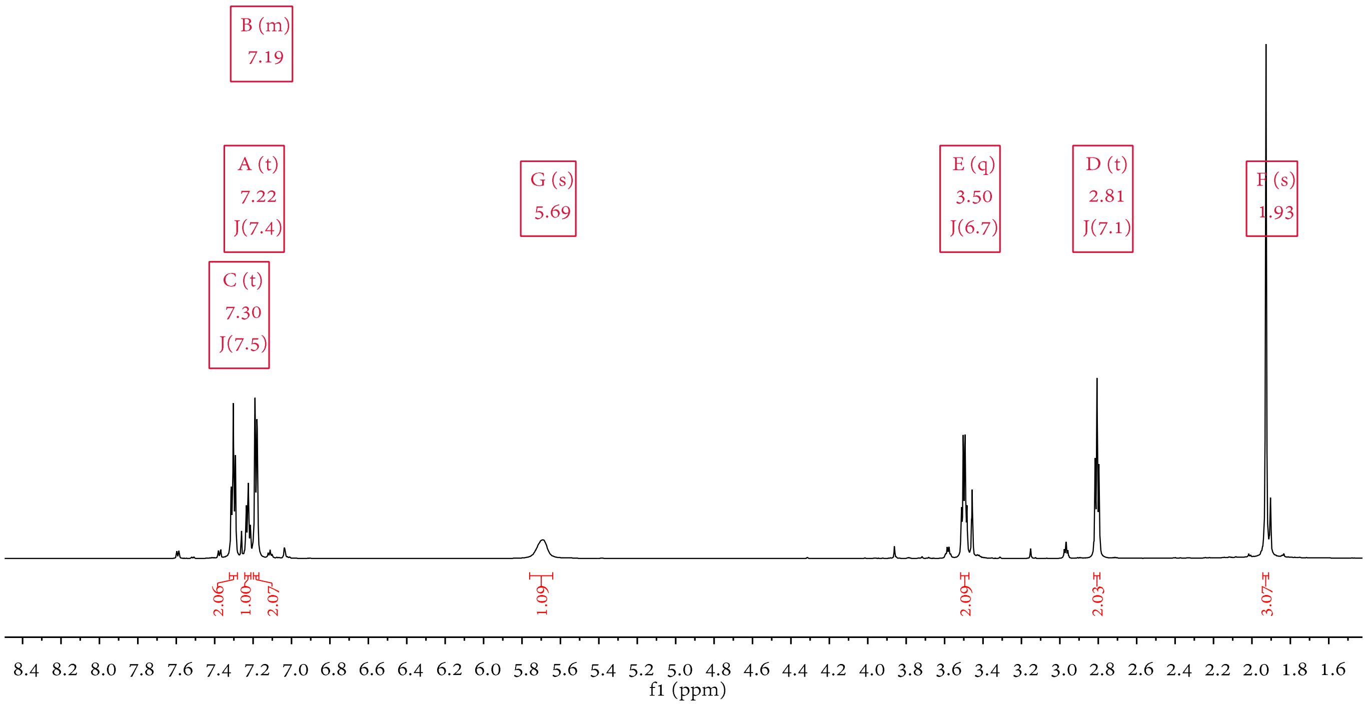

Figure S70. The ^{1}H NMR spectrum of compound 12 in $CDCl_3$.

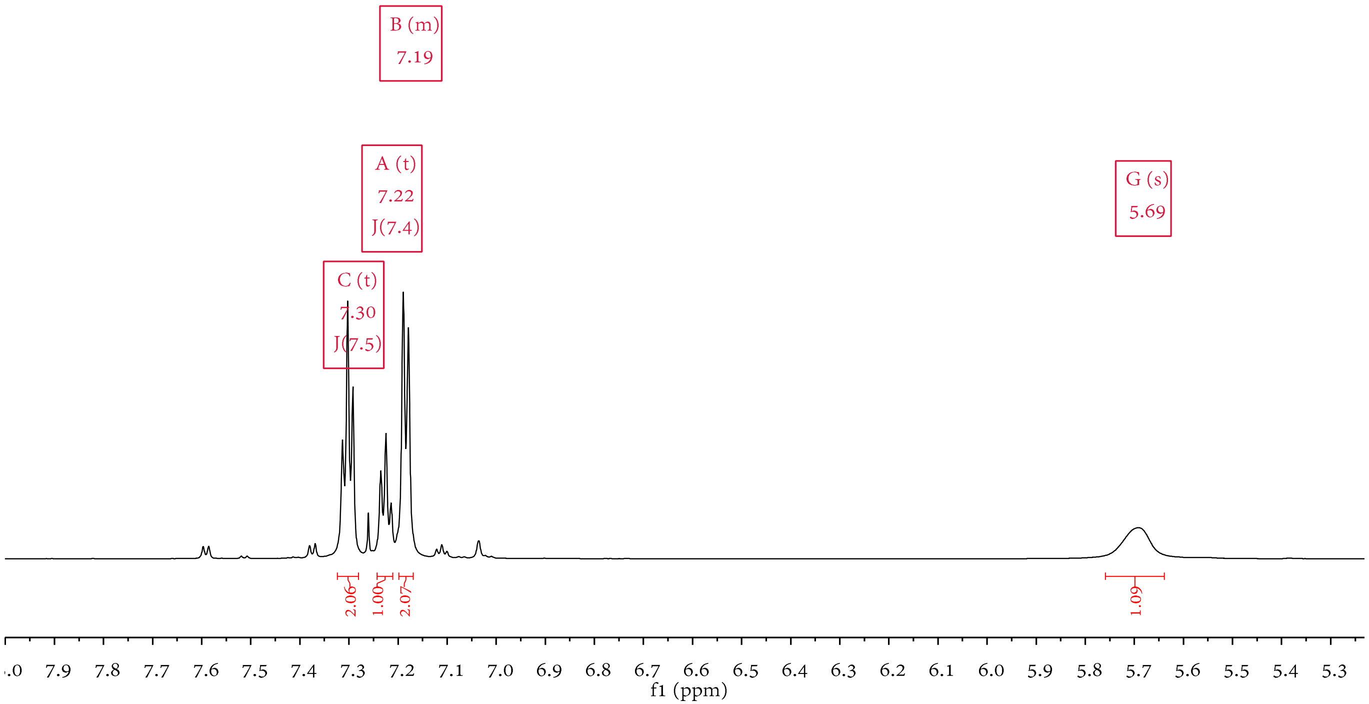

Figure S71. The ^{1}H NMR spectrum (5-8 ppm) of compound 12 in $CDCl_3$.

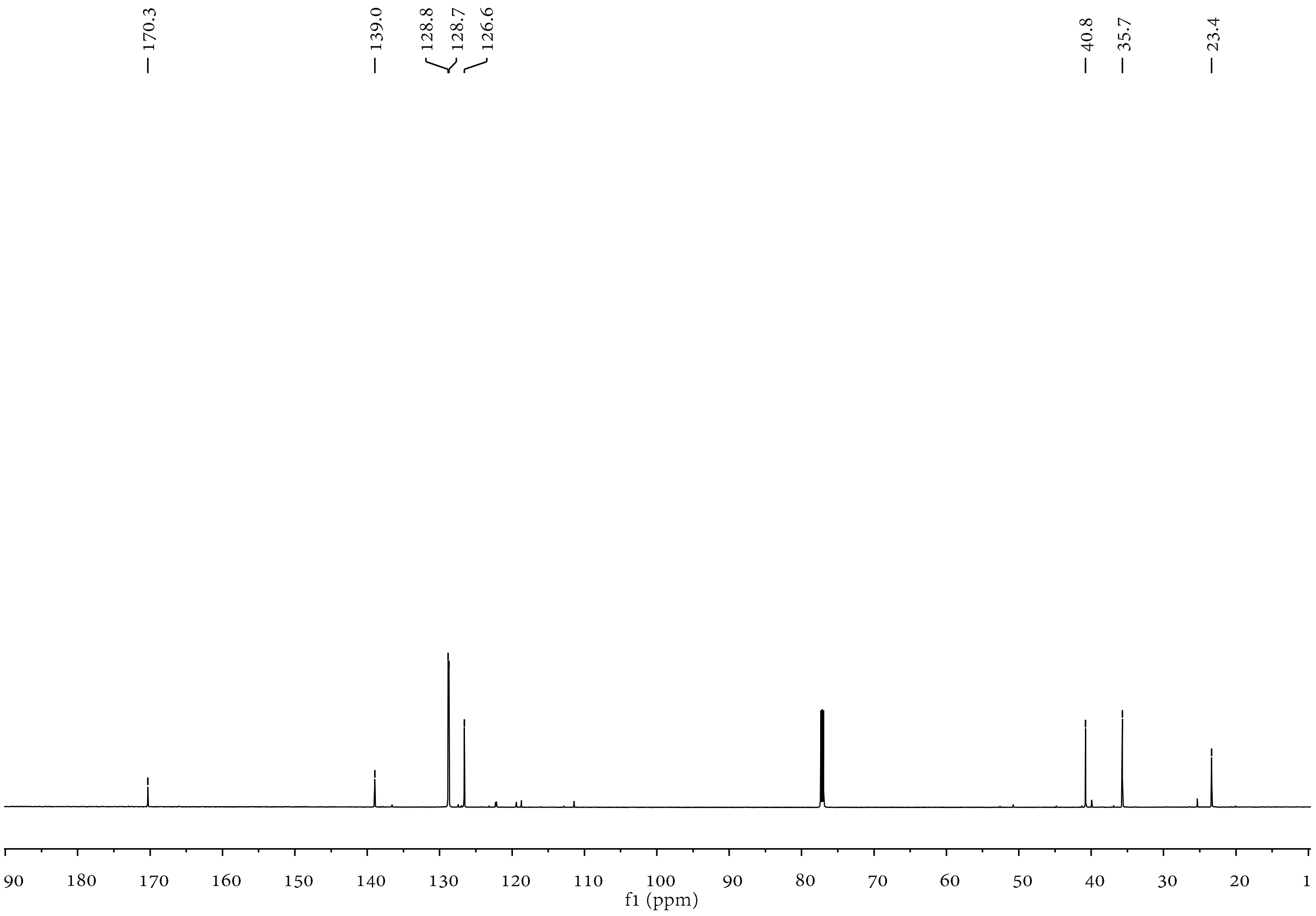

Figure S72. The ^{13}C NMR spectrum of compound 12 in $CDCl_3$

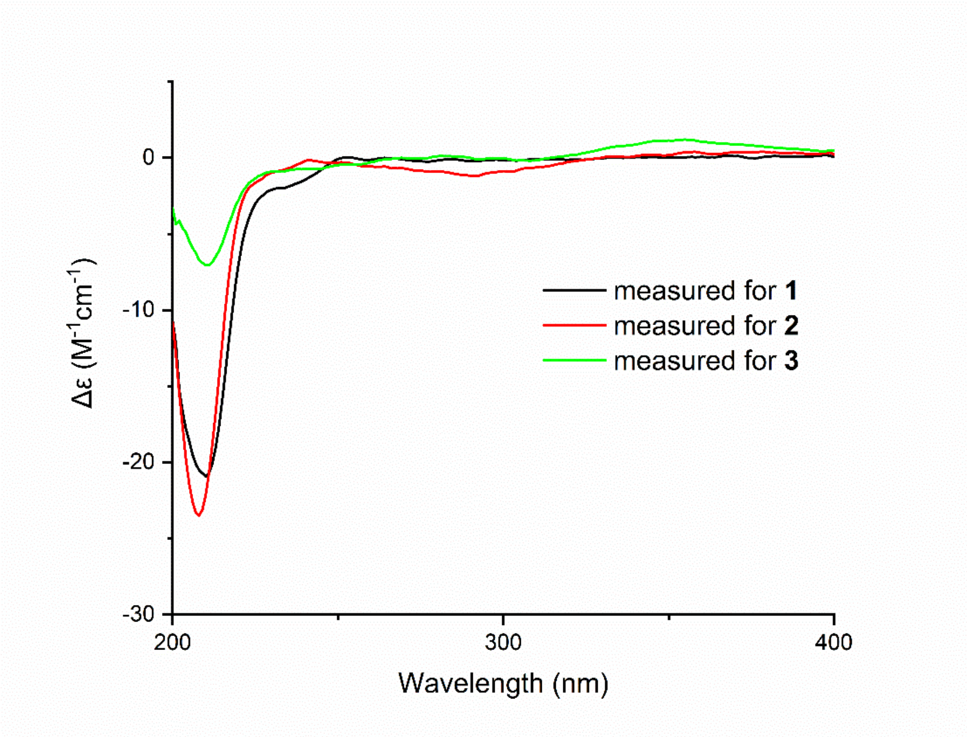

Figure S73. Experimental ECD spectra of compounds 1, 2 and 3.

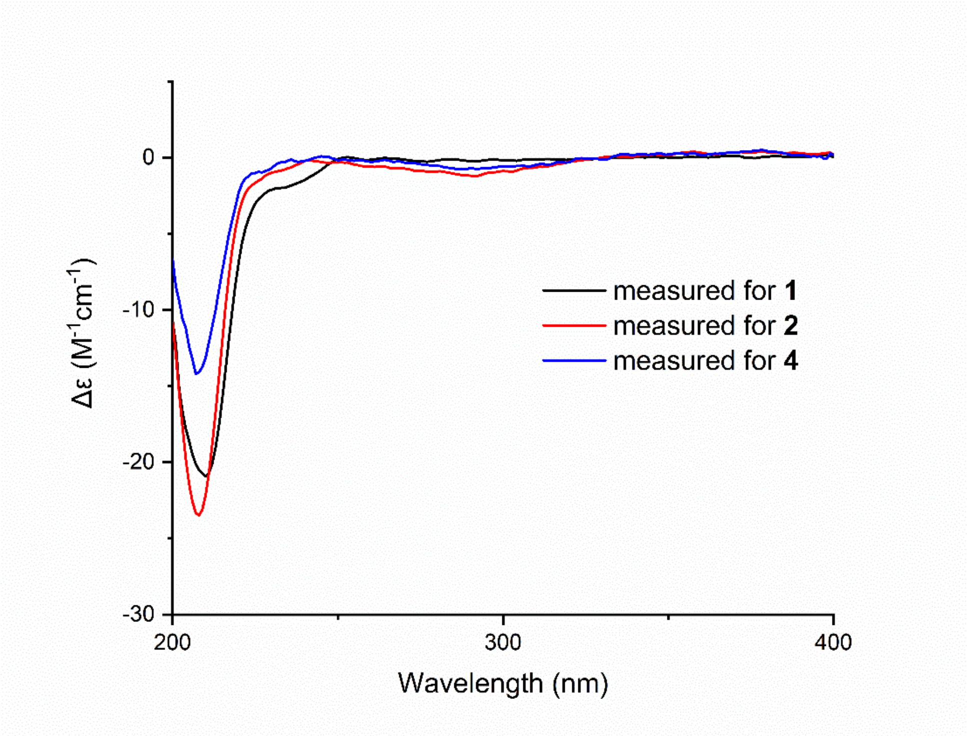

Figure S74. Experimental ECD spectra of compounds 1, 2 and 4.

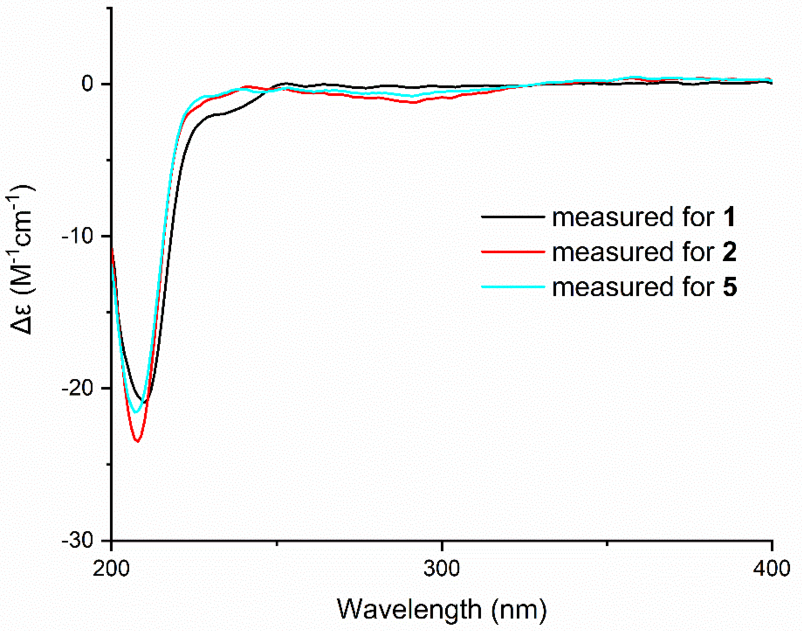

Figure S75. Experimental ECD spectra of compounds 1, 2 and 5.

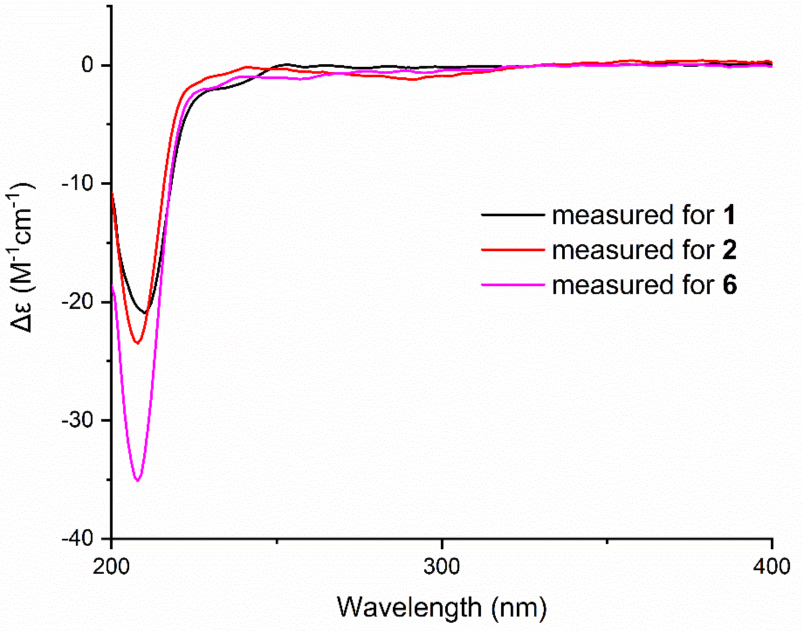

Figure S76. Experimental ECD spectra of compounds 1, 2 and 6.
